# Fabrication of Multifunctional Three‐Component Supramolecular Nano‐Biscuits via Two Macrocycles‐Involved Self‐Assembly for Rice, Citrus and Kiwifruit Protections

**DOI:** 10.1002/advs.202413826

**Published:** 2025-01-24

**Authors:** Xinyu He, Jinghan Yang, Xue Chen, Jiajia Chen, Haicong Zhao, Juan Liu, Fengpei Du, Peiyi Wang

**Affiliations:** ^1^ State Key Laboratory of Green Pesticide, Key Laboratory of Green Pesticide and Agricultural Bioengineering Ministry of Education, Center for Research and Development of Fine Chemicals of Guizhou University Guiyang 550025 China; ^2^ Department of Applied Chemistry, College of Science China Agricultural University Beijing 100193 China

**Keywords:** bacterial infections, biofilm barriers, enhanced efficacy, foliar adhesion, supramolecular nano‐biscuits

## Abstract

Bacterial plant diseases, worsened by biofilm‐mediated resistance, are increasingly threatening global food security. Numerous attempts have been made to develop agrochemicals that inhibit biofilms, however, their ineffective foliar deposition and difficulty in removing mature biofilms remain major challenges. Herein, multifunctional three‐component supramolecular nano‐biscuits (NI6R@CB[7]@*β*‐CD) are successfully engineered via ordered self‐assembly between two macrocycles [cucurbit[7]uril (CB[7]), *β*‐cyclodextrin (*β*‐CD)] and (*R*)‐2‐naphthol‐based bis‐imidazolium bromide salt (NI6R). This macrocycles‐involved bactericidal material combines many advantages. 1) Alleviate the off‐target movement of droplets on hydrophobic blade surfaces. 2) Enhance the biofilm‐disrupting ability. At a low‐dose of 4.44 *µg* mL^−1^, the inhibition rate of biofilm formation reached 78.3%. At 35.5 *µg* mL^−1^, the potency to remove mature biofilms reached 77.6%. 3) Efficiently hinder bacterial reproduction, swimming, extracellular polysaccharide production, extracellular enzyme secretion, and virulence to plants. These superior characteristics are undoubtedly transmitted to the in vivo control effect. At 200 *µg* mL^−1^, this smart material exhibits superior control efficiencies of 49.6%/65.0%/85.4% against three kinds of bacterial diseases (rice leaf blight, citrus canker, and kiwifruit canker), respectively, surpassing the commercial bactericide—thiodiazole‐copper‐20%SC (33.6%/41.5%/43.2%) and NI6R (40.3%/51.2%/71.2%). Furthermore, NI6R@CB[7]@*β*‐CD is biosafe to non‐target organisms. This study is instructive for constructing multifunctional agrochemicals in sustainable crop protection.

## Graphical abstract

Through supramolecular self‐assembly strategies, multifunctional three‐component nano‐biscuits (NI6R@CB[7]@*β*‐CD) are assembled from a chiral (*R*)‐2‐naphthol‐based bis‐imidazolium bromide salt (NI6R), cucurbit[7]uril (CB[7]) and *β*‐cyclodextrin (*β*‐CD). This material has several excellent functions: superior bacterial biofilm eradication, enhanced foliar deposition, and excellent in vivo control efficiency for rice, citrus, and kiwifruit protections.

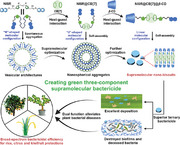

## 1 Introduction

Plant bacterial diseases have become a major challenge in agriculture, posing a major threat to crop safety, agricultural productivity, and economic stability.^[^ ^1^ ^]^ As a representative high‐risk bacterial disease, rice leaf blight (RLB) caused by *Xanthomonas oryzae* pv*. oryzae* (*Xoo*) is one of the three most destructive rice diseases (e.g., rice blast, rice sheath blight).^[^ ^2^ ^]^ Typically, *Xoo* infection leads to the formation of characteristic streaks on rice leaves, which in turn causes wilting and whitening, thereby severely affecting photosynthesis and plant normal growth.^[^ ^3^ ^]^ In severe outbreaks, this disease can cause a 30–50% reduction in rice yields.^[^ ^4^ ^]^ Currently, chemical control of RLB relies mainly on traditional bactericides such as bismerthiazol, zhongshengmycin and copper formulations. However, long‐term excessive use of these outdated agrochemicals has led to the rapid development of resistance in pathogenic bacteria, which not only reduces the practical efficacy, but also exposes agricultural production to higher disease risks.^[^ ^5^ ^]^ Unremitting efforts have found that the evolutionary bacterial biofilm plays a central role in the development of resistance. Biofilm, serving as a robust barrier, is mainly composed of extracellular polysaccharides (EPS), proteins, and phospholipids secreted by bacteria themselves.^[^ ^6^ ^]^ This intractable barrier not only weakens the penetration of biocides through physical isolation, but also provides a comfortable environment for bacteria to fight against other external interferences, including plant immune response and abiotic stress.^[^ ^7^ ^]^ The bacteria within the biofilm are a coordinated and cooperative community, which can quickly adapt to external pressure through mechanisms such as gene exchange and metabolic regulation, and significantly accelerate the emergence of bacterial resistance.^[^ ^8^ ^]^ This case greatly diminishes the effectiveness of conventional bactericides. However, an effective bactericide to inhibit or eradicate bacterial biofilms has not been reported in the field of agrochemicals so far. Therefore, the development of novel bactericides capable of dismantling biofilm defenses has become a key challenge in plant disease control.^[^ ^9^ ^]^

On the other hand, the low utilization rate of agricultural chemicals is another major factor limiting the actual efficacy of bactericides.^[^ ^10^ ^]^ In nature, the leaf surfaces of most crops are hydrophobic or superhydrophobic, which makes it difficult for pesticide droplets to effectively attach and spread on these interfaces.^[^ ^11^ ^]^ Taking rice as an example, the micro‐protruding structure and wax layer on the leaf surface significantly enhance the superhydrophobicity, resulting in the droplets being more likely to bounce and splash during contact.^[^ ^12^ ^]^ According to statistics, less than 10% of the pesticides sprayed in the field are effectively deposited on the target crops.^[^ ^13^ ^]^ This inefficient foliar deposition not only reduces the actual bactericidal effect, but also leads to a significant loss of pesticides to non‐target areas, seriously damaging ecosystems and non‐target organisms.^[^ ^14^ ^]^ Currently, a common method to improve foliar deposition is to introduce surfactants into the preparation of agrochemicals, which enhances the adhesion of droplets on hydrophobic leaf surfaces by reducing their dynamic surface tension.^[^ ^15^ ^]^ For example, the Tween series surfactants, as representatives of polysorbate esters (such as Tween‐20, Tween‐80), are widely used in pesticide formulations due to their good emulsifying, wetting and dispersing properties.^[^ ^16^ ^]^ However, the extensive use of various surfactants not only brings potential environmental pollution, but also makes it enter the food chain covertly, which seriously threatens human health. These issues have prompted the search for greener and more sustainable alternatives to minimize environmental impacts while maintaining pesticide effectiveness.^[^ ^17^ ^]^ In view of these challenges, the development of a highly biocompatible multifunctional bactericide that simultaneously addresses biofilm barriers and inefficient foliar deposition is particularly urgent.

Fortunately, the continuous advancement of supramolecular chemistry offers great potential for the development of multifunctional bactericides.^[^ ^18^ ^]^ Supramolecular systems integrate multiple molecules into a functional entirety through intermolecular noncovalent interactions, such as hydrogen bonding, host‐guest recognition, van der Waals forces, and hydrophobic interactions.^[^ ^19^ ^]^ This integration not only combines the advantages of multiple molecules, but also provides hierarchical aggregates with different topological shapes via ordered self‐assembly. Through this strategic optimization, the active ingredients involved in the assembly will obtain more advantageous properties, such as improved water solubility, better biocompatibility, and enhanced biological functions, which ultimately promote the bioavailability of bioactive substrates.^[^ ^20^ ^]^ In supramolecular optimization techniques, host‐guest recognition principle is widely adopted, mainly due to its privilege to effectively regulate the self‐assembly process to obtain the desired supramolecular assemblies.^[^ ^21^ ^]^ Macrocyclic host molecules usually have specific structural features, such as hydrophobic cavities or special charge distributions, which enable them to selectively bind to specific guest molecules.^[^ ^22^ ^]^ For instance, cucurbit[n]uril (CB[n]), as a typical biocompatible host molecule, with electron‐rich carbamido groups at its two portals, can form binary or ternary supramolecular complexes with cationic guest molecules through charge transfer, hydrogen bonding, and hydrophobic interactions.^[^ ^23^ ^]^ Similarly, cyclodextrins (CDs), as a class of macrocyclic oligosaccharides, can selectively encapsulate diverse lipophilic molecules within its specific hydrophobic cavity to form new supramolecular building blocks.^[^ ^24^ ^]^ Through these simple manipulations, it not only improves the stability of bioactive guest molecules,^[^ ^25^ ^]^ but also optimizes their physicochemical properties and biological functions.^[^ ^26^ ^]^ This case offers an opportunity to build multifunctional supramolecular materials in enhancing the droplet/leaf (liquid/solid) interface interaction and ultimate pesticide bioavailability.

To create a multipurpose supramolecular bactericide with anti‐biofilm, bactericidal, and foliar deposition properties for bacterial disease control, the conception of biologically active guest molecule is of primal importance.^[^ ^27^ ^]^ Naphthalene skeleton is an ideal candidate due to its derivatives having excellent antifungal and antibacterial properties as well as its ease of recognition by macrocyclic host molecules.^[^ ^28^ ^]^ Another active skeleton, cationic imidazolium salt, is known for its excellent chemical stability and broad‐spectrum antimicrobial activity, especially after the introduction of suitable long‐chain alkyl groups, whose bactericidal effect will be significantly enhanced.^[^ ^29^ ^]^ Meanwhile, the absolute chiral configuration (*R*‐ or *S*‐forms) in the molecular composition also plays a vital role in determining the final antimicrobial activity.^[^ ^30^ ^]^ Benefiting from these revelations, we expect to conceive a kind of bioactive chiral molecule with both naphthyl and imidazolium skeletons linked by alkyl chains. The synergistic effect of those bioactive fragments will endow this designed guest molecule with good antibacterial activity and also lay a solid foundation for the construction of multifunctional supramolecular bactericides.

**Scheme 1:**
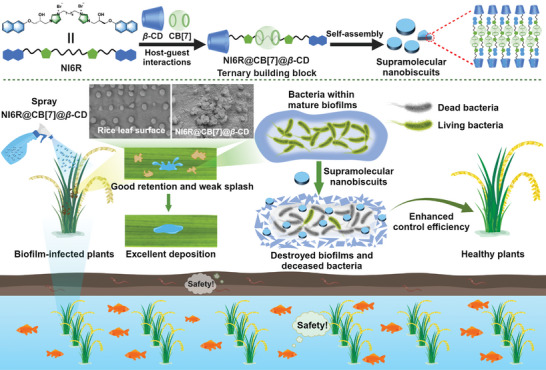
The illustration presents the assembly of a three‐component supramolecular nano‐biscuit system, designed as a biosafe, multifunctional bactericidal material aimed at enhancing foliar droplet deposition, removing persistent biofilms, and effectively combating bacterial diseases.

In this study, we have devised two chiral structures and screened out a highly bioactive (*R*)‐2‐naphthol‐based bis‐imidazolium bromide salt (NI6R) as the guest molecule. Later, the biocompatible macrocyclic host molecules—cucurbit[7]uril (CB[7]) and *β*‐cyclodextrin (*β*‐CD) were used to regulate and optimize the physicochemical property, self‐assembly behavior, and biological function of NI6R via host‐guest encapsulating forces, thereby affording a ternary supramolecular building block (NI6R@CB[7]@*β*‐CD), which spontaneously self‐assembled into three‐component supramolecular nano‐biscuits as multifunctional bactericidal materials (**Scheme** **1**). This macrocycles‐involved material reduces the off‐target movement of droplets on hydrophobic leaf surfaces, enhances the inhibiting and eradicating ability of biofilm barriers, and hinders the bacterial virulence to plants. These superior advantages ultimately improve the control efficacy of three kinds of high‐risk bacterial diseases (rice leaf blight, citrus canker, and kiwifruit canker) in pot experiments. Furthermore, NI6R@CB[7]@*β*‐CD shows good biosafety against non‐target organisms such as rice seeds, rice plants, zebrafishes, and earthworms. Overall, this study provides a new research idea for the design and application of multifunctional supramolecular bactericides to efficiently control bacterial diseases without introducing hazardous additives in pesticide formulations.

## 2 Result and Discussion

### 2.1 Synthesis and In Vitro Bactericidal Assay of Two Chiral Molecules (NI6R and NI6S)

According to the planned molecular design concept, a simple and efficient synthesis route is used to construct the desired chiral molecules. Moreover, all the used chemical reagents including 2‐naphthol, *R*‐epichlorohydrin, imidazole, 1,6‐dibromohexane, cucurbit[7]uril and *β*‐CD are readily available commercial reagents. As shown in Figure S1 (Supporting Information), the substitution reaction between 2‐naphthol and *R‐* or *S*‐epichlorohydrin (puriss., ≥ 99%) was carried out to give two chiral intermediates owning an ethylene oxide group, which were then ring‐opened by imidazole under alkaline conditions to provide two key intermediates (*R*‐NI and *S*‐NI) with a chiral isopropanol substructure. Finally, a classical synthetic method of quaternary ammonium salt between 1,6‐dibromohexane and intermediate *R*‐NI (or *S*‐NI) was adopted to afford the desired chiral molecules (NI6R and NI6S) with a high yield of 84.7–88.9%. Their molecular compositions were successfully verified by NMR, HPLC, and HRMS spectra (Figures S2–S8, Supporting Information). The in vitro bactericidal potency of NI6R and NI6S was determined by the frequently‐used turbidimetric method.^[^ ^31^ ^]^ Evidently, the two designed chiral molecules exhibit strong bioactivity against pathogenic *Xoo* strains, achieving complete inhibition at 50, 25, and 12.5 *µg* mL^−1^ (**Table** **1**; Table S1, Supporting Information). Further screening reveals significantly lower EC₅₀ values of 1.11 *µg* mL^−1^ (NI6R) and 3.78 *µg* mL^−1^ (NI6S), outperforming commercial bactericides such as TC (EC_50_ = 47.5 *µg* mL^−1^) and BT (EC_50_ = 32.6 *µg* mL^−1^). This delightful outcome validates our initial conception in obtaining highly active bactericidal molecules. Careful observation revealed that the bactericidal activity of *R*‐type molecule (NI6R) was 3.4 times to that of *S*‐type molecule (NI6S), therefore, NI6R was selected as the guest molecule to construct macrocycles‐involved functional supramolecular materials.

**Table 1:** In vitro bactericidal activity of compounds NI6R and NI6S against *Xoo*.

Compounds	Inhibition ratio (%)		Regression equation	EC_50_ ^a)^ (*µg* mL^−1^)	R^2^
	50 *µg* mL^−1^	25 *µg* mL^−1^			
NI6R	100	100	y = 7.81x + 4.63	**1.11 ± 0.06**	0.966
NI6S	100	100	y = 4.07x + 2.57	3.78 ± 0.48	0.967
BT	82.5 ± 4.1	28.3 ± 1.9	y = 4.99x – 2.56	32.6 ± 1.7	0.927
TC	51.5 ± 1.5	30.9 ± 2.5	y = 1.77x + 2.04	47.5 ± 2.4	0.999

^a)^ EC_50_ values of bactericidal activity are expressed as mean ± SD (standard deviation).

### 2.2 Successful Preparation of Three‐Component Supramolecular Nano‐Biscuit Materials NI6R@CB[7]@*β*‐CD

Combined with the structural characteristics of NI6R, the hexyl linker within the two cationic imidazolium tends to be favored and encapsulated by CB[7], whereas the naphthyl ether substructure tends to be recognized and coated by *β*‐CD.^[^ ^32^ ^]^ Thus, the two macrocyclic molecules (CB[7] and *β*‐CD) can be flexibly employed to optimize the physicochemical and biological properties of guest molecule (NI6R). On this basis, a ternary supramolecular material (NI6R@CB[7]@*β*‐CD) can be constructed through the stepwise encapsulation of NI6R by CB[7] and *β*‐CD. Briefly, NI6R (4.0 µL, 64.1 mm) dissolved in dimethyl sulfoxide (DMSO) was dropped into 0.992 mL deionized aqueous solution containing CB[7] (0.256 mm). After ultrasonic oscillation for 15 mins at room temperature, the turbid system became a transparent solution, indicating the formation of binary supramolecular complex (NI6R@CB[7]). Later, *β*‐CD (4.0 µL, 64.1 mm) dissolved in deionized water was supplemented into the above solution. After fully mixing for 5 mins at room temperature, a three‐component supramolecular material (NI6R@CB[7]@*β*‐CD, molar ratio, 1:1:1) was fabricated, in which the effective concentration of NI6R is 0.256 mm (200 *µg* mL^−1^) and the fraction of DMSO is 0.4% (V/V). During the investigation, techniques such as UV–vis titration, Job's plot analysis, ^1^H NMR titration, Zeta potential, HRMS determination, and scanning electron microscopy (SEM) were employed to thoroughly examine the stoichiometric ratios, driving forces, microscopic structure, and self‐assembly mechanisms.

Given that the successful formation of binary complex (NI6R@CB[7]) is a key stage for constructing the final three‐component supramolecular materials, thus, the stoichiometric ratio and driving force were first investigated. Through the UV–vis titration experiment of NI6R with CB[7] (0–2.0 equivalents), the absorbance curve of NI6R was gradually decreased until the amount of CB[7] reaches 1.0 equivalent (Figure S9A, Supporting Information). Illustratively, the absorbance at 272 nm was reduced to an equilibrium point at 1.0 (Figure S9B, Supporting Information), indicating a one‐to‐one binding mode between NI6R and CB[7]. This assertion was further confirmed by Job's plots test (Figure S9C, Supporting Information) and their HRMS spectrum (Figure S9D, Supporting Information), giving the optimal fraction of 0.5 ([NI6R]/[NI6R+CB[7]]) and a merged molecular weight of 891.3377 for NI6R@CB[7], respectively. The possible packaging location was further explored by ^1^H NMR titration. As shown in Figure S9E and Table S2 (Supporting Information), upon the addition of 1.0 equivalent CB[7], the protons (H_m_, H_o_, H_p_) from the hexyl linker had a larger chemical shift (Δ*δ* = −0.552, −0.297, −0.146 ppm, respectively) to upfield, revealing that CB[7] potentially encapsulated the alkyl chain between the two imidazolium bromides of NI6R, causing the shielding effect. At the same time, the proton signals of H_a_ and H_h_ from the cationic imidazolium exhibited downfield shifts, with Δ*δ* values of 0.079 and 0.036 ppm, respectively. This indicates that these protons were positioned at external ports of CB[7], resulting in a deshielding effect. These findings confirm that CB[7] selectively encases the hexyl chain of NI6R, forming a 1:1 complex. Similarly, after introducing the *β*‐CD into NI6R, the proton signals from the naphthyl ether substructure exhibited obvious chemical shifts (e.g., Δ*δ* for H_b‐c_, H_d_, H_f_, H_g_, H_i_, and H_j_ was ‐0.022, ‐0.011, ‐0.020, ‐0.030, 0.026 and 0.019 ppm, respectively, molar ratio NI6R:*β*‐CD = 1:1), while other remaining protons did not change significantly (Figure S10A and Table S3, Supporting Information), indicating the selective encapsulation of naphthalene ring inside the hydrophobic *β*‐CD's cavity. The Job's plot analysis (Figure S10B, Supporting Information) and the molecular weight of 877.5825 obtained from the HRMS spectrum (Figure S10C, Supporting Information) confirm a 1:1 binding interaction between NI6R and *β*‐CD. These preliminary findings provide a solid basis for developing three‐component supramolecular complexes.

**Figure 1:**
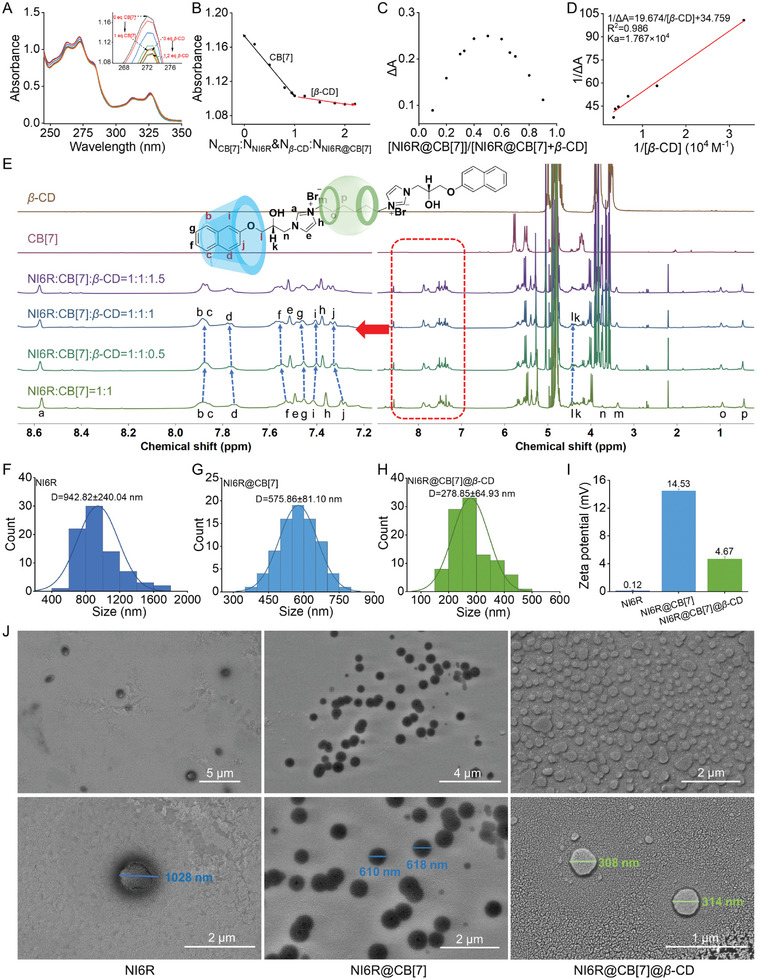
Characterization of NI6R@CB[7]@*β*‐CD. A) UV–vis spectra of NI6R (0.15 mm) following incremental addition of CB[7] (0.0–0.15 mm) and *β*‐CD (0.0–0.18 mm) in water. B) UV–vis absorption at 272 nm for NI6R (0.15 mm) upon varying the amounts of *β*‐CD and CB[7]. C) Job's plot for ΔA at 272 nm with a combined concentration of 0.15 mm for NI6R@CB[7] and *β*‐CD in water. D) Benesi‐Hildebrand fitting curves for the colorimetric [ΔA] ^−1^ data. E) ^1^H NMR spectra of NI6R@CB[7] (2.0 mm, C_NI6R_: C_CB[7]_ = 1:1) with varying molar ratios of *β*‐CD (1:0.5, 1:1, 1:1.5) in D_2_O. F‐H) Particle size distribution of NI6R, NI6R@CB[7], and NI6R@CB[7]@*β*‐CD at 100 µg mL^−1^. I) Zeta potentials of NI6R, NI6R@CB[7], and NI6R@CB[7]@*β*‐CD. J) SEM images of NI6R, NI6R@CB[7], and NI6R@CB[7]@*β*‐CD at 100 *µg* mL^−1^.

The assembling process of NI6R@CB[7]@*β*‐CD was primarily monitored by UV–vis titration of NI6R@CB[7] with different amounts of *β*‐CD. Clearly, the absorption peak at 272 nm continued to give a downward trend with the stepwise addition of *β*‐CD (**Figure** **1**A), which may be attributed to the occurring envelopment of naphthyl ring by *β*‐CD, thereby affecting the absorbance. When the amount of *β*‐CD reached 1.0 equivalent, the changes in absorbance stabilized (Figure 1B), indicating that NI6R@CB[7] and *β*‐CD assembled to the ternary system with a possible 1:1 stoichiometric ratio. The subsequent Job's plots test validates this inference, in which a fraction of 0.5 (NI6R@CB[7]/(NI6R@CB[7]+*β*‐CD)) at the maximum absorption change (ΔA) was observed (Figure 1C). Additionally, the binding constant of 1.767 × 10^4^ M^−1^ (Figure 1D) indicates the formation of a stable three‐component supramolecular assembly. Their HRMS spectrum offers a combined molecular weight of 973.2930 (m/z) for NI6R@CB[7]@*β*‐CD (Figure S11, Supporting Information), verifying a suitable binding mode of 1:1:1 for the three components. Next, the packaging sites between *β*‐CD and NI6R@CB[7] were also probed through ^1^H NMR titration experiments. As illustrated in Figure 1E and Table S4 (Supporting Information), with the addition of 1.0 equivalent *β*‐CD into NI6R@CB[7], except for the chemical shift change of the protons on the naphthalene ring (e.g., Δ*δ* for H_f_ and H_g_ was 0.029 and 0.006 ppm, respectively), the other protons from the imidazole or alkyl chain did not change markedly. This finding illustrates the *β*‐CD‐involved host–guest interaction was mainly happened at the naphthyl ether moiety of NI6R, confirming the selective capsulation. Beyond that, the 2D ROESY and 2D DOSY spectra of NI6R, NI6R@CB[7] and NI6R@CB[7]@*β*‐CD were performed and illustrated in Figures S12–S14,S15–S17 (Supporting Information), respectively. From the 2D ROESY spectrum of NI6R, the protons (H_m_, H_o_, H_p_) from the hexyl group have associated signals with naphthalene ring, indicating that these hydrophobic groups are close to each other in the water environment, thereby adapting an “M”‐shaped molecular configuration (Figures S12 and S18A, Supporting Information). Based on the 2D ROESY spectrum of NI6R@CB[7], the protons (H_m_, H_o_, H_p_) from the hexyl group have associated signals with CB[7] (Figure S13, Supporting Information), confirming the formation of binary supramolecular complexes (NI6R@CB[7]). From the 2D ROESY spectrum of NI6R@CB[7]@*β*‐CD, there were obvious associated signals for both the protons (H_m_, H_o_, H_p_) from the hexyl group with CB[7] and the protons from the naphthalene ring with *β*‐CD (Figure S14, Supporting Information), verifying the formation of ternary supramolecular complexes (NI6R@CB[7]@*β*‐CD). Moreover, The diffusion coefficient (*D*) values of NI6R, NI6R@CB[7] and NI6R@CB[7]@*β*‐CD were 2.88 × 10^−10^ m^2^ s^−1^, 2.34 × 10^−10^ m^2^ s^−1^, and 1.91 × 10^−10^ m^2^ s^−1^, respectively (Figures S15–S17, Supporting Information). The gradual reduction in diffusion coefficient values indicates the formation of supramolecular complexes.^[^ ^33^ ^]^

Subsequently, the particle size distribution, Zeta potential, and topological morphology of these aggregates were measured by dynamic light scattering (DLS) and SEM. Intriguingly, the average particle size of NI6R@CB[7]@*β*‐CD assemblies was 278.85 nm, notably smaller than the 575.86 nm of the NI6R@CB[7] complex and the 942.82 nm of the NI6R alone (Figure 1F–H). This outcome manifests that the ternary supramolecular aggregate (NI6R@CB[7]@*β*‐CD) has better dispersibility within the system. Regarding the surface charge of the nanoparticles in aqueous solution, the NI6R itself gave a Zeta potential of 0.12 mV, while NI6R@CB[7] presented an elevated value of 14.53 mV (Figure 1I). This consequence may be attributed to the formation of a steric “U”‐shaped host‐guest building unit, in which two hydrophilic cationic imidazolium salts located at the CB[7] ports are more likely to expose to the surface of the nanoparticles during self‐assembly in an aqueous environment (Figure S19B, Supporting Information), thereby causing the enhancement of Zeta potential. Upon further addition of *β*‐CD, the Zeta potential of the resulting NI6R@CB[7]@*β*‐CD complex decreased to 4.67 mV compared to NI6R@CB[7]. This reduction may ascribe to the selective encapsulation of the hydrophobic naphthyl group in the NI6R structure by the externally hydrophilic *β*‐CD, effectively shielding the surface charge of the complex. These findings further corroborate the successful construction of the ternary supramolecular assembly. SEM characterization (Figure 1J) reveals that NI6R exhibits a vesicular structure, while NI6R@CB[7] appears as regular spherical aggregates. Remarkably, the final two macrocycles‐involved ternary building block (NI6R@CB[7]@*β*‐CD) self‐assembles into nanoscale biscuits.

Considering the anisotropy and molecular structural characteristics of these new supramolecular building blocks (NI6R, NI6R@CB[7], and NI6R@CB[7]@*β*‐CD), their possible assembly mechanisms are proposed as follows. For the NI6R itself, it is a typical supramolecular amphiphile, which probably adapts an “M”‐shaped molecular configuration. This inference was confirmed by its optimal molecular configuration using Chem 3D (Figure S18A, Supporting Information) and ^1^H‐^1^H ROESY spectrum (Figure S12, Supporting Information). Moreover, the concentration‐dependent UV spectra showed that the absorption peak of the aromatic region did not shift (Figure S18B, Supporting Information), indicating a spontaneous assembly process. Later, this building unit will voluntarily aggregate together to arrange the hydrophobic naphthyl and hexyl groups inside the assembly, and subsequently tends to give vesicular architectures (Figure S19A, Supporting Information). After loading NI6R with CB[7], a new binary host‐guest building unit (NI6R@CB[7]) adapts a “U”‐shaped molecular configuration along with a steric and hydrophilic imidazolium‐CB[7]‐imidazolium moiety, which will prefer to be exposed to the water environment (Figure S19B, Supporting Information). Then, NI6R@CB[7] starts to assemble, allowing the hydrophilic part to contact the water environment and the hydrophobic naphthyl part to extend into the interior. This scenario tends to promote the formation of a curved, layered structure that minimizes the exposure of hydrophobic regions to the aqueous environment. Finally, through the hydrogen bonding interaction between imidazolium salts, water molecules, bromide ions, etc., the layer and layer structure are connected to form spherical aggregates. After continuing to introduce *β*‐CD to encapsulate the naphthalene ring of NI6R@CB[7], this three‐component building block (NI6R@CB[7]@*β*‐CD) adapts a linear molecular configuration and assembles to form a layered structure via a staggered arrangement. This case not only protects the hydrophobic naphthyl group inside, but also allows the large volume of the hydrophilic *β*‐CD to contact the water environment. Finally, through the multiple hydrogen bonding between hydroxyl groups, ether groups, urea groups, water molecules, etc., all the layers are connected to each other to give nanoscale biscuits (Scheme 1).

Considering that loading NI6R with CB[7] and *β*‐CD combines the potential advantages of the two macrocycles, such as biocompatibility, we speculate that it will improve biological properties, biosafety, and foliar deposition performance in pesticide spraying and utilization.

### 2.3 NI6R@CB[7]@*β*‐CD Exhibits Enhanced Inhibitory Activity Against Bacterial Biofilm Barriers

**Figure 2:**
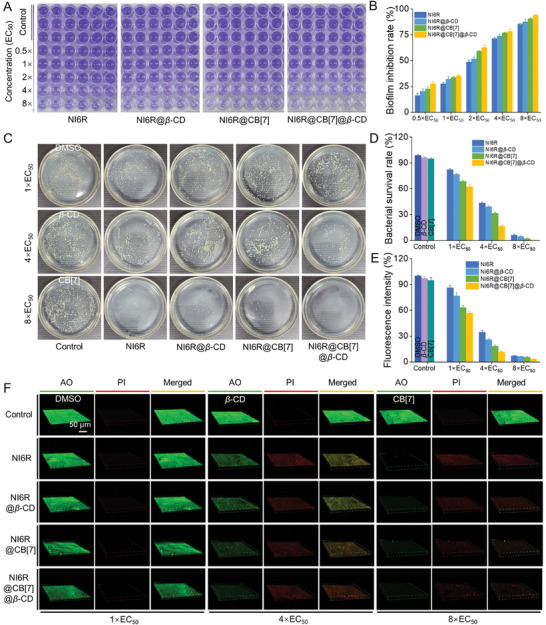
A) Biofilm inhibition results using crystal violet staining after 48 h of co‐incubation with varying doses of NI6R, NI6R@*β*‐CD, NI6R@CB[7], and NI6R@CB[7]@*β*‐CD. Control groups included DMSO (0.18%, V/V), *β*‐CD, and CB[7]. B) Biofilm inhibition rates following treatment with different concentrations (0.5–8.0×EC_50_) of NI6R, NI6R@*β*‐CD, NI6R@CB[7], and NI6R@CB[7]@*β*‐CD. C) The resulting *Xoo* colonies within biofilms grown on solid NB medium at varying doses of the treatments. D) Statistical survival rates of *Xoo* colonies on agar plates. E) Analysis of relative green fluorescence intensity from CLSM 3D images using ImageJ software. F) CLSM 3D images of *Xoo* biofilms stained with AO (live cells, green) and PI (dead cells, red) after 48 h of co‐incubation with different doses of NI6R, NI6R@β‐CD, NI6R@CB[7], and NI6R@CB[7]@*β*‐CD.

A key prerequisite for a superior bactericide to achieve good bactericidal effect is to break through the stubborn biofilm barrier, which is secreted and constructed by bacteria themselves.^[^ ^34^ ^]^ To verify the inhibitory ability of NI6R@CB[7]@*β*‐CD on *Xoo*’s biofilms, the crystal violet staining method was carried out to quantify the biofilm production.^[^ ^35^ ^]^ Following 48 h of co‐incubation with varying concentrations of NI6R@CB[7]@*β*‐CD, this three‐component supramolecular material exhibited a concentration‐dependent anti‐biofilm activity. As observed from **Figure** **2**A, the purple solution gradually became fainter with increasing their concentrations. The corresponding OD_570_ _nm_ value was detected in Figure S20 (Supporting Information) to calculate the inhibition rate of biofilm formation. Clearly, at a low concentration of 2×EC_50_ (2.22 *µg* mL^−1^), NI6R@CB[7]@*β*‐CD demonstrated a 62.8% inhibition of biofilm formation, surpassing NI6R (48.8%), NI6R@*β*‐CD (51.8%), and NI6R@CB[7] (59.2%) under the same conditions (Figure 2B). Increasing the concentration of NI6R@CB[7]@*β*‐CD to 4× and 8×EC_50_ (4.44 and 8.88 *µg* mL^−1^) resulted in anti‐biofilm rates of 78.3% and 94.0%, respectively. This effectiveness was markedly superior to NI6R alone (71.4% and 85.6%, respectively), revealing a highly effective biofilm inhibitor (NI6R@CB[7]@*β*‐CD) was discovered by utilizing the host‐guest supramolecular optimization strategy. Besides, the effects of BT and TC on biofilm formation were almost negligible, even at a higher concentration of 8.88 *µg* mL^−1^ (Figure S21, Supporting Information).

To investigate the survival rate of *Xoo* strains within the biofilm, the associated bacterial monoclonal culture on solid NB (nutrient broth) medium was performed. As shown in Figure 2C,D, compared to the controls (CB[7] and *β*‐CD), the NI6R@CB[7]@*β*‐CD treatment group exhibited the lowest survival rates, at 16.7% and 0.36% for concentrations of 4.44 and 8.88 *µg* mL^−1^, respectively. These values were significantly lower than those of monomeric NI6R (43.6% and 6.30%, respectively), NI6R@*β*‐CD (39.1% and 4.65%, respectively), and NI6R@CB[7] (31.5% and 2.13%, respectively). These outcomes disclose that NI6R@CB[7]@*β*‐CD has a dual function to disrupt the biofilm formation and the bacterial propagation within the biofilm. The following confocal laser scanning microscopy (CLSM) was used to visualize the anti‐biofilm property, in which two dyes including acridine orange (AO) and propidium iodide (PI) were employed to label the live (green fluorescence) and dead *Xoo* cells (red fluorescence), respectively. As illustrated in Figure 2E,F, the control groups (0.18% DMSO, *β*‐CD, and CB[7]) gave strong green fluorescence and almost no red fluorescence, indicating that a large number of live *Xoo* cells and dense biofilms existed. With the increase of dosage in each treatment group, the green fluorescence presented a gradual weakening trend. Especially at 4× and 8×EC_50_, NI6R@CB[7]@*β*‐CD exhibited the lowest green fluorescence intensity compared to other treatment groups (NI6R, NI6R@*β*‐CD, NI6R@CB[7], Figure 2E). Combined with their pronounced inhibitory effects on biofilm formation, biofilm‐enclosed bacteria represent only a small fraction, thereby resulting in a significant reduction in the number of *Xoo* within the biofilm. Moreover, at the dose of 8×EC_50_, NI6R, NI6R@*β*‐CD, NI6R@CB[7], and NI6R@CB[7]@*β*‐CD displayed good bactericidal properties, thus, the green/red fluorescence for living/dead *Xoo* cells was weak (Figure S22, Supporting Information). This outcome was consistent with the crystal violet staining findings, indicating that the co‐assembled three‐component supramolecular material was an optimal biofilm inhibitor.

### 2.4 NI6R@CB[7]@*β*‐CD can Eradicate the Already Established Biofilms

**Figure 3:**
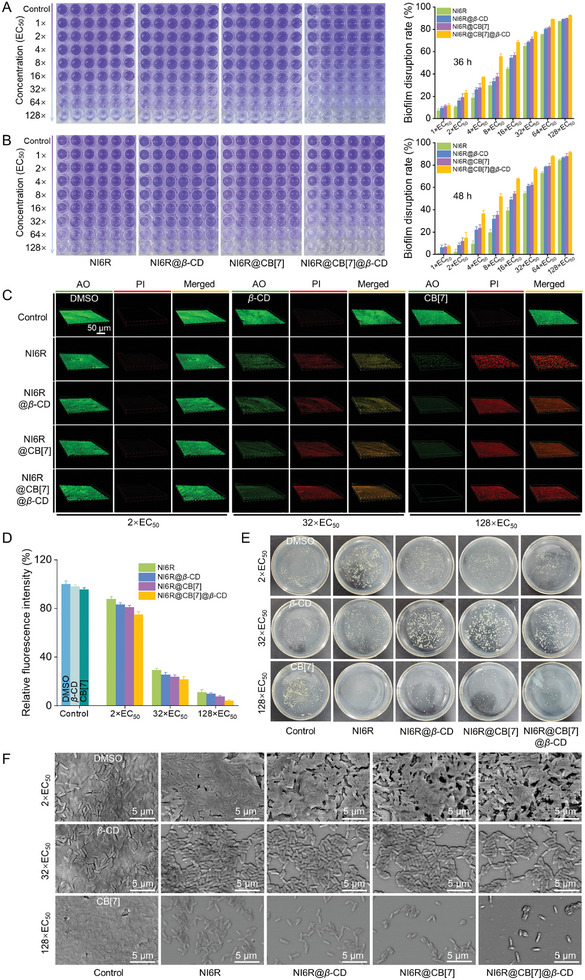
A,B) Crystal violet staining used to assess the biofilm eradication effects of various agents: *Xoo* biofilms were established in 96‐well plates for 36 or 48 h, followed by exposure to different doses of NI6R, NI6R@*β*‐CD, NI6R@CB[7], and NI6R@CB[7]@*β*‐CD for an additional 24 h before staining. Biofilm destruction rates for these treatments are shown in the histograms on the right. C) CLSM 3D images of *Xoo* biofilms pre‐formed in 12‐well plates for 48 h, then treated with varying doses of supramolecular complexes for 24 h, followed by AO and PI co‐staining. D) Analysis of relative green fluorescence intensity from CLSM 3D images using ImageJ. E) *Xoo* colony cultures within biofilms grown on agar plates. F) SEM images of *Xoo* biofilm disruption by supramolecular materials after 48 h of biofilm formation, followed by 24 h of treatment with different doses of the materials before SEM imaging.

The outbreak of bacterial diseases often means that a large number of stubborn mature biofilms are established by pathogenic bacteria, which consequently serves as a strong barrier to hinder the penetration and action of conventional bactericides.^[^ ^36^ ^]^ Therefore, it is crucial to explore its ability to eradicate mature biofilms in practical applications. In this study, the mature biofilm was pre‐established via statically culturing the *Xoo* cells for 36 or 48 h (Figure S23, Supporting Information). Later, different doses of supramolecular materials were added to co‐incubate with mature *Xoo*‐biofilms for another 24 h to allow them to dismantle the biofilm. Finally, the crystal violet staining with the absorbance at OD_570_ _nm_ was tested for quantitative biofilm analysis. As illustrated in **Figures** **3**A,B and S24 (Supporting Information), compared with control groups (0.18% DMSO, *β*‐CD, and CB[7]), the purple color solution gradually became lighter as the concentration of active ingredient increased, indicating that the treatment groups could destroy the preformed biofilm in a concentration‐dependent manner. For the biofilm incubated for 36 h, the eradication rates of NI6R@CB[7]@*β*‐CD at 8×, 16×, 32×, 64×EC_50_ were 55.8%, 68.6%, 77.6%, and 89.0%, respectively, obviously exceeding the NI6R alone (29.7%, 44.7%, 65.0%, and 75.2%, respectively), as well as NI6R@*β*‐CD and NI6R@CB[7] at the same conditions. For the biofilm aged for 48 h, the eradication rates of NI6R@CB[7]@*β*‐CD at 8×, 16×, 32×, 64×EC_50_ were 51.9%, 67.9%, 76.8%, and 87.8%, respectively, which were also better than those of NI6R itself (19.6%, 39.3%, 54.7%, and 72.8%, respectively), NI6R@*β*‐CD (31.8%, 49.0%, 61.5%, and 78.2%, respectively), and NI6R@CB[7] (35.5%, 54.5%, 62.5%, and 79.0%, respectively). Remarkably, NI6R@CB[7]@*β*‐CD exhibits the most superior biofilm eradication ability, verifying that two‐macrocycles‐involved molecular optimization can enhance the biological function of bioactive substrates (NI6R). Besides, the commercial bactericides TC and BT had a weak ability to eradicate mature biofilms, even at very high concentrations (Figure S25, Supporting Information). For instance, regarding to the pre‐established biofilm aged for 48 h, at 71.04 *µg* mL^−1^ (64×EC_50_) and 142.08 *µg* mL^−1^ (128×EC_50_), TC afforded the eradication rates of 1.81% and 2.25%, respectively. Meanwhile, BT gave the corresponding eradication rates of 14.92% and 22.81%, which were much lower than those of our designed bactericides. The above outcome substantiates that traditional agricultural bactericides have weak biofilm scavenging ability.

Subsequently, CLSM imaging was used to visualize the eradication effect. In this experiment, *Xoo* strains were statically cultured in a 12‐well plate for 48 h to produce the mature biofilm. Then, different concentrations of NI6R@CB[7]@*β*‐CD or other components (NI6R, NI6R@*β*‐CD, NI6R@CB[7]) were added and co‐incubated for another 24 h. After that, all the samples were consecutively stained with AO and PI before CLSM imaging. As displayed in Figure 3C,D, at 32×EC_50_ (35.52 *µg* mL^−1^), the green fluorescence intensity in treatment groups weakened, while the red fluorescence become stronger compared with the controls (0.18% DMSO, *β*‐CD, and CB[7]). This phenomenon indicates that the dense biofilm is disintegrated, and a certain number of bacteria within the biofilm are killed. When the concentration increased to 128×EC_50_, the intensity of green fluorescence was drastically reduced in the NI6R, NI6R@*β*‐CD, NI6R@CB[7], and NI6R@CB[7]@*β*‐CD treatment groups, corresponding to a reduction of 88.8%, 90.0%, 92.4%, and 95.6%, respectively, suggesting that there are only a few living bacteria within the biofilm. These intriguing outcomes reveal that NI6R@CB[7]@*β*‐CD can efficiently disrupt the biofilm, thus achieving the effective annihilation of pathogens. This statement was further confirmed by plate diffusion method (Figure 3E; Figure S26, Supporting Information). Compared with the control, the number of *Xoo* colonies from the biofilm was significantly reduced after NI6R, NI6R@*β*‐CD, NI6R@CB[7] and NI6R@CB[7]@*β*‐CD treatments. At 32× and 128×EC_50_, the bacterial survival rates of NI6R@CB[7]@*β*‐CD were 30.6% and 2.42%, respectively, which were lower than those of NI6R, NI6R@*β*‐CD, and NI6R@CB[7], confirming the most effective for the three‐component supramolecular material. Certainly, SEM‐assisted imaging was further executed to directly observe the degree of damage to mature biofilms. As shown in Figure 3F, at 2×EC_50_, the pre‐established biofilm was slightly thinned and cracked. Whereas, this damage was significantly enhanced when the concentration was increased to 32×EC_50_, causing a large number of bacteria reduced and exposed to the outside. As the concentration reached 128×EC_50_, the biofilm was almost completely destroyed and few bacteria survived. By comparison, it is found that the NI6R@CB[7]@*β*‐CD treatment groups exhibit the best biological properties, verifying the feasibility of using supramolecular technology to optimize active small molecules. The above test results show that the constructed three‐component supramolecular aggregates are able to effectively eradicate biofilm and kill encapsulated bacteria, which is of great significance for the upgrading of bactericides in the field of agriculture.

### 2.5 The Anti‐Biofilm Mechanism and Other Functions of NI6R@CB[7]@*β*‐CD

**Figure 4:**
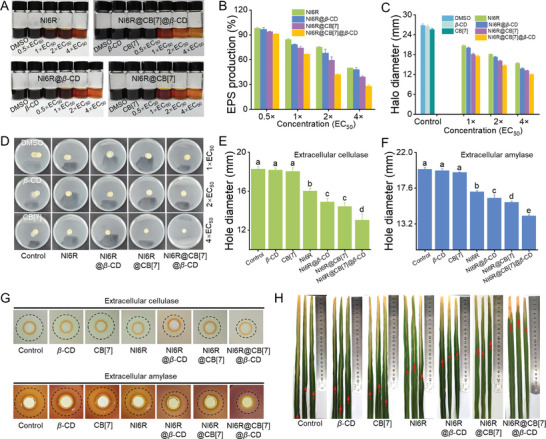
A) Quantitative analysis of EPS production at different doses (1×, 2×, 4×EC_50_) of NI6R, NI6R@*β*‐CD, NI6R@CB[7] and NI6R@CB[7]@*β*‐CD by the phenol‐sulfuric acid method. B) The relative EPS yields affected by NI6R, NI6R@*β*‐CD, NI6R@CB[7], and NI6R@CB[7]@*β*‐CD at different doses. C,D) Determination of swimming motility and colony circular diameter of *Xoo* strains after treatment with different doses (1×, 2×, 4×EC_50_) of NI6R, NI6R@*β*‐CD, NI6R@CB[7] and NI6R@CB[7]@*β*‐CD. E,F) Aperture diameters of extracellular cellulase and amylase treated with NI6R, NI6R@*β*‐CD, NI6R@CB[7] and NI6R@CB[7]@*β*‐CD at 4×EC_50_. G) Photographs for extracellular cellulase and amylase experiments. H) Representative susceptible rice leaves treated with NI6R, NI6R@*β*‐CD, NI6R@CB[7] and NI6R@CB[7]@*β*‐CD at 4×EC_50_ by the leaf‐clipping method. Single‐factor ANOVA test was used, with lower case letters indicating statistically significant differences between components (*p* < 0.05 adjusted by Waller–Duncan method).

To explore the possible anti‐biofilm mechanism, the influence of NI6R@CB[7]@*β*‐CD on the production of extracellular polysaccharides (EPS), which are a key component of biofilm barrier, was detected by the classical phenol‐sulfuric acid approach.^[^ ^37^ ^]^ As illustrated in **Figure** **4**A, the color of the EPS extract shifted from dark to orange‐yellow with increasing the concentration of active ingredients. To quantify the amounts of EPS products, the OD_490_ _nm_ was monitored to calculate the final contents in each specimen (Figure 4B; Figure S27, Supporting Information). At a low dose of 2×EC_50_ (2.22 *µg* mL^−1^), the EPS production rates for NI6R@CB[7]@*β*‐CD, NI6R@*β*‐CD, NI6R@CB[7], and NI6R were 42.1%, 59.5%, 68.2%, and 75.2%, respectively. Clearly, NI6R@CB[7]@*β*‐CD exhibited the most effective inhibition of EPS production, which was significantly better than NI6R alone, further confirming the effectiveness of the supramolecular optimization strategy. When the concentration was increased to 4×EC_50_, this inhibitory effect on EPS production became even more pronounced, particularly in the NI6R@CB[7]@*β*‐CD treatment group, where the production rate dropped to just 28.2%. These findings suggest that the biocompatible three‐component supramolecular material possesses a unique ability to effectively inhibit EPS, ultimately breaking down biofilm barriers. To further probe the underlying reason, the transcriptional level of the relevant gum gene cluster that can modulate the synthesis and transport of EPS in *Xoo*,^[^ ^38^ ^]^ was detected by qRT‐PCR experiments (Table S5, Supporting Information). As illustrated in Figure S28 (Supporting Information), upon the treatment of NI6R@CB[7]@*β*‐CD at 4.44 *µg* mL^−1^, the expression levels of gum genes (*gumB, gumC, gumD, gumE, gumG, gumK, gumM*) were markedly downregulated compared to treatment with NI6R, NI6R@*β*‐CD, and NI6R@CB[7], which disclosed that the biofilm disruption was potentially ascribed to the prominent downregulation of EPS expression by NI6R@CB[7]@*β*‐CD. On the other hand, the motility of bacteria and the secretion of extracellular enzymes play a key role in protecting bacteria from host immune attack, promoting bacterial attachment, and infecting host tissues.^[^ ^39^ ^]^ An in‐depth understanding of these virulence factors affected by NI6R@CB[7]@*β*‐CD is essential in the development of new agrochemicals. It can be observed from Figure 4C,D that the swimming diameters of bacterial colonies in the negative control groups (0.1% DMSO, *β*‐CD, CB[7]) were 26.8, 26.5, and 25.6 mm, respectively. When the active ingredient was set to 2× or 4×EC_50_, the swimming diameters of NI6R@CB[7]@*β*‐CD were reduced to 14.7 and 12.0 mm, which were much better than the other components. This interesting action of inhibiting bacterial swimming may further hinder the diffusion and migration of pathogens in plant tissues. Next, the extracellular enzyme secretion triggered by NI6R@CB[7]@*β*‐CD was carried out and provided in Figure 4E–G. Notably, NI6R@CB[7]@*β*‐CD displays the most significant inhibitory effect on the secretion of *Xoo* extracellular enzymes, and the hydrolysis circles of its extracellular cellulase and amylase at 4×EC_50_ were only 13.0 and 14.2 mm, respectively, which were smaller than that of NI6R itself (16.0 and 17.2 mm). This function will potentially prevent bacteria from invading by degrading the host cell wall. Given these promising functions, the actual pathogenicity experiment on rice plants was performed. As shown in Figure 4H and Figure S29 (Supporting Information), a significant reduction in the length of infected lesions were observed in the NI6R@*β*‐CD, NI6R@CB[7], and NI6R@CB[7]@*β*‐CD treatment groups, with lesion lengths of 13.53, 12.47, and 6.51 cm, respectively. Notably, the NI6R@CB[7]@*β*‐CD treatment group emerged the most pronounced effect, which correlates with its superior inhibitory potency to various virulence factors. In summary, the fabricated three‐component supramolecular material (NI6R@CB[7]@*β*‐CD) can violently inhibit the virulence factors (biofilm formation, bacterial motility, extracellular enzymes secretion) and pathogenicity of *Xoo* strains, thus weakening the severity of rice bacterial blight disease.

### 2.6 NI6R@CB[7]@*β*‐CD Droplets have Excellent Retention and Deposition Properties on Hydrophobic Rice Leaves

**Figure 5:**
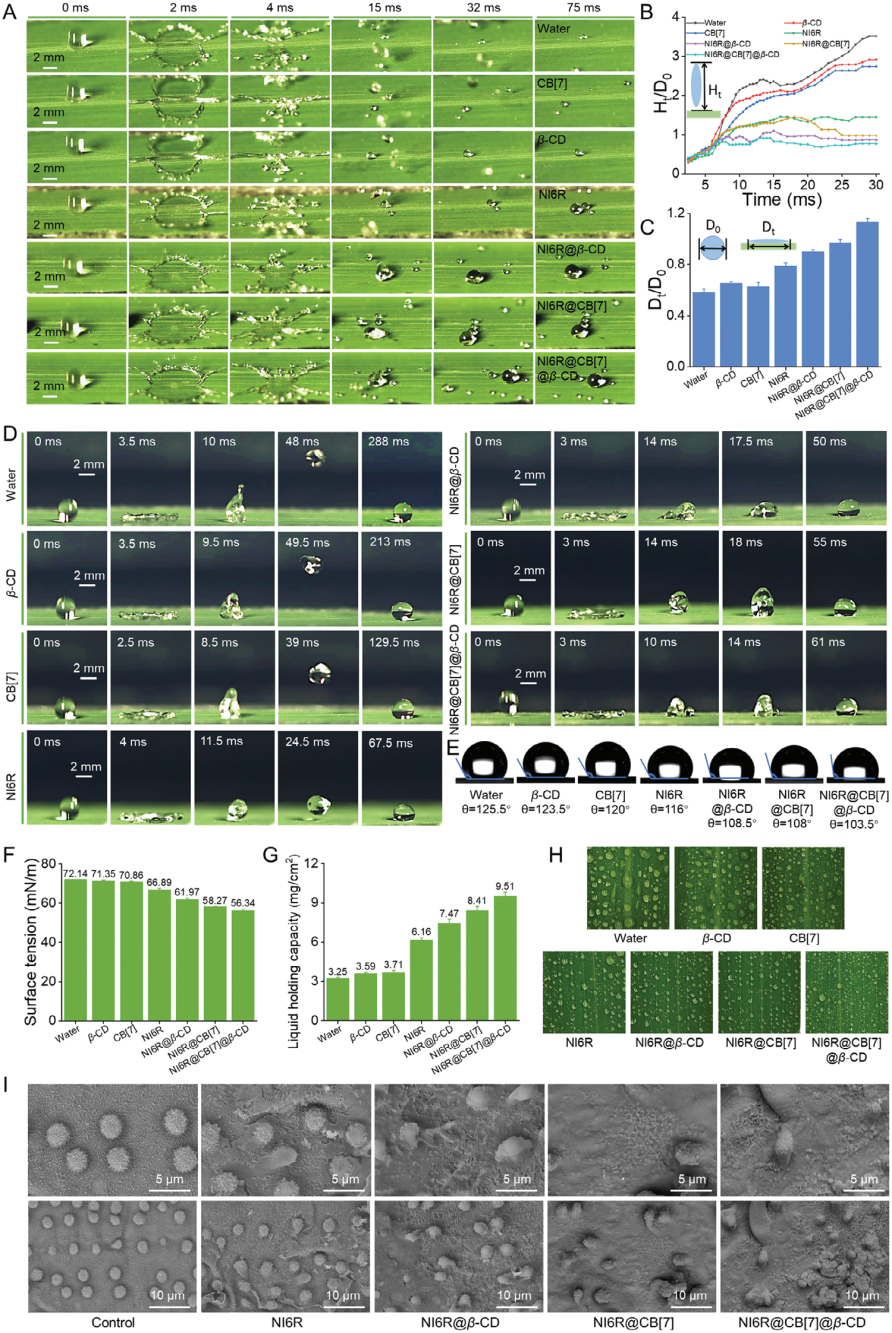
A) High‐speed camera footage of water, *β*‐CD, CB[7], NI6R, NI6R@*β*‐CD, NI6R@CB[7], and NI6R@CB[7]@*β*‐CD droplets splashing from a height of 40 cm on rice leaves. B) Diagram showing the normalized rebound height (H_t_/D_0_) of each component, where D_0_ and H_t_ represent the initial droplet diameter and rebound height during shrinkage. C) Diagram of the normalized diffusion diameter (D_t_/D_0_) of each droplet in its final stage, with D_t_ representing the spreading diameter when landing on rice leaves. D) Bouncing behavior of water, *β*‐CD, CB[7], NI6R, NI6R@*β*‐CD, NI6R@CB[7], and NI6R@CB[7]@*β*‐CD droplets from a 10 cm height onto rice leaves. E) Static contact angle of droplets on rice leaves. F) Surface tension measurements of water, *β*‐CD, CB[7], NI6R, NI6R@*β*‐CD, NI6R@CB[7], and NI6R@CB[7]@*β*‐CD at 200 *µg* mL^−1^. G) Liquid‐holding capacity of NI6R, NI6R@*β*‐CD, NI6R@CB[7], and NI6R@CB[7]@*β*‐CD on rice leaves at 200 *µg* mL^−1^. H) Images of various droplets sprayed on rice leaves. I) SEM images of different samples deposited on rice leaves at 200 *µg* mL^−1^.

In the process of pesticide spraying, a large number of droplets will rebound, rupture, and splash after hitting the surface of hydrophobic leaves, resulting in a small fraction of active ingredients eventually staying on the surface of leaves, which cannot achieve the ideal deposition effect and pesticide utilization rate. Therefore, this section evaluates the retention and deposition characteristics of NI6R@CB[7]@*β*‐CD on rice leaves. Droplets of various components were uniformly released from a height of 40 cm, and the entire process was captured using a high‐speed video camera (Video S1, Supporting Information, **Figure** **5**A). During this process, the spreading area of the droplets of NI6R@CB[7]@*β*‐CD, NI6R@*β*‐CD or NI6R@CB[7] reaches its maximum within 2–4 ms, which is larger than those of other controls (0.4% DMSO, *β*‐CD, CB[7]). In the final state of splashing (75 ms), it is also clearly observed that the retention volume of NI6R@CB[7]@*β*‐CD, NI6R@*β*‐CD or NI6R@CB[7] droplets is also significantly excess to NI6R itself and other components, suggesting that the supramolecular complex has good retention properties. To further explore the bouncing behavior, the droplets were released from a uniform height of 10 cm (Video S2, Supporting Information, Figure 5D). During the analysis, the normalized bounce height H_t_/D_0_ (Figure 5B) and the normalized diffusion diameter D_t_/D_0_ (Figure 5C) were statistically evaluated. Notably, droplets of NI6R@CB[7]@*β*‐CD, NI6R@*β*‐CD, and NI6R@CB[7] displayed no significant rebound, with H_t_/D_0_ values of 0.77, 0.86, and 0.97, respectively. These were considerably lower than the values for NI6R alone (1.44), CB[7] (2.74), *β*‐CD (2.92), and H_2_O (3.51). Additionally, the control droplets had small D_t_/D_0_ values (0.58–0.63), indicating poor spreading on the hydrophobic leaf surface. In contrast, droplets of NI6R@CB[7]@*β*‐CD, NI6R@*β*‐CD, and NI6R@CB[7] had larger D_t_/D_0_ values (0.9–1.13), suggesting better wetting and spreading on the leaf surface. To compare the influence of chiral configuration on droplet retention and deposition behavior, the relevant supramolecular complexes NI6S@*β*‐CD, NI6S@CB[7], and NI6S@CB[7]@*β*‐CD were constructed based on NI6S (*S*‐configuration) to test their bounce and splash levels on rice leaves. As a consequence, these inclusions in aqueous solutions had similar effectiveness, and also effectively inhibited the bounce and splash of droplets, suggesting that the configuration of small molecules had no influence (Figures S30 and S31, Supporting Information).

To explore the possible reasons for their superior deposition ability, the correlative contact angle and surface tension tests for each droplet were performed (Figure 5E,F). Clearly, after loading NI6R with CB[7]@*β*‐CD, *β*‐CD, or CB[7] to form supramolecular inclusions (NI6R@CB[7]@*β*‐CD, NI6R@*β*‐CD, and NI6R@CB[7]), their contact angles were reduced to 103.5°, 108.5°, and 108.0°, respectively, which were lower than those of NI6R and other controls (116°–125.5°). A lower contact angle for NI6R@CB[7]@*β*‐CD droplets implies that they can form a larger contact area on the leaf surface. A similar trend was observed in the surface tension test, providing the corresponding value of 56.34, 61.97, and 58.27 mN m^−1^ for NI6R@CB[7]@*β*‐CD, NI6R@*β*‐CD and NI6R@CB[7], which were also smaller to those of NI6R and other components (66.89–72.14 mN m^−1^). The combination of these characteristics enables these supramolecular assemblies to be more effectively deposited on the leaf surface during spraying, thereby maximizing the utilization of active ingredients. The following liquid holding capacity (LHC) measurement further illuminates the retention of droplets on the hydrophobic leaves (Figure 5G), in which NI6R@CB[7]@*β*‐CD gives the maximal retention capacity of 9.51 mg cm^−2^. This effect is obviously superior to other components (e.g., NI6R, 6.16 mg cm^−2^; H_2_O, 3.25 mg cm^−2^), consistent with the above inference.

Subsequent macroscopic and microscopic blade surface spray observation and SEM analysis further confirmed its superior deposition performance. As shown in Figure 5H, compared to droplets of other components, NI6R@CB[7]@*β*‐CD droplets on hydrophobic rice leaves were smaller, denser, and more uniform in size—highly favorable for agrochemical applications. Microscopic observations revealed that while NI6R formed a relatively thin film on the blank rice leaf surface, NI6R@CB[7]@*β*‐CD, NI6R@*β*‐CD, and NI6R@CB[7] exhibited more uniform and thicker coverage. These results clearly demonstrate the superior deposition properties of these assemblies on rice leaves (Figure 5I). In conclusion, the above tests disclose that the construction of supramolecular assemblies prominently enhances the deposition efficiency of active substrates and has good potential for application.

### 2.7 NI6R@CB[7]@*β*‐CD Achieves Efficient Control of Rice Bacterial Blight In Vivo

**Figure 6:**
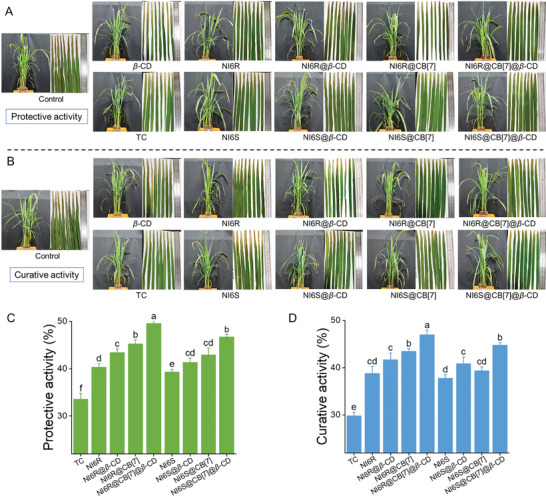
A,C) Photographs and in vivo protective effects of NI6R, NI6R@*β*‐CD, NI6R@CB[7], NI6R@CB[7]@*β*‐CD, NI6S, NI6S@*β*‐CD, NI6S@CB[7], NI6S@CB[7]@*β*‐CD, and the commercial bactericide TC‐20%SC against rice bacterial leaf blight at an effective dose of 200 *µg* mL^−1^ (with cultures maintained for an additional 14 days post‐spraying). B,D) Photographs and in vivo curative effects of the same treatments against rice bacterial leaf blight at 200 *µg* mL^−1^ (agents were sprayed after inoculation and cultured for 14 more days). A single‐factor ANOVA test was applied, with lowercase letters denoting statistically significant differences among the components (*p* < 0.05, Waller–Duncan method).

Building on the excellent biofilm eradication, sterilization, and leaf deposition performance of NI6R@CB[7]@*β*‐CD, its in vivo bactericidal efficacy against *Xoo* was further assessed using the leaf‐cutting method at 200 *µg* mL^−1^. Meanwhile, the *S*‐type supramolecular complex (NI6S@CB[7]@*β*‐CD) was also tested to explore the influence of absolute configuration on in vivo activity. As shown in **Figure** **6**A–D and Table S6 (Supporting Information), the negative control group showed more severe leaf‐blight disease patterns, whereas rice in the NI6R@*β*‐CD, NI6R@CB[7] and NI6R@CB[7]@*β*‐CD treatment groups exhibited varying degrees of disease reduction. Particularly, NI6R@CB[7]@*β*‐CD gave the best protective and curative activities of 49.6% and 47.0%, respectively, which were notably better than the commercial bactericide TC‐20%SC (33.6%/29.8%) and NI6R itself (40.3%/38.8%). For the *S*‐type supramolecular complex (NI6S@CB[7]@*β*‐CD), it also exhibited better control efficiency, which were 46.7% (protective effect) and 44.8% (curative effect), respectively, but both were weaker than the *R*‐type supramolecular complex (NI6R@CB[7]@*β*‐CD). Overall, in terms of in vivo bioactivity, the three‐component supramolecular material (NI6R@CB[7]@*β*‐CD) demonstrates potential application value. These results not only confirmed that we successfully designed a highly active molecule NI6R, but also optimized this molecule through the host‐guest supramolecular strategy, and finally achieved synergistic control of rice bacterial diseases.

### 2.8 NI6R@CB[7]@*β*‐CD Exhibits Broad‐Spectrum In Vitro and In Vivo Bioactivity Against Citrus/Kiwifruit Bacterial Canker

**Figure 7:**
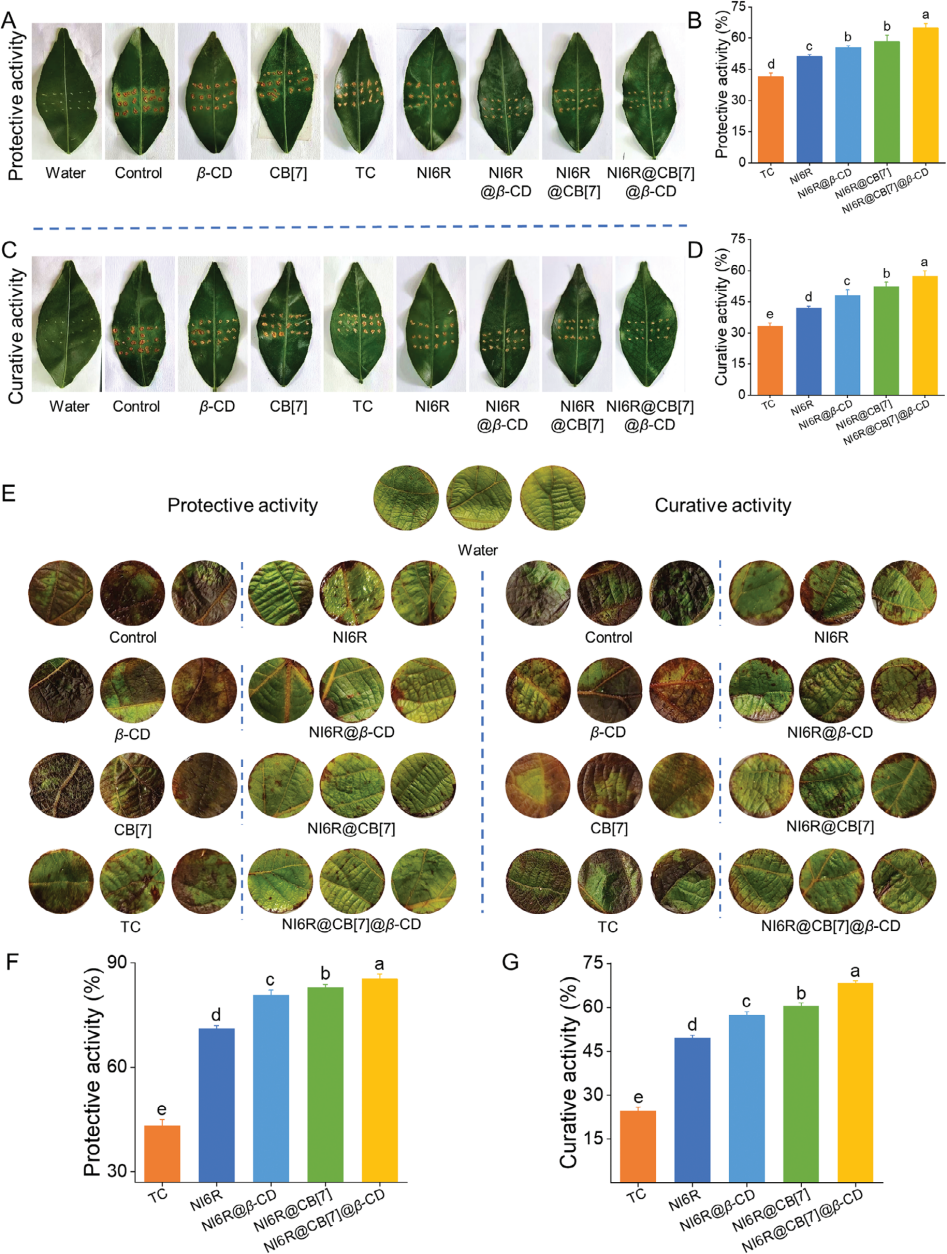
A,B) Photographs and in vivo protective effects of NI6R, NI6R@*β*‐CD, NI6R@CB[7], NI6R@CB[7]@*β*‐CD, and TC‐20%SC against citrus bacterial canker at 200 *µg* mL^−1^ (cultured for an additional 14 days post‐spraying). C,D) Photographs and in vivo curative effects of NI6R, NI6R@*β*‐CD, NI6R@CB[7], NI6R@CB[7]@*β*‐CD, and TC‐20%SC at 200 *µg* mL^−1^ (applied after inoculation and cultured for 14 days). E–G) Photographs and in vivo control effects of NI6R, NI6R@*β*‐CD, NI6R@CB[7], NI6R@CB[7]@*β*‐CD, and TC‐20%SC at 200 *µg* mL^−1^ (cultured for five additional days post‐spraying). A single‐factor ANOVA test was applied, with lowercase letters denoting statistically significant differences between the components (*p* < 0.05, Waller–Duncan method).

To verify the universality of the current system for other bacterial diseases, the in vitro bioactivity against high‐risk *Xanthomonas axonopodis* pv. *citri* (*Xac*) and *Pseudomonas syringae* pv. *actinidiae* (*Psa*) were assessed.^[^ ^40^ ^]^ As presented in Table S7 (Supporting Information), NI6R showed strong anti‐*Xac* and anti‐*Psa* activities with EC_50_ values of 1.05 and 1.16 *µg* mL^−1^, respectively, which were lower than those of the *S*‐type small molecule NI6S (1.88 and 1.87 *µg* mL^−1^) and much lower than the commercial bactericides (TC and BT, EC_50_ = 91.1–144 *µg* mL^−1^). Based on these in vitro results, the in vivo efficacy of NI6R@CB[7]@*β*‐CD against citrus and kiwifruit bacterial canker was further evaluated, along with other groups for comparison (e.g., NI6R, NI6R@*β*‐CD, NI6R@CB[7], and *S*‐configuration forms such as NI6S, NI6S@*β*‐CD, NI6S@CB[7], NI6S@CB[7]@*β*‐CD). As shown in **Figures** **7**A–D and S32 (Supporting Information), the *Xac* infection in the control group was markedly more severe, with more yellow necrotic spots observed. In contrast, citrus leaves treated with NI6R@*β*‐CD, NI6R@CB[7], and NI6R@CB[7]@*β*‐CD exhibited notably milder symptoms, with protective activities of 55.6%, 58.3%, and 65.0%, respectively, and curative activities of 48.0%, 52.3%, and 57.4%. These results indicate a substantial improvement over NI6R alone (51.2%/41.9%) and also markedly surpass the commercial bactericide TC‐20%SC (41.5%/33.2%). Besides, both the protective and curative activities of these supramolecular complexes were superior to those prepared with the *S*‐enantiomer, including NI6S@*β*‐CD, NI6S@CB[7], and NI6S@CB[7]@*β*‐CD (Figure S32, Supporting Information).

Subsequently, the in vivo bactericidal activity to *Psa* was assessed and illustrated in Figure 7E–G. Clearly, the leaves of the negative control group showed large brown susceptible areas, whereas the kiwifruit leaves treated with NI6R@*β*‐CD, NI6R@CB[7] and NI6R@CB[7]@*β*‐CD showed brighter green color and less susceptible areas, with a protective activity of 80.8%, 83.0%, and 85.4%, respectively, and the curative activity was 57.3%, 60.5% and 68.3%, respectively (Figure 7F,G), which was significantly better than that of the commercial bactericide TC‐20%SC (protective activity: 43.2%; curative activity: 24.6%) and NI6R itself (protective activity: 71.2%; curative activity: 49.5%). Meanwhile, the supramolecular inclusions with *R*‐configuration were found to be superior to those complexes constructed with *S*‐configuration both in protective and curative activities (Figure S33, Supporting Information).

The above outcomes indicate that the three‐component supramolecular material (NI6R@CB[7]@*β*‐CD) prepared by this study has good control efficiency against many intractable plant bacterial diseases, which provides new solution ideas for agricultural disease control.

### 2.9 NI6R@CB[7]@*β*‐CD Shows Good Biosafety to Rice Plants, Paddy, Zebrafish, and Earthworm

**Figure 8:**
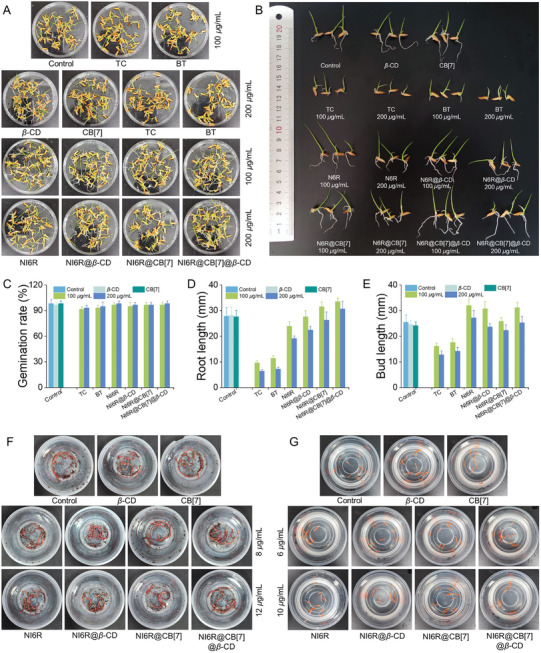
A, B) Safety assessment of seed germination and seedlings following treatment with NI6R, NI6R@*β*‐CD, NI6R@CB[7], and NI6R@CB[7]@*β*‐CD at 100 and 200 *µg* mL^−1^. C–E) Evaluated seed germination rates, root length, and shoot length. F) Acute toxicity testing of NI6R, NI6R@*β*‐CD, NI6R@CB[7], and NI6R@CB[7]@*β*‐CD on earthworms at concentrations of 8 and 12* µg* mL^−1^. G) Acute toxicity testing of NI6R, NI6R@*β*‐CD, NI6R@CB[7], and NI6R@CB[7]@*β*‐CD on zebrafish at 6 and 10 *µg* mL^−1^.

The biosafety evaluation is an important index for creating new agrochemicals, thus, the toxicity analysis of each component on model organisms, such as rice itself, zebrafish, and earthworm, was carried out. The effects of different samples on rice seed germination were first investigated, and the seed germination rates for the control group and each experimental group at 100 and 200 *µg* mL^−1^ were greater than 93.3% without significant differences (**Figure** **8**A,C). Next, the effects of different samples on the root length of rice seedlings were investigated. When the concentration of the active ingredient was 100 *µg* mL^−1^, the growth of seed roots in the TC and BT treatment groups was significantly inhibited with only 9.7–11.4 mm compared with the 28 mm root length of the control group. It is noteworthy that NI6R@CB[7] and NI6R@CB[7]@*β*‐CD promoted root growth to a certain extent up to 31.5–33.5 mm (Figure 8B,D), which may be attributed to the growth‐promoting effect of nitrogen‐rich cucurbit[7]uril molecules in the two supramolecular inclusions. However, this effect has not been achieved on *β*‐CD‐involved supramolecular inclusion (NI6R@*β*‐CD). When the concentration of the active ingredient was increased to 200 *µg* mL^−1^, the root growth of all component treatments, although inhibited to some extent, was still the highest in the NI6R@CB[7]@*β*‐CD treatment group, which reached 30.7 mm (blue columns). For the rice shoot growth, similar to the trend of root length, the TC and BT treatment groups also showed some inhibitory effects on rice shoots, whereas 100 *µg* mL^−1^ of NI6R@CB[7]@*β*‐CD still had a certain effect on promoting shoot growth (Figure 8B,E). In addition, since quaternary ammonium salts may have potential phytotoxicity, therefore, the corresponding tests were conducted on rice plants (Figure S34, Supporting Information). At concentrations of 200 and 500 *µg* mL^−1^, among various supramolecular agents, the rice leaves treated with NI6R@CB[7] and NI6R@CB[7]@*β*‐CD remain healthy and have no negative effect. However, at 200 *µg* mL^−1^, the NI6R treatment group showed sporadic black spots, and the other treatment groups showed no abnormal problems. When the concentration reached 500 *µg* mL^−1^, the rice leaves of the NI6R treatment group gave more black spots, indicating more serious damage. This finding indicates that the use of host‐guest supramolecular optimization strategies can effectively improve the biosafety of imidazolium salts in practical applications, which is consistent with the literature.^[^ ^41^ ^]^ The possible underlying reason for this outcome is attributed to the charge‐transfer capability of CB[7], which reduces the free diffusion and excessive accumulation of quaternary ammonium salts in plant cells. Consequently, it minimizes the direct interaction of the active ingredients with plant cells, thereby reducing its phytotoxicity.

Subsequently, acute toxicity experiments were conducted to evaluate the safety of each component on non‐target organisms such as earthworm and zebrafish (Figure 8F,G). It was observed that at concentrations of 8 and 12 *µg* mL^−1^, the survival rate of earthworm in the NI6R@CB[7]@*β*‐CD and other treatment groups was 100% (Figure S35A, Supporting Information). At a concentration of 10 *µg* mL^−1^, the survival rate of zebrafish exceeded 93.3% in the NI6R‐only treatment group, while in all other groups, including NI6R@CB[7]@*β*‐CD, the zebrafish survival rate was 100% (Figure S35B, Supporting Information). According to the acute toxicity evaluation standard (LC_50_>10 *µg* mL^−1^, indicating low toxicity),^[^ ^42^ ^]^ all the tested supramolecular bactericides demonstrated low toxicity toward both earthworm and zebrafish. This suggests that these supramolecular inclusions possess good biosafety and biocompatibility, which is significant for the development and adoption of environmentally friendly green pesticides.

## 3 Conclusion

This study presents a transformative approach to addressing the persistent challenges of biofilm resistance and poor droplet deposition in agricultural pathogen control. The fabricated supramolecular complex NI6R@CB[7]@*β*‐CD demonstrated exceptional efficacy in disrupting mature biofilms, inhibiting bacterial reproduction and motility, and reducing pathogenicity, while effectively improving pesticide deposition on hydrophobic rice leaves. Moreover, NI6R@CB[7]@*β*‐CD has broad‐spectrum in vitro and in vivo bactericidal activity against major plant pathogens, which markedly surpasses conventional treatments. Importantly, its low toxicity and biosafety against crops and non‐target organisms highlight its potential as a next‐generation, environmentally sustainable solution for agricultural disease management. This work exemplifies the power of supramolecular chemistry in revolutionizing agrochemical development, paving the way for more effective, biologically safe plant protection strategies.

## 4 Experimental Section

### In Vitro Antibacterial Bioassay

The virulence regression equations and EC_50_ values of all the target compounds and positive control bactericides (bismerthiazol and thiodiazole‐copper) against *Xanthomonas oryzae* pv. *oryzae* (*Xoo*), *Xanthomonas axonopodis* pv. *citri* (*Xac*) and *Pseudomonas syringae* pv. *actinidiae* (*Psa*) were determined by turbidimetric method. 5.0 mL of active ingredients‐containing NB medium (NB composition: glucose 10 g, peptone 5.0 g, beef paste 3.0 g, yeast powder 1.0 g, distilled water 1.0 L, pH 7.2) was prepared on a sterile ultra‐clean bench at the corresponding concentrations of target compounds and commercial bactericides, then adding 40 *µL* of the bacterial solution to the above system, and finally incubate in a constant temperature shaker (28 °C, 220 rpm) with shaking for 24–48 h until the turbidity of the blank control (0.4% DMSO) reached OD_595_ _nm_ = 0.6. Subsequently, the corresponding antibacterial activity calculations were performed. Pipette 200 *µL* of the samples onto a 96‐well plate and determine the OD_595_ _nm_ values of each concentration on an enzyme labeling instrument, setting three parallels for each sample. Turbidity corrected values were OD_595 with bacteria_ – OD_595 without bacteria_. The inhibition rate (I) can be obtained from I (%) = (C‐T)/C × 100 (C and T are the turbidity‐corrected values of the negative control and the actual sample, respectively). Based on the inhibition rate at different concentrations, the toxicity regression equation and the half inhibitory concentration value (EC_50_) were calculated using Excel software.^[^ ^31^ ^]^

### Preparation of Supramolecular Complexes

For comparison, two binary supramolecular complexes (NI6R@CB[7] and NI6R@*β*‐CD) were also prepared. Briefly, NI6R (4.0 *µL*, 64.1 mm) dissolved in dimethyl sulfoxide (DMSO) was dropped into 0.992 mL deionized aqueous solution containing CB[7] (0.256 mm). After ultrasonic oscillation for 15 mins at room temperature, the turbid system became a transparent solution, indicating the formation of binary supramolecular complex (NI6R@CB[7]). Later, *β*‐CD (4.0* µL*, 64.1 mm) dissolved in deionized water was supplemented into the above solution. After fully mixing for 5 mins at room temperature, a three‐component supramolecular material (NI6R@CB[7]@*β*‐CD, molar ratio, 1:1:1) was fabricated, in which the effective concentration of NI6R is 0.256 mm (200 *µg* mL^−1^) and the fraction of DMSO is 0.4% (V/V).

### Biofilm Inhibition Experiment

First, the *Xoo* bacterial solution with resuspended overnight (OD_595_ _nm_ = 0.6) was adjusted to OD_595_ _nm_ = 0.1 with sterilized NB medium. 200 *µL* of the bacterial solution was added into 96‐well plates, and NI6R, NI6R@*β*‐CD, NI6R@CB[7], and NI6R@CB[7]@*β*‐CD were added by the half‐fold dilution method, so that the concentrations in the bacterial solution were 0.5×, 1×, 2×, 4×, and 8×EC_50_, respectively. Next, the OD_595_ _nm_ values were determined after 48 h of incubation in an incubator at 28 °C. After the incubation was completed, the *Xoo* suspension cells were gently removed, washed 3 times with sterile water, and dried at room temperature for 4 h. To each well, 200 *µL* of 0.1% crystal violet solution (*ω*/v) was added, and the staining was allowed to stand for 15 mins, then the crystal violet solution was removed, and the excess of crystal violet solution was rinsed three times with sterile water. Then it was dried in an oven at 40 °C for 1 h. 200 *µL* of 95% ethanol solution was added to completely dissolve the crystal violet. Finally, the OD_570_ _nm_ value was measured to determine the biofilm content.^[^ ^43^ ^]^

### Biofilm Eradication Experiment

First, the *Xoo* bacterial solution resuspended overnight with OD_595_ _nm_ = 0.6 was adjusted to OD_595_ _nm_ = 0.1 with sterilized NB medium. 200 *µL* of bacterial solution was added to a 96‐well plate and incubated in an incubator at 28 °C for 36 or 48 h. Then, the medium was removed, and the planktonic bacteria were washed twice with PBS buffer (pH 7.4), and then added to the plate with concentrations of 1×, 2×, 4×, 8×, 16×, 32×, 64×, 128×EC_50_ NI6R, NI6R@*β*‐CD, NI6R@CB[7], and NI6R@CB[7]@*β*‐CD, respectively. The incubation was continued in an incubator at 28 °C for 24 h and the OD_595_ _nm_ values were determined. Finally, the removal of mature biofilm by the agents was determined using the same procedure as the biofilm inhibition crystal violet staining method.^[^ ^35^ ^]^

### Rice Droplet Splashing and Bouncing Experiments

The dynamic impact of droplets on rice leaves was captured with an i‐SPEED 220 high‐speed camera at 2000 fps. Water and 200 *µg* mL^−1^ of *β*‐CD, CB[7], NI6R, NI6R@*β*‐CD, NI6R@CB[7], NI6R@CB[7]@*β*‐CD, etc. were injected drop‐by‐drop with a microwell syringe with an inner diameter of 0.25 mm. The videos were processed and analyzed using i‐SPEED Suite software. The heights of the droplet splash and bounce experiments were 40 and 10 cm, respectively.^[^ ^18^ ^]^

## Conflict of Interest

The authors declare no conflict of interest.

## Supporting information



Supporting Information

Supplemental Video 1

Supplemental Video 2

## Data Availability

The data that support the findings of this study are available in the supplementary material of this article.
