# Supplementary material for: Fabrication of Multifunctional Three‐Component Supramolecular Nano‐Biscuits via Two Macrocycles‐Involved Self‐Assembly for Rice, Citrus and Kiwifruit Protections
Source: Adv Sci (Weinh). 2025 Jan 24;12(11):2413826. doi: 10.1002/advs.202413826 (PMC11923968; doi:10.1002/advs.202413826)
Supplement: Supplementary file 1 — Supporting Information [file ADVS-12-2413826-s002.docx]

Supporting Information

**Fabrication of Multifunctional Three-Component Supramolecular Nano-Biscuits via Two Macrocycles-Involved Self-Assembly for Rice, Citrus and Kiwifruit Protections**

*Xinyu He, ^†^ Jinghan Yang,^†^ Xue Chen, Jiajia Chen, Haicong Zhao, Fengpei Du, Juan Liu, and Peiyi Wang****

X.-Y. He, J.-H. Yang, X. Chen, J.-J. Chen, H.-C. Zhao, J. Liu, Prof. P.-Y. Wang

State Key Laboratory of Green Pesticide, Key Laboratory of Green Pesticide and Agricultural Bioengineering, Ministry of Education, Center for Research and Development of Fine Chemicals of Guizhou University, Guiyang, 550025, China.

Prof. F.-P. Du

Department of Applied Chemistry, College of Science, China Agricultural University, Beijing, 100193, China.

^*^Corresponding author E-mail: pywang888@126.com; pywang@gzu.edu.cn (P.-Y. Wang)

^†^ The two authors contribute equally to this work.

Contents

[1. Experimental Section 1](#_Toc187053182)

[1.1 Chemicals and Instruments 1](#_Toc187053183)

[1.2 UV-Vis Titration Experimenting 2](#_Toc187053184)

[1.3 Job's Plot Experiment 2](#_Toc187053185)

[1.4 Zeta Potential Determination 3](#_Toc187053186)

[1.5 Confocal Laser Scanning Microscope (CLSM) 3D Imaging 3](#_Toc187053187)

[1.6 SEM of Bacterial Biofilm 3](#_Toc187053188)

[1.7 Antibacterial Activity Analysis (Coated Plate Method) 4](#_Toc187053189)

[1.8 Swimming Motility Assay 4](#_Toc187053190)

[1.9 Determination of Bacterial Extracellular Polysaccharides (EPS) 5](#_Toc187053191)

[1.10 Determination of Extracellular Cellulase 5](#_Toc187053192)

[1.11 Determination of Extracellular Amylase 6](#_Toc187053193)

[1.12 Rice Pathogenicity Assay 6](#_Toc187053194)

[1.13 SEM of Deposition on Rice Leaf 6](#_Toc187053195)

[1.14 Liquid Holding Capacities Assay 7](#_Toc187053196)

[1.15 *In Vivo* Antibacterial Bioassay against Rice Bacterial Leaf Blight 7](#_Toc187053197)

[1.16 Rice Phytotoxicity Assay 8](#_Toc187053198)

[1.17 *In Vivo* Antibacterial Bioassays against Citrus Canker 8](#_Toc187053199)

[1.18 *In Vivo* Antibacterial Bioassays against Kiwifruit Canker 9](#_Toc187053200)

[1.19 Germination Experiment of Rice Seed 10](#_Toc187053201)

[1.20 Earthworm Safety Experiment 10](#_Toc187053202)

[1.21 Zebrafish Safety Experiment 11](#_Toc187053203)

[1.22 Statistical Data Processing 11](#_Toc187053204)

[2. Synthesis Procedures and Characterization Data for Target Compounds 11](#_Toc187053205)

[3. ^1^H NMR, ^13^C NMR, HPLC, and HRMS Spectra of Target Compounds 15](#_Toc187053206)

[4. Supplementary Figures and Tables 19](#_Toc187053207)

[4.1 Characterization of NI6R@CB[7] 19](#_Toc187053208)

[4.2 Characterization of NI6R@*β*-CD 20](#_Toc187053209)

[4.3 HRMS Spectrum of Supramolecular Complex NI6R@CB[7]@*β*-CD 22](#_Toc187053210)

[4.4 ^1^H-^1^H ROESY Spectra of NI6R, NI6R@CB[7] and NI6R@CB[7]@*β*-CD 22](#_Toc187053211)

[4.5 2D Diffusion Ordered Spectroscopy (DOSY) of NI6R, NI6R@CB[7] and NI6R@CB[7]@*β*-CD. 24](#_Toc187053212)

[4.6 UV-vis Spectra of Different Concentrations of NI6R in Water and Schematic Representation of the Lowest Energy in Chem 3D 26](#_Toc187053213)

[4.7 Assembly Mechanisms of Supramolecular Building Blocks NI6R and NI6R@CB[7] 26](#_Toc187053214)

[4.8 Biofilm Inhibition Test 27](#_Toc187053215)

[4.9 Biofilm Formation at Different Periods 28](#_Toc187053216)

[4.10 Crystal Violet Staining for Biofilm Eradication 28](#_Toc187053217)

[4.11 Survival Rate of *Xoo* Bacteria Enclosed by Biofilm 29](#_Toc187053218)

[4.12 OD_490 nm_ Value of Exopolysaccharides Solution 30](#_Toc187053219)

[4.13 The Transcriptional Level of the Interrelated *Gum* Gene Cluster that Can Regulate the Synthesis and Transport of EPS in *Xoo* 30](#_Toc187053220)

[4.14 Length of Leaf Lesion in Rice Pathogenicity 31](#_Toc187053221)

[4.15 Investigation of Droplet Splashing on Rice Leaves 32](#_Toc187053222)

[4.16 Investigation of Droplet Bouncing on Rice Leaves 32](#_Toc187053223)

[4.17 *In Vivo* Control of NI6S@CB[7]@*β*-CD on *Xac* 33](#_Toc187053224)

[4.18 *In Vivo* Control of NI6S@CB[7]@*β*-CD on *Psa* 33](#_Toc187053225)

[4.19 Phytotoxicity Studies of NI6R, NI6R@*β*-CD, NI6R@CB[7] and NI6R@CB[7]@*β*-CD on Rice Plants 34](#_Toc187053226)

[4.20 Acute Toxicity of NI6R, NI6R@*β*-CD, NI6R@CB[7] and NI6R@CB[7]@*β*-CD to Earthworm and Zebrafish 34](#_Toc187053227)

[5. Supplementary Tables 35](#_Toc187053228)

[5.1 NI6R/S against Plant Pathogens *Xoo* 35](#_Toc187053229)

[5.2 Chemical Shift Change of Supramolecular Complex NI6R@CB[7] and NI6R@CB[7]@*β*-CD After Assembly 35](#_Toc187053230)

[5.3 Efficiency of *in Vivo* Control of Rice Bacterial Leaf Blight 37](#_Toc187053231)

[5.4 NI6R/S against Plant Pathogens *Xac* and *Psa* 37](#_Toc187053232)

[References 38](#_Toc187053233)

# 1. Experimental Section

## 1.1 Chemicals and Instruments

The chemical reagents used, *β*-naphthol (98%), (*R*)-epichlorohydrin (99% purity), (*S*)-epichlorohydrin (99%) (Sarn Chemical Technology (Shanghai) Co., Shanghai, China); imidazole (99%), *β*-cyclodextrin (99%) (Anhui Zesheng Technology Co., Ltd, Anqing, Anhui, China); and CB[7] (98%) (Nanjing Parkway Pharmaceutical Technology Co., Ltd, Nanjing, Jiangsu, China) were purchased from commercial sources. The hydrogen spectrum and carbon spectral data were detected by NMR spectrometer (JEOL-ECX-500 (Nippon Electronics Corporation, Musashino, Akishima, Tokyo, Japan) and Bruker Biospin-AG-400 (Bruker Spectroscopy Instruments, Ettlingen, Germany)) using DMSO-*d*_6_ and CDCl_3_ as deuterated solvents and TMS as internal standard solution. Corresponding mass spectral data were scanned and approved by a high-resolution mass spectrometer (uitu3000, Thermo Scientific, Thermo Fisher Scientific (China) Co., Ltd., Shanghai, China). The OD value of *in vitro* antibacterial activity was monitored at 595 nm by using Cytation™ 5 multimode readers (BioTek Instruments, Inc., Vermont, USA). The *in vivo* bacterial inhibition experiment was carried out in an intelligent artificial climate chamber (RXZ-436C, Ningbo Jiangnan Instrument Factory, Ningbo, Zhejiang, China). Ultraviolet-visible (UV-vis) spectra were measured with a UV-1900 spectrophotometer (Shimadzu Corporation, Sakyo-ku, Kyoto, Japan). Contact angle experimenting was performed using a JC-2000D1 instrument (Shanghai Zhongchen Digital Technology Instrument Co., Ltd., Shanghai, China). A highly sensitive DLS analyzer (NanoBrook 90Plus PALS) was used to determine the molecular Zeta potential (Brookhaven Instruments, New York, NY, USA). The droplet bouncing and impact experiments were captured by an i-SPEED 220 high-speed camera (Optronis Germany, Kehl, Germany). Compounds and rice samples were visualized and image processed using a FEI Nova NanoSEM 450 scanning electron microscope (SEM) (FEI USA, Hillsboro, Oregon, USA). Biofilm inhibition and eradication samples were processed by 3D visualization photography using a Nikon A1R Confocal Microscope System (Nikon Instruments Inc., Melville, NY, USA).

## 1.2 UV-Vis Titration Experimenting

UV-visible spectra were obtained at this concentration by adding 45 *μ*L (10 mM) of NI6R to cuvette to prepare a 0.15 mM working solution. An aqueous solution of 10 mM *β*-CD/CB[7] was experimented by gradually adding it at a ratio of 0.2 eq to a cuvette containing 1.0 eq of NI6R. The binding constants of the complexes, *K*_a_, were calculated by the Benesi-Hildebrand equation,

$$\frac{\text{1}}{\text{Δ}\text{A}}\text{=}\frac{\text{1}}{\text{α}\text{×Ka}}\text{×}\frac{\text{1}}{\text{c}}\text{＋}\frac{\text{1}}{\text{α}}$$

where *Δ*A is the difference between the absorbance values before (A_0_) and after (A) supramolecular complexes encapsulation, and c and α are the total concentration and the constant of *β*-CD/CB[7], respectively. Ka was obtained by plotting 1/*Δ*A against 1/c (*β*-CD/CB[7]) and calculating the slope and intercept of the resulting line.^[1]^

## 1.3 Job's Plot Experiment

The binding stoichiometry of *β*-CD/CB[7] to compound NI6R was determined using the Job's plot method with continuous variation of UV-vis spectroscopy. A 3.0 mL mixture of NI6R and *β*-CD/CB[7] was configured with different molar ratios N_NI6R_:N*_β_*_-CD/CB[7]_ = 10:0, 9:1, 8:2...0:10. It was left for 1 day and the UV absorbance was measured after its supramolecular self-assembly complete action, *Δ*A is the difference in UV absorbance before and after titration. Job's curve was obtained by plotting the scatter plot of *Δ*A versus N_NI6R_:N_NI6R+_*_β_*_-CD/CB[7]_.

## 1.4 Zeta Potential Determination

Zeta potentials of 200 *μ*g mL^-1^ NI6R, NI6R@*β*-CD, NI6R@CB[7], and NI6R@CB[7]@*β*-CD were measured using a dynamic light scatter meter (DLS).

## 1.5 Confocal Laser Scanning Microscope (CLSM) 3D Imaging

Biofilm inhibition assay: sterilized conductive glass was placed in 12-well cell culture plates, 5.0 mL of *Xoo* bacterial solution (OD_595 nm_ = 0.1) was added to each well, and NI6R, NI6R@*β*-CD, NI6R@CB[7] and NI6R@CB[7]@*β*-CD were added to bring the concentration to 1×, 4×, and 8×EC_50_, respectively, and placed under incubated at 28 ℃ for 48 h. Then, the bacterial solution was removed and washed three times with PBS buffer to remove the floating cells, and acridine orange (0.1% AO) and pyridinium iodide (0.01% PI) were added to stain the cells under dark conditions for 15 min for CLSM 3D imaging.

Biofilm eradication assay: sterilized conductive glass was placed in a 12-well cell culture plate, and *Xoo* cells (OD_595 nm_ = 0.1) were fully cultured for 48 h. After biofilm maturation, NI6R, NI6R@*β*-CD, NI6R@CB[7], and NI6R@CB[7]@*β*-CD were added to bring the concentration to 2×, 32×, and 128×EC_50_, respectively, and incubation was continued at 28 ˚C for 24 h. Then, bacterial fluids were removed and washed three times with PBS buffer to remove floating cells, and staining was performed by adding acridine orange (0.1% AO) and pyridinium iodide (0.01% PI) for 15 min under dark conditions for CLSM 3D imaging. All samples were subjected to CLSM detection using 488 or 543 nm laser.

## 1.6 SEM of Bacterial Biofilm

First, 5.0 mL of *Xoo* bacterial solution with resuspended overnight OD_595 nm_ = 0.6 was added to 12-well cell culture plates fitted with sterilized conductive glass. After 48 h of incubation in an incubator at 28 ℃, the bacterial solution was removed after the biofilm had matured, and the planktonic bacteria were washed with PBS buffer (pH = 7.4), to which 5.0 mL of medium was added, and then NI6R, NI6R@*β*-CD, NI6R@CB[7], and NI6R@CB[7]@*β*-CD were added to give a concentration of supramolecular materials in the bacterial solution of 2×, 32×, and 128×EC_50_, respectively, and the incubation was continued for 24 h. After incubation, slowly aspirate the bacterial liquid in the plate, slowly add 5.0 mL PBS along the wall and wash once, and then add 5.0 mL 2.5% glutaraldehyde to fix it for 12 h. After fixation, aspirate the glutaraldehyde, and then use 5.0 mL ethanol (50%, 70%, 90%, and 100%) solution for dehydrating and replacing, each time for 5 min, and then suck out the ethanol solution and freeze-dry it for 3 h, and then wait for photographing.

## 1.7 Antibacterial Activity Analysis (Coated Plate Method)

The *Xoo* suspension (200 *μ*L, OD_595 nm_ = 0.1) was incubated in a 96-well plate for 48 h. Then NI6R, NI6R@*β*-CD, NI6R@CB[7], and NI6R@CB[7]@*β*-CD at different doses (2×EC_50_, 32×EC_50_, and 128×EC_50_) were added and continued to be incubated for 24 h. Supernatant planktonic bacteria and bacteria encapsulated in the biofilm-coated bacteria were diluted 1.0×10^4^ times with PBS buffer, 10 *μ*L was taken in the middle of the agar plate, spread evenly with a blotting rod, and placed at 28 °C for 5 d. Finally, the number of colonies of each fraction was counted.^[2]^

## 1.8 Swimming Motility Assay

NI6R, NI6R@*β*-CD, NI6R@CB[7], and NI6R@CB[7]@*β*-CD at effective concentrations of 1×EC_50_, 2×EC_50_, and 4×EC_50_, respectively, were added to centrifuge tubes containing 2.0 mL of *Xoo* bacteriophage (OD_595 nm_ = 1). The blank control was dimethyl sulfoxide. Then 2.0 *µ*L of *Xoo* bacterial solution was aspirated and added dropwise to NB liquid medium containing 0.5% agar. It was left to incubate at 28 °C for 5 d. Colony diameter was determined by the crosshatch method.

## 1.9 Determination of Bacterial Extracellular Polysaccharides (EPS)

First, the overnight *Xoo* bacterial solution was resuspended and the OD_595 nm_ value was adjusted to 0.1 with sterilized NB medium. Subsequently, 25 mL of OD_595 nm_ = 0.1 bacterial solution was taken in a 50 mL conical flask, to which 0.1% DMSO or NI6R, NI6R@*β*-CD, NI6R@CB[7], and NI6R@CB[7]@*β*-CD were added to make the concentration of the compounds in the bacterial solution to be 0, 0.56, 1.11, 2.22, and 4.44 *µ*g mL^-1^ respectively, which were incubated in a shaker at 28 ℃ for 5 d at 200 rpm.

Then, the cultured bacterial solution was centrifuged at 10000 rpm for 10 min, and after centrifugation, 0.5 mL of the supernatant was taken, and 0.5 mL of 50 g L^-1^ phenol solution (protected from light) and 2.5 mL of 95% concentrated sulfuric acid were added, and then it was left to stand for 5 min, and 200 *μ*L of it was sucked up to determine the value of OD_490 nm_, and 3 replicates were set up for each sample.

## 1.10 Determination of Extracellular Cellulase

First, NB liquid medium containing *Xoo* cells (OD_595 nm_ = 0.1), NI6R, NI6R@*β*-CD, NI6R@CB[7], and NI6R@CB[7]@*β*-CD (4×EC_50_) was prepared, and the blank control was an equal amount of DMSO. Next, a layer of 1.5% agar was spread in 90 mm Petri dishes, and after it was allowed to solidify, four evenly spaced dishes were placed in each Oxford cups. Then 10 mL of NB medium containing 0.25% sodium carboxymethylcellulose and 1.5% agar powder was added, and after it solidified, the Oxford cup was removed. After that, take 2.0 *μ*L of prepared bacterial solution and add it to the wells formed by the Oxford cup, incubate at 28 ℃ for 3 d. Set up three parallel. 3 d later, wash the colonies with sterile water, add 0.1% Congo red solution to stain the colonies for 30 min, pour off the staining solution, wash with sterile water for two times, and then decolorize the colonies with 10 mL of 1.0 mol L^-1^ NaCl solution for 5 min, and finally measure the diameter of the aperture.

## 1.11 Determination of Extracellular Amylase

First, NB liquid medium containing *Xoo* cells (OD_595 nm_ = 0.1), NI6R, NI6R@*β*-CD, NI6R@CB[7], and NI6R@CB[7]@*β*-CD (4×EC_50_) was prepared, and the blank control was an equal amount of DMSO. Next, a layer of 1.5% agar was spread in 90 mm Petri dishes, and after it was allowed to solidify, four evenly spaced dishes were placed in each Oxford cups. Then, 10 mL of NB medium containing 0.1% soluble starch and 1.5% agar powder was added and allowed to solidify, and the Oxford cup was removed. 2.0 *μ*L of the prepared bacterial solution was added to the wells formed by the Oxford cup and incubated at 28 °C for 3 d. After that, the colonies were washed with sterile water and then stained with I_2_/KI (0.08 mol L^-1^ I_2_ (1%, V/V), 3.2 mol L^-1^ KI (8%)) solution for 5 min, and washed with 70% ethanol solution for three times after staining. Finally, the aperture diameter was measured.

## 1.12 Rice Pathogenicity Assay

NI6R, NI6R@*β*-CD, NI6R@CB[7] and NI6R@CB[7]@*β*-CD at a concentration of 4×EC_50_ and an equal amount of DMSO solution were added to *Xoo* bacterial broth with an OD_595 nm_ = 0.1, and incubated for 12 h at 28 °C and 180 rpm. Leaf cuttings were used to immerse the adult rice plants. After 14 d of incubation in the greenhouse, the damaged length of rice leaves was measured and photographed.

## 1.13 SEM of Deposition on Rice Leaf

Rice intercepts were placed on a carrier stage, and 20 *μ*L of 200 *μ*g mL^-1^ of *β*-CD, CB[7], NI6R, NI6R@*β*-CD, NI6R@CB[7], and NI6R@CB[7]@*β*-CD were pipetted dropwise onto rice leaves. After air-drying at room temperature for 4 h, the samples were sprayed with gold and experimented for characterization by scanning electron microscopy.

## 1.14 Liquid Holding Capacities Assay

Round rice leaves with a diameter of 1.0 cm were intercepted and immersed in *β*-CD, CB[7], NI6R, NI6R@*β*-CD, NI6R@CB[7], NI6R@CB[7]@*β*-CD solutions at a concentration of 200 *μ*g mL^-1^, respectively. 30 s later, the leaves were picked up with forceps until there were no droplets and the mass before and after immersion was weighed with an analytical balance (respectively M_1_ and M_2_). Three parallels were set up for each component sample and LHC was calculated as follows:^[3]^

$LHC = (M_{2}-M_{1}) / S$ (S is the area of the leaf blade).

## 1.15 *In Vivo* Antibacterial Bioassay against Rice Bacterial Leaf Blight

Mature rice plants of the same length were inoculated by the leaf-cutting method, with DMSO, *β*-CD, and CB[7] as negative controls, NI6R, NI6R@*β*-CD, NI6R@CB[7], NI6R@CB[7]@*β*-CD as experimental groups, and thiediazole-copper (TC-20%SC) as positive control.

Protective activity experiment: 200 *μ*g mL^-1^ of the relevant compounds were uniformly sprayed on the leaves, and the leaves were cut at the apical 1-2 cm of rice with sterilized scissors inoculated with *Xoo* cells 24 hours later (OD_595_ _nm_ = 0.6).

Curative activity experiment: The apical 1-2 cm of rice leaves were cut with sterilized scissors inoculated with *Xoo* cells (OD_595 nm_ = 0.6), and then 200 *μ*g mL^-1^ of the relevant compounds were uniformly sprayed on the leaves 24 hours later.

Calculation of control efficiency: The rice samples were incubated in an artificial climate chamber at 28 °C and 90% humidity for 14 days, and the corresponding control effects were obtained by grading standardized calculation methods. The susceptible length of each leaf and the total leaf length were measured, and then the percentage of the susceptible length to the total leaf length was calculated. After that, the susceptible leaves were categorized according to the grading criteria. The disease index (C or T) is calculated as follows:

$$\text{Disease index}\left( \text{C or T} \right)\text{=∑(number of leaves of each grade × corresponding grade)}$$

$$\text{/(total number of leaves × highest grade)}$$

The control efficiency of curative and protective activity I is calculated as follows:

$$\text{Control efficiency I (\%) = (C-T)/C×100\%}$$

In the formula, C and T represent the disease index of the negative control group and the treatment group, respectively.^[4]^

## 1.16 Rice Phytotoxicity Assay

NI6R, NI6R@*β*-CD, NI6R@CB[7], and NI6R@CB[7]@*β*-CD at concentrations of 200 and 500 *μ*g mL^-1^ were uniformly sprayed on adult rice leaves, and DMSO was used as a blank control. Photographs of the lesions of each sample were taken after 7 days of incubation in an artificial climate chamber at 28 °C and 90% humidity.

## 1.17 *In Vivo* Antibacterial Bioassays against Citrus Canker

Solutions of *β*-CD, CB[7], NI6R, NI6R@*β*-CD, NI6R@CB[7], NI6R@CB[7]@*β*-CD, and TC-20%SC at a concentration of 200 *μ*g mL^-1^ were prepared separately. Equivalent DMSO was used as a blank control. Citrus leaves were uniformly punctured using a sterile syringe with 9 holes on the left and right sides of each leaf.

Protective activity experiment: evenly cut filter paper was soaked in the above prepared solutions for 1 h, respectively, after which the filter paper was applied to the traumatized surface of the leaves for 24 h. After the new filter paper was fully moistened with *Xac* cytosol (OD_595 nm_ = 0.01), the new filter paper was applied directly to the traumatized surface.

Curative activity experiment: The uniformly cut filter paper was fully moistened with *Xac* cytosol (OD_595 nm_ = 0.01) and applied to the traumatized surface, and 24 h later, the new filter paper was separately soaked in the above prepared solution for 1 h, after which the filter paper was applied to the traumatized surface of the leaf. After 14 d of incubation in an artificial incubator (28 °C, 90% RH), the diseased portion of the leaf blade was uniformly cut into 100 mg pieces, and then the chlorophyll of the leaf blade was extracted with 10 mL of a mixture of 95% acetone and 95% ethanol (1:1), and the OD_663 nm_ and OD_645 nm_ values were measured, and the formula was calculated as follows:

$$\text{Chlorophyll a =(12.7 × }\text{OD}_{\text{663}}\text{ }\text{- 2.69 × }\text{OD}_{\text{645}}\text{) × 0.1}$$

$$\text{Chlorophyll b = (22.9 × }\text{OD}_{\text{645}}\text{ - 4.68 × }\text{OD}_{\text{663}}\text{) × 0.1}$$

$$\text{Total chlorophyll= (8.02 × }\text{OD}_{\text{663}}\text{ + 20.21 × }\text{OD}_{\text{645}}\text{) × 0.1}$$

$$\text{Relative infected area= Total chlorophyll in the clean water group-}$$

$\text{Total chlorophyll in the experimental group}$

$$\text{Control efficiency I (\%) = (C-T)/C×100\%}$$

In the equation, C and T were infected areas in the clean water group and the treatment group, respectively.^[5]^

## 1.18 *In Vivo* Antibacterial Bioassays against Kiwifruit Canker

Fresh kiwifruit leaves of mature length were taken and rinsed three times with sterile water and placed on filter paper to dry. Then, the leaves were cut into 1.5 cm diameter using a perforator. Protective activity assay: The cut leaves were soaked in 10 mL of *Psa* cytosol (OD_595 nm_ = 0.1) for 1 hour. After air-drying, they were transferred to a new petri dish and soaked for 1 h with 10 mL of 200 *μ*g mL^-1^ of *β*-CD, CB[7], NI6R, NI6R@*β*-CD, NI6R@CB[7], NI6R@CB[7]@*β*-CD, and TC-20%SC solutions, respectively. An equal amount of DMSO was taken as a blank control. For the curative activity experiment, the samples were first dipped with the drug and then with the bacterial solution. The samples were incubated in an artificial climatic chamber (28 °C, 90% RH). 5 d later, the lesion symptoms were observed and photographed for recording, and the area of lesions was measured by Image-J software to evaluate the control efficiency.^[6]^

$$\text{Control efficiency}\left( \text{\%} \right)\text{= (infected area of control group- }$$

$$\text{infected area of treatment group)/infected area of control group}\text{ }\text{× 100\%}$$

## 1.19 Germination Experiment of Rice Seed

First, NI6R, NI6R@*β*-CD, NI6R@CB[7], NI6R@CB[7]@*β*-CD, TC-20%SC, and 90% BT solution treatment solutions were prepared at concentrations of 100 and 200 *μ*g mL^-1^, respectively. After that, each petri dish was filled with 15 mL of treatment solution and 50 sterile whole rice seeds were placed, and three parallels were set up for each sample, with water, *β*-CD, and CB[7] as controls. They were placed in an artificial incubator (temperature: 25 °C, light/dark time: 13 h/11 h, 75% humidity) for 5 d. The treatment solution was changed once a day. Twenty seeds were randomly selected from each fraction to determine the germination rate, and root length and stem length were determined.^[7]^

## 1.20 Earthworm Safety Experiment

The experiment was conducted using a soil mixture bioassay. For this, 200 grams of artificial soil was placed into a 500 mL plastic container. The moisture content was adjusted to 36% of the final weight. In the test soil, concentrations of 2.88 and 4.32 *μ*g g^-1^ were achieved by adding 8 and 12 *μ*g mL^-1^ solutions of NI6R, NI6R@*β*-CD, NI6R@CB[7], and NI6R@CB[7]@*β*-CD, respectively, to the container. Ten mature *Eisenia Fetida* were placed in each container. The containers were sealed with plastic wrap, perforated for ventilation, and placed in an incubator set at 20±2 °C with 80% humidity and a 12-hour night/day photoperiod, earthworm mortality was measured 5 d later.^[8]^

## 1.21 Zebrafish Safety Experiment

The acute toxicity experiment of zebrafish was performed by hydrostatic bioassay without feeding and water change during the experiment. Ten zebrafish were kept in NI6R, NI6R@*β*-CD, NI6R@CB[7], NI6R@CB[7]@*β*-CD solutions at concentrations of 6 and 10 *μ*g mL^-1^, respectively, and a control group of deionized water was set up. The state of the zebrafish was observed every day, and if there was no obvious movement, such as gill flapping and no response to touching the tail, the fish was judged to be dead. The survival of zebrafish after 96 h was recorded.

## 1.22 Statistical Data Processing

IBM SPSS Statistics 27 software was used to statistic analyze the significance difference. Single-factor ANOVA test was used, with lower case letters indicating statistically significant differences between components (*P* < 0.05 adjusted by Waller-Duncan method).

## 2. Synthesis Procedures and Characterization Data for Target Compounds

The synthetic route and structure of target compounds NI6R and NI6S:

**Figure S1.** Synthetic route of title compounds NI6R and NI6S.

**(*R*)-2-((naphthalen-2-yloxy)methyl)oxirane** **(1R)**

A 50 mL round-bottomed flask was stirred with *β*-naphthol (1.0 g, 6.96 mmol), (*R*)-epichlorohydrin (816.47 *μ*L, 10.44 mmol) and 20 mL of DMF, to which KOH (468.57, 8.35 mmol) was added, and stirred for 24 h at normal temperature. The reaction was completed, ethyl acetate (60 mL) was added to the reaction system, and the organic layer was extracted with saturated NH_4_Cl solution (50 mL×3), dried over anhydrous Na_2_SO_4_, and the solvent was removed in vacuum. The intermediate 1R was purified by column chromatography and the eluent was PE:EA (petroleum ether: ethyl acetate) = 40:1 (V/V). A white powdery solid, yield 88.59%; ^1^H NMR (500 MHz, CDCl_3_) *δ* 7.85 – 7.74 (m, 3H, Naph-H), 7.49 (t, *J* = 7.5 Hz, 1H, Naph-H), 7.39 (t, *J* = 7.4 Hz, 1H, Naph-H), 7.24 (dd, *J* = 8.9, 2.2 Hz, 1H, Naph-H), 7.16 (s, 1H, Naph-H), 4.33 (dd, *J* = 10.9, 2.9 Hz, 1H, Naph-O-CH_2_-), 4.03 (dd, *J* = 10.9, 5.8 Hz, 1H, Naph-O-CH_2_-), 3.45 – 3.40 (m, 1H, CH), 2.94 (t, *J* = 4.5 Hz, 1H, -O-CH_2_-), 2.80 (dd, *J* = 4.7, 2.6 Hz, 1H, -O-CH_2_-).^13^C NMR (101 MHz, CDCl_3_) *δ* 156.37, 134.37, 129.75, 129.30, 127.65, 127.04, 126.61, 123.82, 118.76, 106.72, 68.63, 50.05, 44.60.

Intermediate 1S was synthesized according to the synthesis of intermediate 1R.

**(*S*)-2-((naphthalen-2-yloxy)methyl)oxirane (1S)**

A white powdery solid, yield 80.21%; ^1^H NMR (500 MHz, CDCl_3_) *δ* 7.80 – 7.72 (m, 3H, Naph-H), 7.45 (ddd, *J* = 8.2, 6.9, 1.2 Hz, 1H, Naph-H), 7.35 (ddd, *J* = 8.1, 6.9, 1.2 Hz, 1H, Naph-H), 7.19 (dd, *J* = 8.9, 2.6 Hz, 1H, Naph-H), 7.14 (d, *J* = 2.5 Hz, 1H, Naph-H), 4.36 (dd, *J* = 10.9, 3.1 Hz, 1H, Naph-O-CH_2_-), 4.07 (dd, *J* = 10.9, 5.8 Hz, 1H, Naph-O-CH_2_-), 3.44 (ddt, *J* = 5.7, 4.0, 2.9 Hz, 1H, CH), 2.96 (dd, *J* = 4.7, 4.3 Hz, 1H, -O-CH_2_-), 2.83 (dd, *J* = 4.9, 2.7 Hz, 1H, -O-CH_2_-). ^13^C NMR (101 MHz, CDCl_3_) *δ* 156.52, 134.50, 129.68, 129.26, 127.79, 126.92, 126.58, 123.98, 118.92, 106.88, 68.82, 50.26, 44.93.

**(*R*)-1-(1H-imidazol-1-yl)-3-(naphthalen-2-yloxy)propan-2-ol (*R*-NI)**

Intermediate 1R (1.0 g, 4.99 mmol), imidazole (407.99 mg, 5.99 mmol) and K_2_CO_3_ (552.16 mg, 4.0 mmol) were added to a 50 mL round-bottomed flask with stirring, and the reaction was heated to reflux for 16 h at 60°C. The reaction was carried out with the help of the following ingredients. Then, dichloromethane (60 mL) was added to the reaction system, and the organic layer was extracted with saturated NH_4_Cl solution (50 mL×3), dried over anhydrous Na_2_SO_4_, and the solvent was removed in vacuum. The target compound *R*-NI was purified by column chromatography, and the eluent was DCM : MeOH (dichloromethane : methanol) = 40:1 (V/V). A white solid, yield 69.63%; ^1^H NMR (500 MHz, DMSO-*d_6_* ) *δ* 7.84 (d, *J* = 3.2 Hz, 1H, Naph-H), 7.83 (d, *J* = 2.1 Hz, 1H, [imidazole](javascript:;)-H), 7.80 (d, *J* = 8.2 Hz, 1H, Naph-H), 7.61 (s, 1H, Naph-H), 7.48 – 7.43 (m, 1H, Naph-H), 7.37 – 7.33 (m, 1H, Naph-H), 7.30 (d, *J* = 2.4 Hz, 1H, Naph-H), 7.21 (dd, *J* = 8.9, 2.5 Hz, 1H, [imidazole](javascript:;)-H), 7.18 (s, 1H, Naph-H), 6.88 (s, 1H, [imidazole](javascript:;)-H), 5.59 (d, *J* = 5.1 Hz, 1H, -OH), 4.23 (dd, *J* = 13.3, 3.4 Hz, 1H, Naph-O-CH_2_-), 4.15 (dd, *J* = 9.7, 5.6 Hz, 1H, Naph-O-CH_2_-), 4.10 (dd, *J* = 13.3, 6.9 Hz, 1H, CH), 3.95 (qd, *J* = 9.9, 5.2 Hz, 2H, -CH_2_-N-[imidazole](javascript:;)). ^13^C NMR (101 MHz, DMSO-*d*_6_) *δ* 156.33, 138.02, 134.30, 129.44, 128.63, 128.13, 127.60, 126.80, 126.52, 123.76, 120.27, 118.77, 106.81, 69.50, 68.30, 49.32.

Intermediate *S*-NI was synthesized according to the synthesis of intermediate *R*-NI.

**(*S*)-1-(1H-imidazol-1-yl)-3-(naphthalen-2-yloxy)propan-2-ol (*S*-NI)**

A white solid, yield 85.70%; ^1^H NMR (500 MHz, DMSO-*d*_6_) *δ* 7.85 (s, 1H, Naph-H), 7.83 (s, 1H, [imidazole](javascript:;)-H), 7.80 (d, *J* = 8.2 Hz, 1H, Naph-H), 7.61 (s, 1H, Naph-H), 7.46 (t, *J* = 7.5 Hz, 1H, Naph-H), 7.35 (t, *J* = 7.5 Hz, 1H, Naph-H), 7.30 (d, *J* = 2.1 Hz, 1H, Naph-H), 7.23 – 7.19 (dd, 1H, [imidazole](javascript:;)-H), 7.18(s, 1H, Naph-H), 6.88 (s, 1H, [imidazole](javascript:;)-H), 5.57 (d, *J* = 4.9 Hz, 1H, -OH), 4.22 (dt, *J* = 10.3, 5.2 Hz, 1H, Naph-O-CH_2_-), 4.18 – 4.05 (m, 2H, Naph-O-CH_2_-, CH), 4.01 – 3.89 (m, 2H, -CH_2_-N-[imidazole](javascript:;)). ^13^C NMR (101 MHz, DMSO-*d*_6_) *δ* 156.31, 137.98, 134.28, 129.41, 128.61, 128.10, 127.58, 126.77, 126.49, 123.74, 120.23, 118.75, 106.80, 69.49, 68.28, 49.30.

**3,3'-(hexane-1,6-diyl)bis(1-((*R*)-2-hydroxy-3-(naphthalen-2-yloxy)propyl)-1H-imidazol-3-ium) bromide (NI6R)**

The intermediate *R*-NI (300.0 mg, 1.12 mmol), 1,3,5-tris(bromomethyl)benzene (118.6 mg, 486.12 *μ*mol) and 5 mL of acetonitrile were added into a 15 mL pressure-resistant tube and heated and stirred at 70 ℃ for 32 h. After completion of the reaction, the reaction was filtered under vacuum, and then washed with 5 mL of acetonitrile for three times, and with 2 mL of dichloromethane for three times, respectively, 0.5 mL methanol twice, and then dried in an oven for 4 h. The target compound NI6R was obtained. A white powdery solid, yield 88.86%; ^1^H NMR (500 MHz, DMSO-*d*_6_) *δ* 9.22 (s, 2H, imidazole-H), 7.85 (d, *J* = 4.3 Hz, 2H, Naph-H), 7.83 (d, *J* = 3.3 Hz, 2H, imidazole-H), 7.82 – 7.79 (m, 4H, Naph-H, 2H, imidazole-H), 7.48 – 7.45 (m, 2H, Naph-H), 7.37 – 7.34 (m, 4H, Naph-H), 7.18 (dd, *J* = 8.9, 2.5 Hz, Naph-H), 5.88 (d, *J* = 5.0 Hz, 2H, –OH), 4.49 (dd, *J* = 12.8, 1.9 Hz, 2H, -CH-), 4.33 – 4.25 (m, 4H, N-CH_2_-), 4.16 (t, *J* = 7.2 Hz, 4H, -CH_2_-), 4.08 (qd, *J* = 10.1, 4.9 Hz, 4H, N-CH_2_-), 1.74 (s, 4H, -CH_2_-), 1.21 (s, 4H, -CH_2_-). ^13^C NMR (126 MHz, DMSO-*d*_6_) *δ* 156.12, 136.73, 134.23, 129.45, 128.65, 127.60, 126.76, 126.56, 123.84, 123.37, 122.15, 118.60, 106.96, 69.39, 67.21, 52.18, 48.69, 29.17, 24.88. HRMS (ESI) m/z [M-2Br^-^]/2 calcd for C_38_H_44_N4O_4_^2+^ : 310.1676, found: 310.1678.

The target compound NI6S was synthesized according to the synthesis of target compound NI6R.

**3,3'-(hexane-1,6-diyl)bis(1-((*S*)-2-hydroxy-3-(naphthalen-2-yloxy)propyl)-1H-imidazol-3-ium) bromide (NI6S)**

A white powdery solid, yield 84.70%; ^1^H NMR (500 MHz, DMSO-*d*_6_) *δ* 9.23 (s, 2H, imidazole-H), 7.81 (d, *J* = 4.0 Hz, 2H, Naph-H), 7.80 (s, 1H, imidazole-H), 7.78 (dt, *J* = 5.2, 1.7 Hz, 2H, imidazole-H, 4H, Naph-H), 7.77 (s, 1H, imidazole-H), 7.43 (ddd, *J* = 8.1, 7.0, 1.1 Hz, 2H, Naph-H), 7.34 – 7.30 (m, 4H, Naph-H), 7.15 (dd, *J* = 8.9, 2.6 Hz, 2H, Naph-H), 5.80 (d, *J* = 5.1 Hz, 2H, –OH), 4.46 (dd, *J* = 12.8, 1.9 Hz, 2H, -CH-), 4.30 – 4.22 (m, 4H, N-CH_2_-), 4.14 (t, *J* = 7.2 Hz, 4H, -CH_2_-), 4.05 (qd, *J* = 10.1, 4.9 Hz, 4H, N-CH_2_-), 1.75 – 1.68 (m, 4H, -CH_2_-), 1.19 (s, 4H, -CH_2_-). ^13^C NMR (126 MHz, DMSO-*d*_6_) *δ* 156.16, 136.73, 134.26, 129.48, 128.68, 127.62, 126.80, 126.58, 123.86, 123.38, 122.18, 118.64, 106.99, 69.43, 67.25, 52.19, 48.73, 29.19, 24.88. HRMS (ESI) m/z [M-2Br^-^]/2 calcd for C_38_H_44_N4O_4_^2+^: 310.1676, found: 310.1676.

# 3. ^1^H NMR, ^13^C NMR, HPLC, and HRMS Spectra of Target Compounds


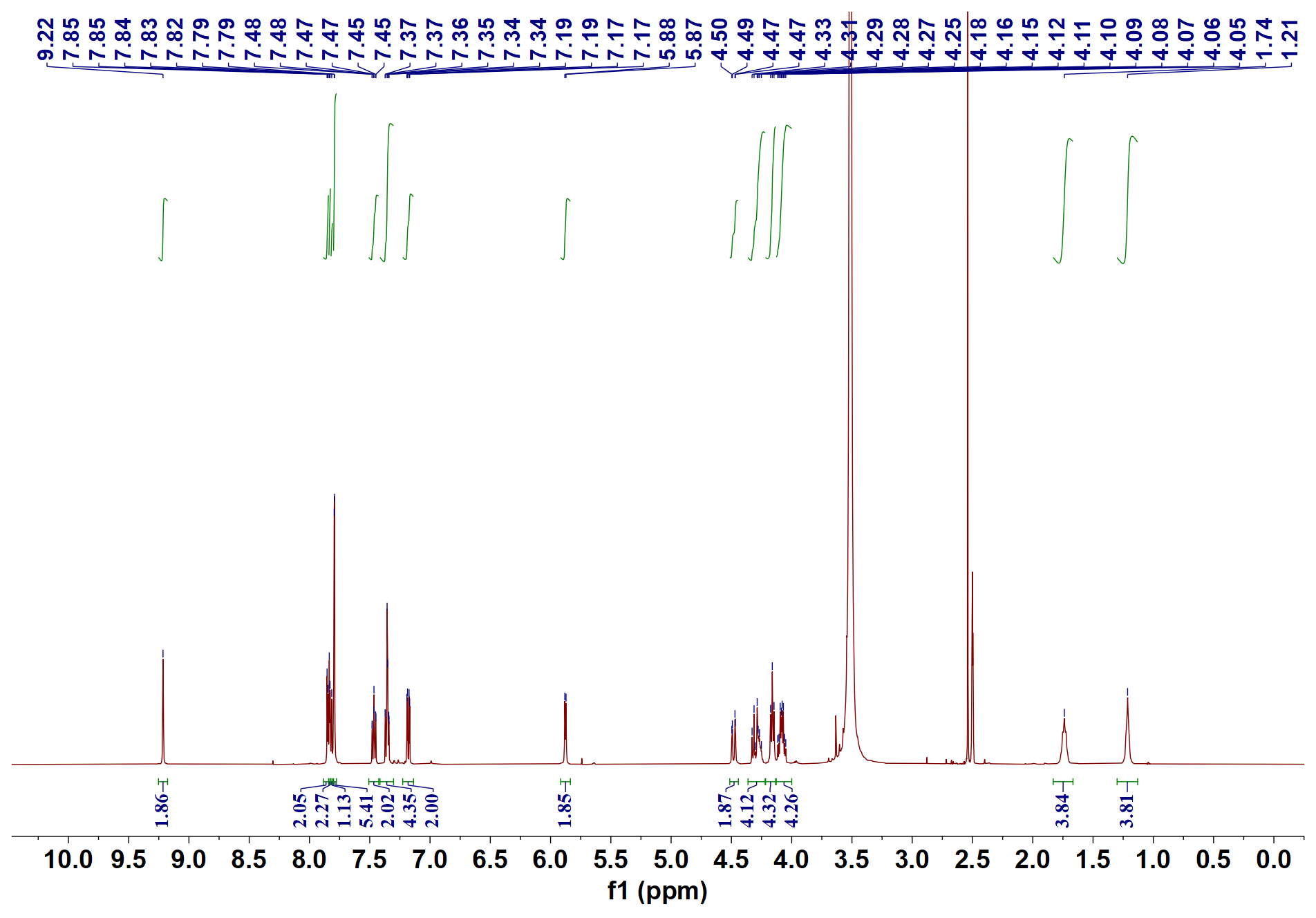


**Figure S2.** ^1^H NMR spectrum (500 MHz, DMSO-*d_6_*) of compound NI6R.


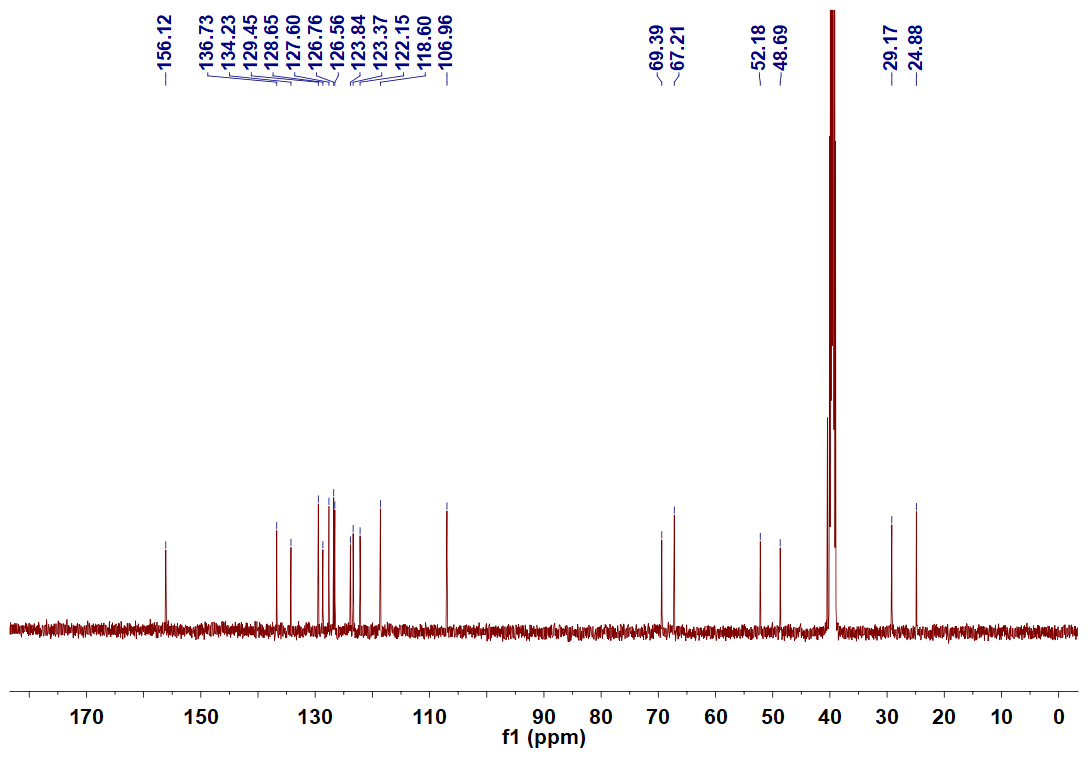


**Figure S3.** ^13^C NMR spectrum (126 MHz, DMSO-*d_6_*) of compound NI6R.


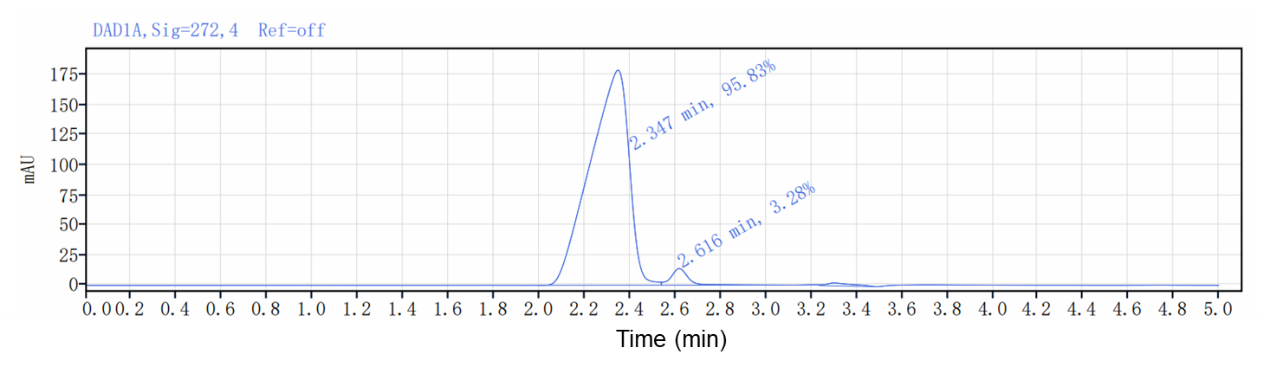


**Figure S4.** Representative HPLC profile of NI6R at an effective concentration of 200 *μ*g mL^-1^. Conditions: 300 Exlend-C18×250 mm×5 *µ*m, methanol/water = 80/20, 0.8 mL min^-1^, injection volume 10.0 *μ*L, 272 nm, Rt = 2.347 min.

**Figure S5.** HRMS spectrum of compound NI6R.


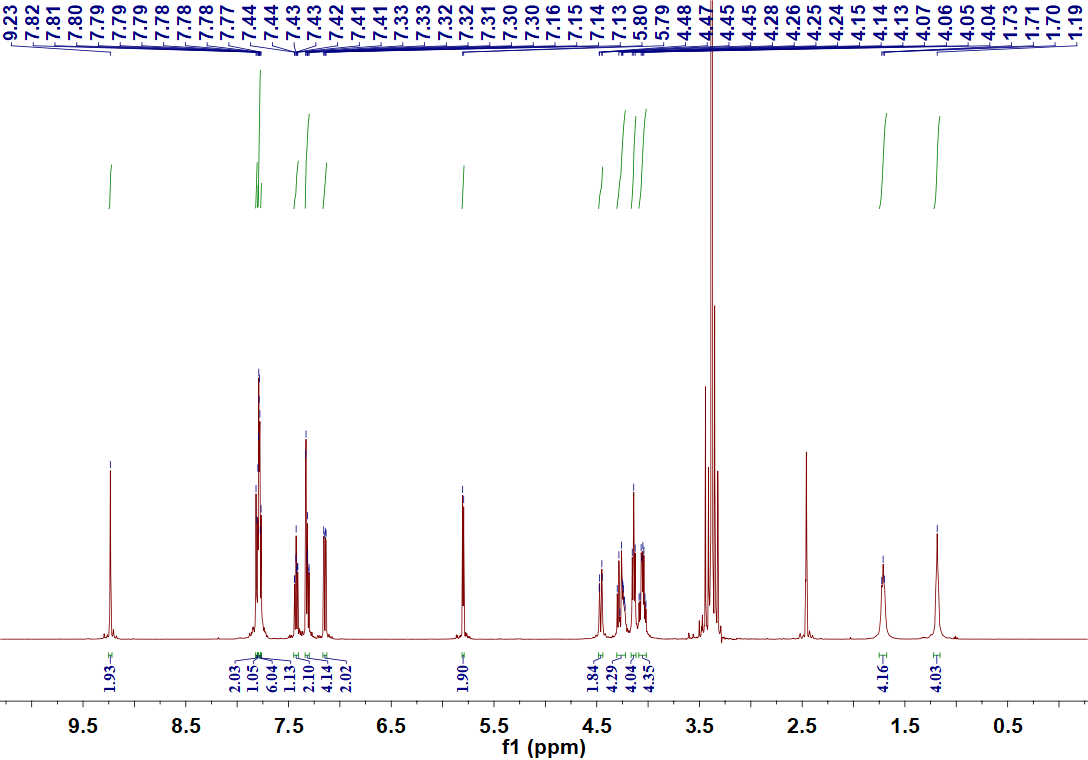


**Figure S6.** ^1^H NMR spectrum (500 MHz, DMSO-*d_6_*) of compound NI6S.


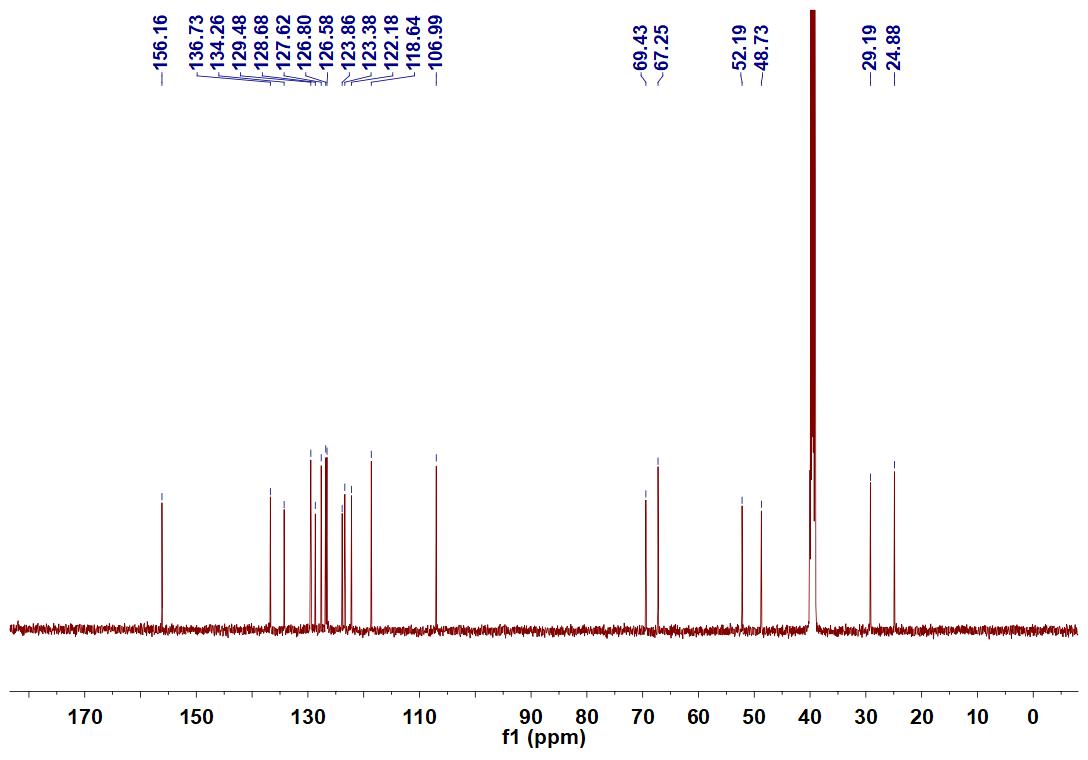


**Figure S7.** ^13^C NMR spectrum (126 MHz, DMSO-*d_6_*) of compound NI6S.

**Figure S8.** HRMS spectrum of compound NI6S.

# 4. Supplementary Figures and Tables

## 4.1 Characterization of NI6R@CB[7]

**
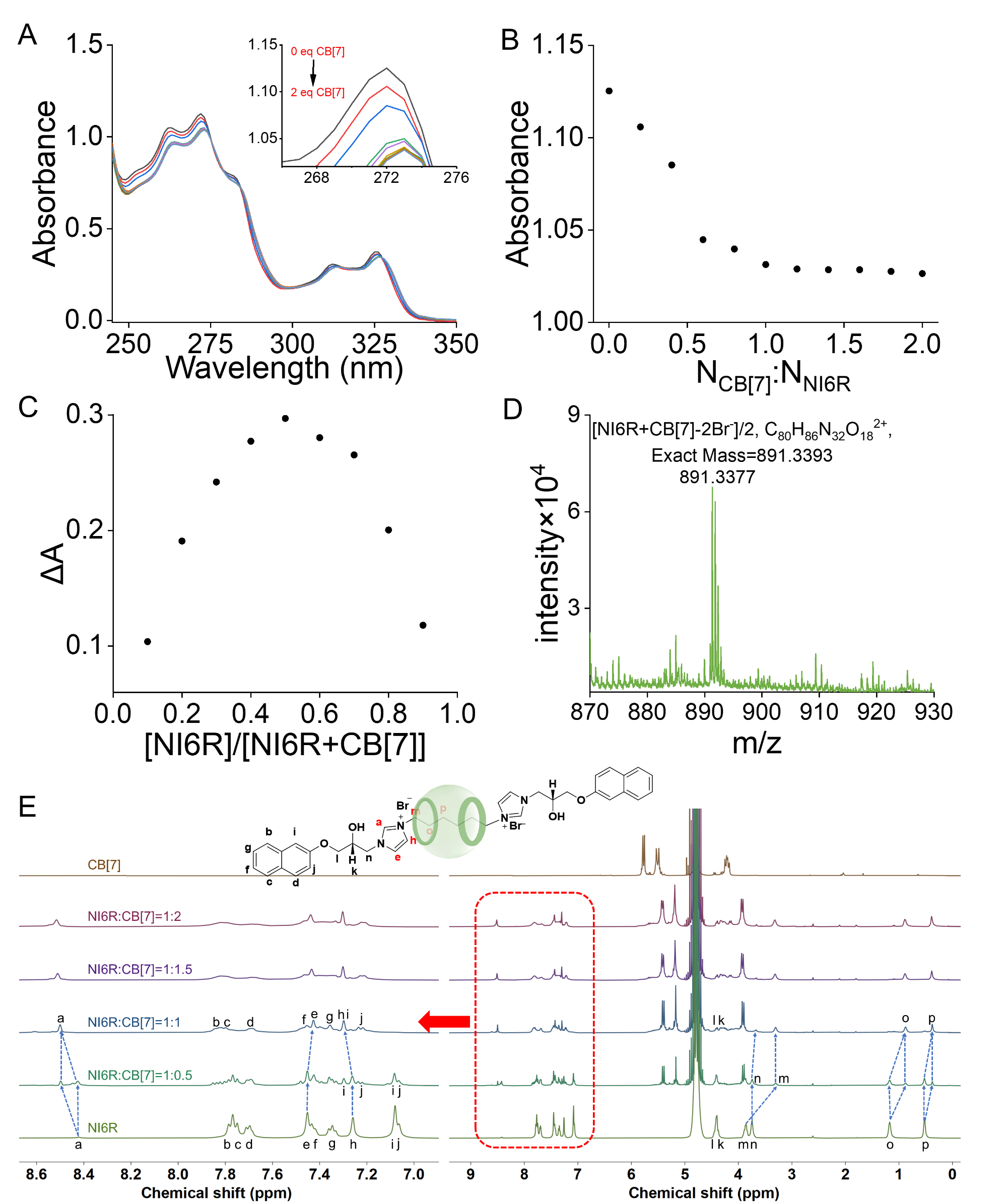
**

**Figure S9.** A) UV-vis absorption of NI6R (0.15 mM) after addition of CB[7] (0.015 ~ 0.30 mM) in aqueous solution; B) UV-vis absorption at 272 nm of NI6R (0.15 mM) after addition of different equivalents of CB[7]; C) Job's plot of *Δ*A at 272 nm at a total concentration of 0.15 mM of NI6R and CB[7] in solution; D) The HRMS mass spectrum of NI6R@CB[7] (1:1). The strong peak found at m/z 891.3377 (calculated for [NI6R+CB[7]-2Br^-^]/2, C_80_H_86_N_32_O_18_^2+^, exact mass = 891.3393); E) ^1^H NMR spectra of NI6R (2.0 mM), CB[7] and NI6R@CB[7] in D_2_O at different molar ratios (1:0.5, 1:1, 1:1.5, 1:2).

## 4.2 Characterization of NI6R@*β*-CD

**
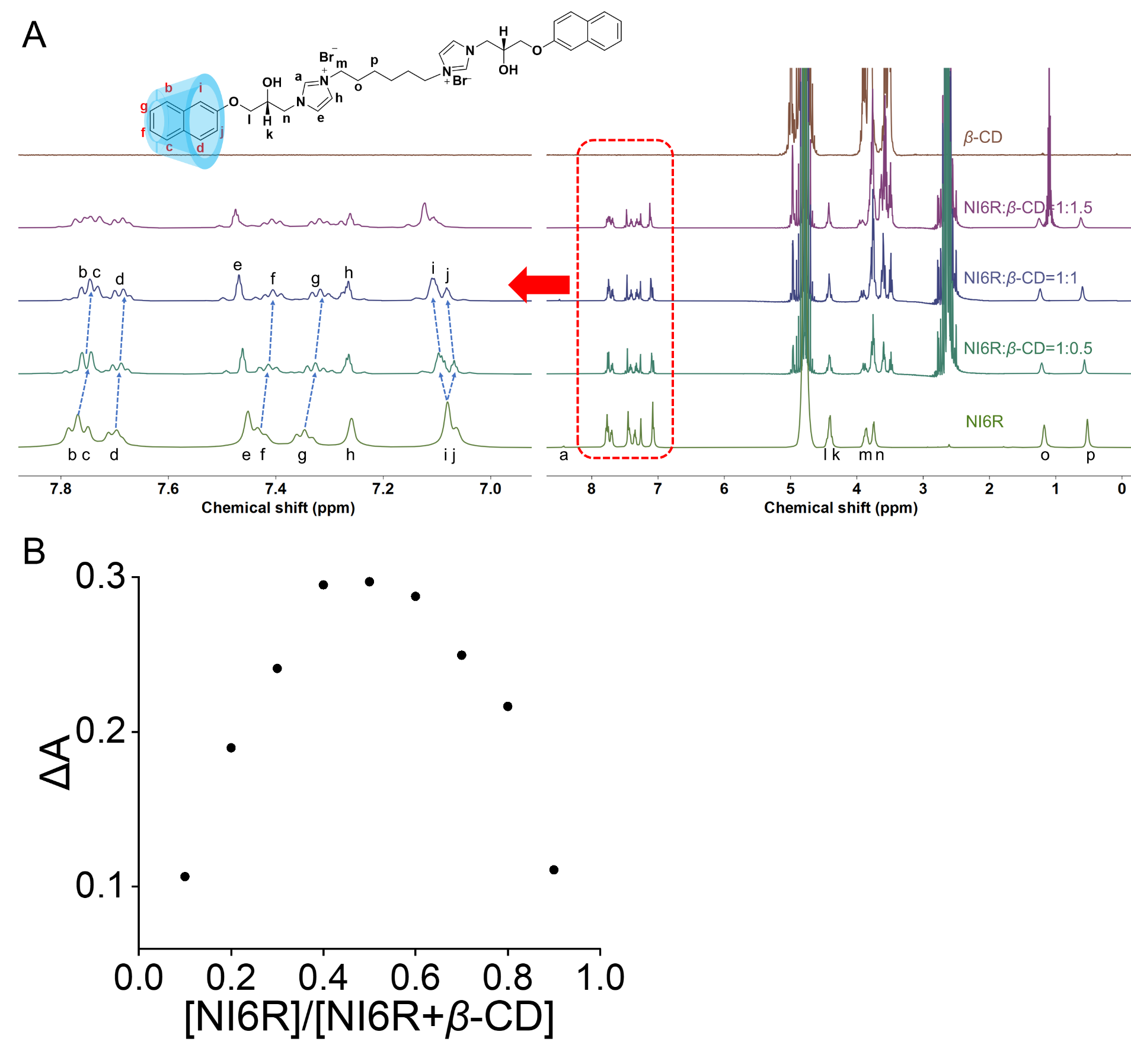
**

**
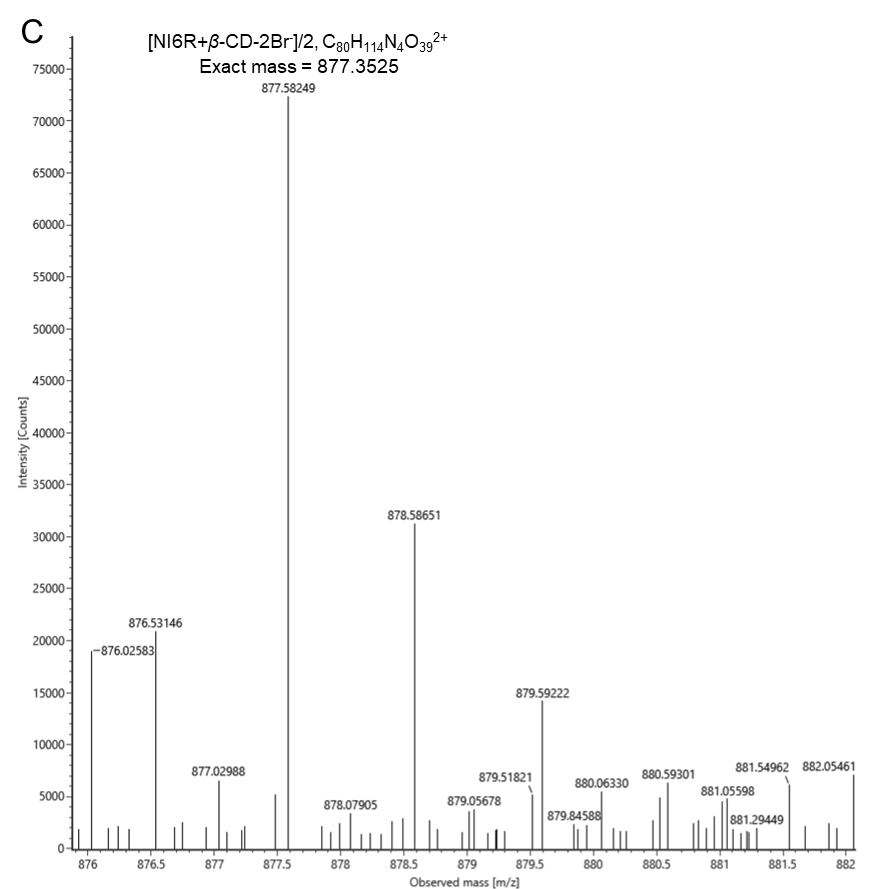
**

**Figure S10.** A) ^1^H NMR spectra of NI6R (2.0 mM), *β*-CD and NI6R@*β*-CD in D_2_O at different molar ratios (1:0.5, 1:1, 1:1.5). B) Job's plot of ΔA at 272 nm at a total concentration of 0.15 mM of NI6R and *β*-CD in solution. C) The HRMS mass spectrum of NI6R@*β*-CD (1:1). The strong peak found at m/z 877.5825 (calculated for [NI6R+*β*-CD-2Br^-^]/2, C_80_H_114_N_4_O_39_^2+^, exact mass = 877.3525).

## 4.3 HRMS Spectrum of Supramolecular Complex NI6R@CB[7]@*β*-CD

**Figure S11.** The HRMS mass spectrum of NI6R@CB[7]@*β*-CD (1:1:1). The strongest peak found at m/z 973.2930 (calculated for [NI6R+CB[7]+*β*-CD-2Br^-^+H^+^]/3, C_122_H_157_N_32_O_53_^3+^, exact mass = 973.0197).

## 4.4 ^1^H-^1^H ROESY Spectra of NI6R, NI6R@CB[7] and NI6R@CB[7]@*β*-CD


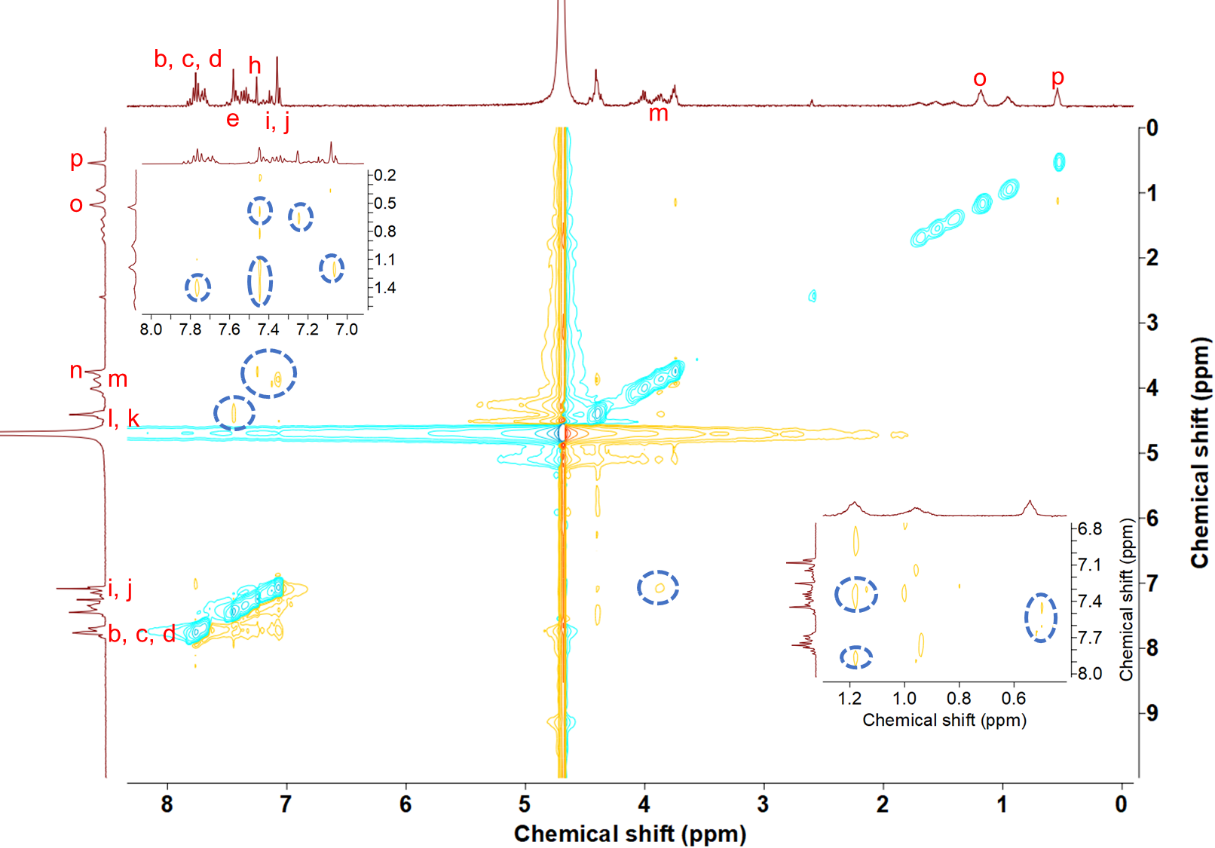


**Figure S12**. ^1^H-^1^H ROESY spectrum of NI6R in D_2_O (C_NI6R_ = 2.0 mM, 400 MHz, 298 K). The blue circles indicate the associated signals.


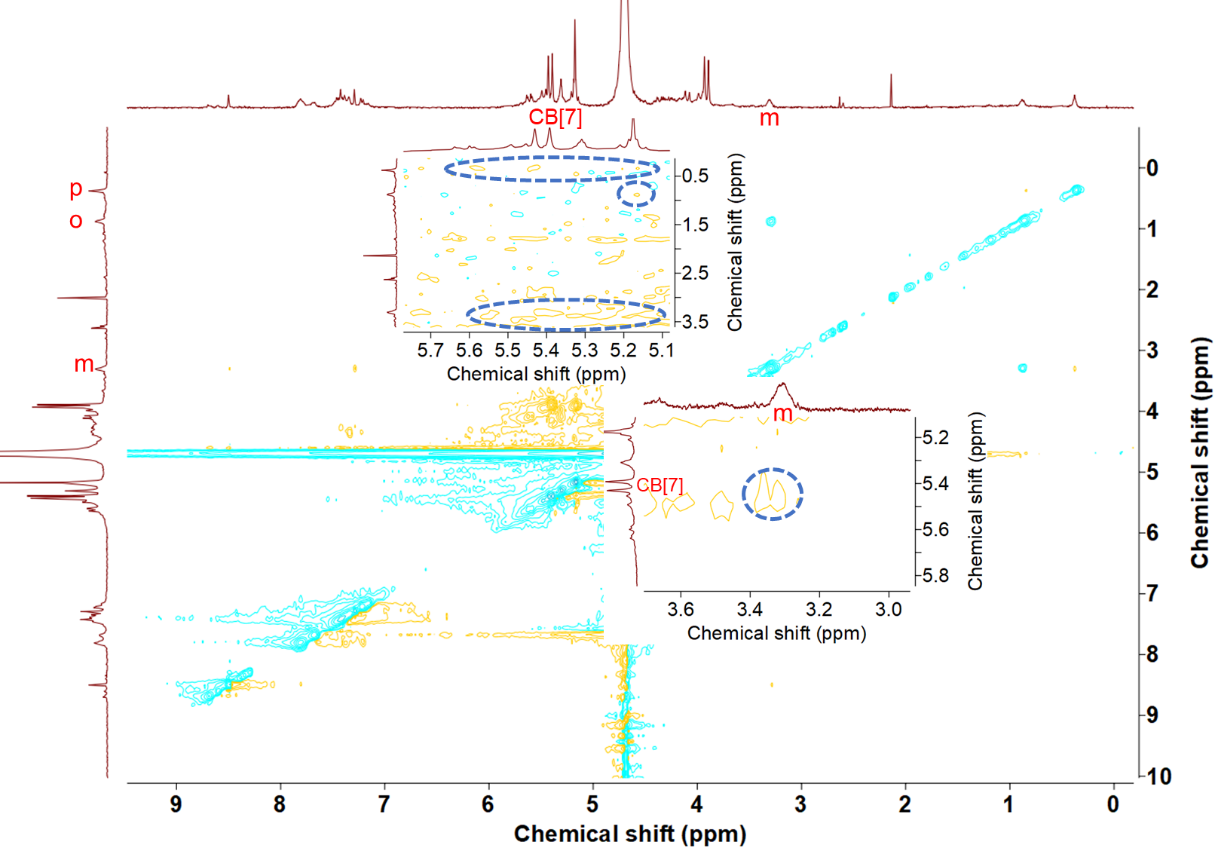


**Figure S13**. ^1^H-^1^H ROESY spectrum of NI6R@CB[7] in D_2_O (C_NI6R@CB[7]_ = 2.0 mM, molar ratio of NI6R : CB[7] = 1:1, 400 MHz, 298 K). The blue circles indicate the associated signals.


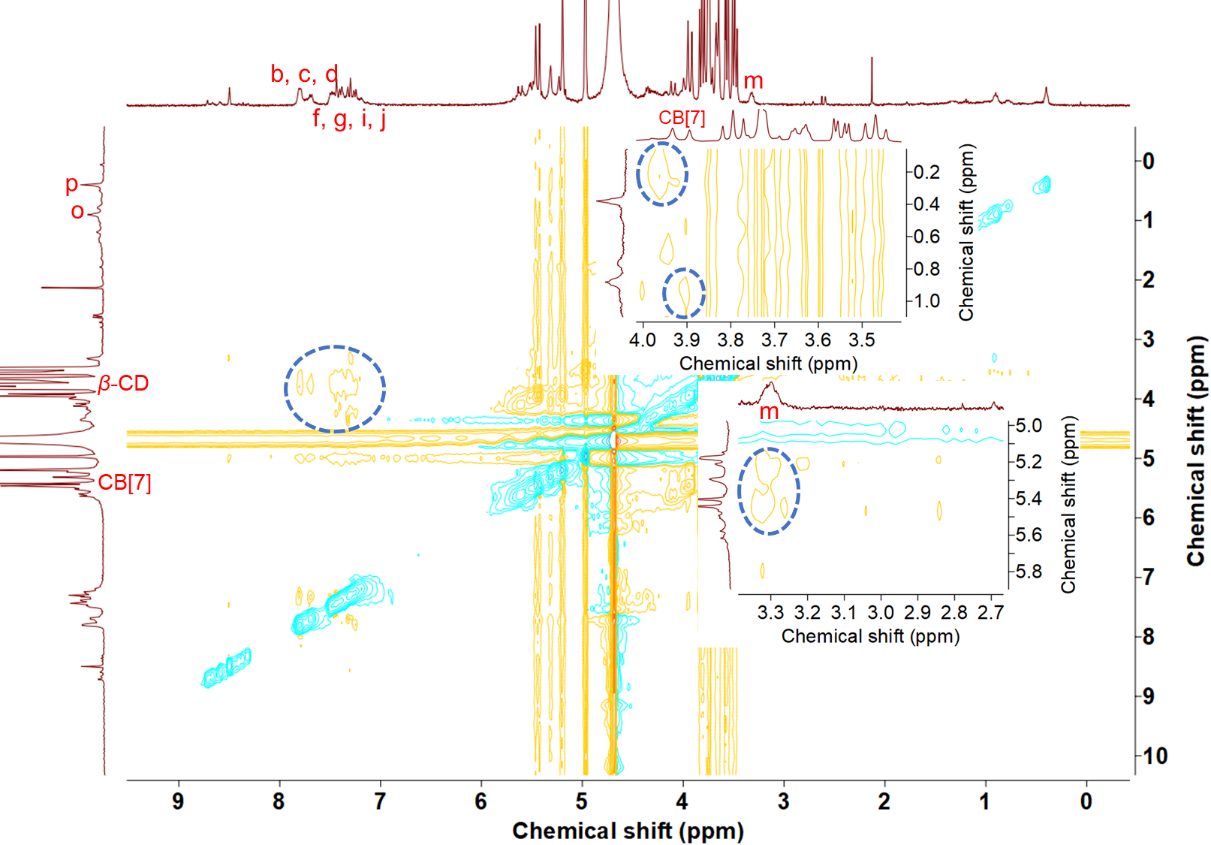


**Figure S14**. ^1^H-^1^H ROESY spectrum of NI6R@CB[7]@*β*-CD in D_2_O (C_NI6R@CB[7]@_*_β_*_-CD_ = 2.0 mM, molar ratio of NI6R : CB[7] : *β*-CD = 1:1:1, 400 MHz, 298 K). The blue circles indicate the associated signals.

## 4.5 2D Diffusion Ordered Spectroscopy (DOSY) of NI6R, NI6R@CB[7] and NI6R@CB[7]@*β*-CD.

**
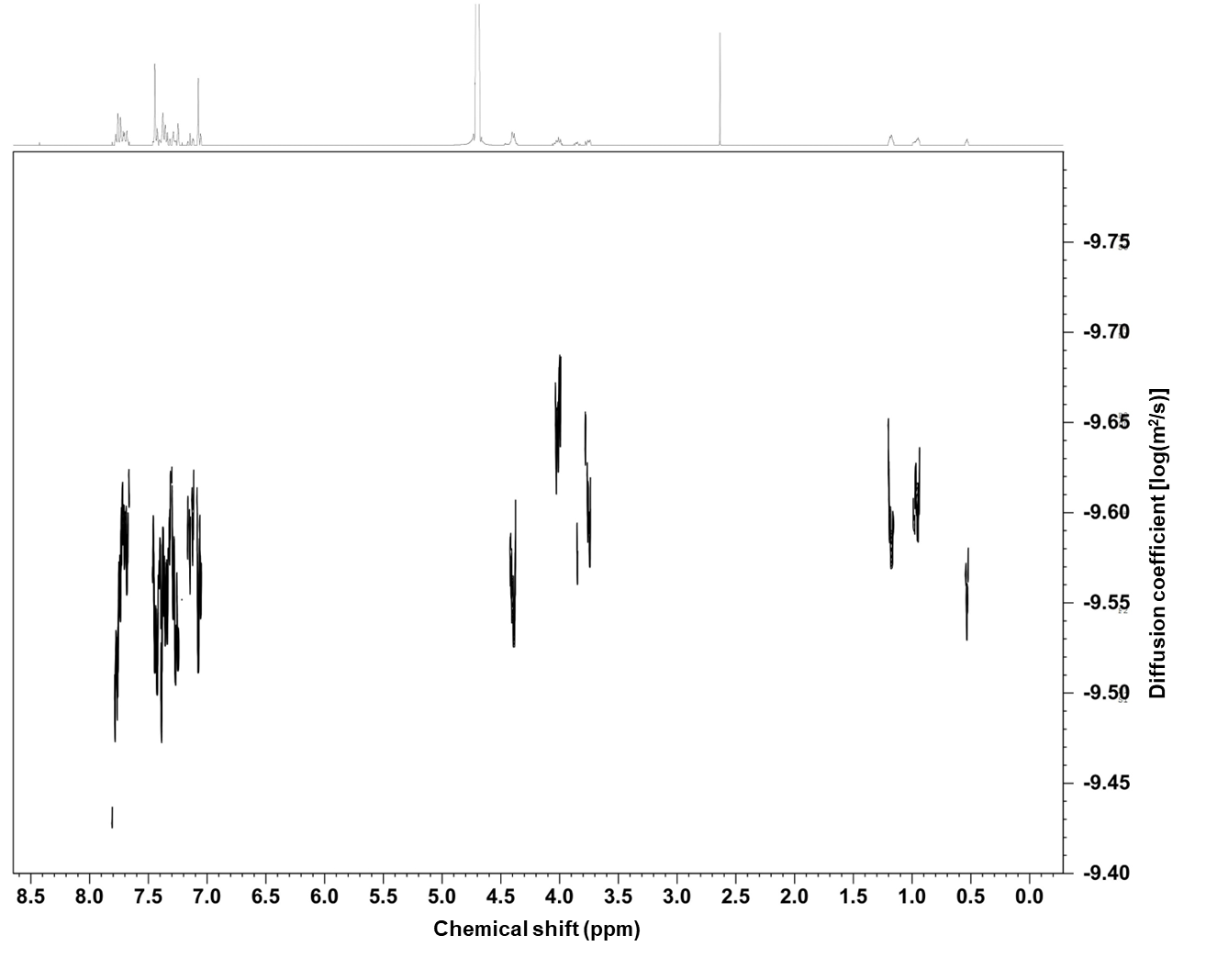
**

**Figure S15**. 2D DOSY spectrum of NI6R in D_2_O (C_NI6R_ = 2.0 mM, 400 MHz, 298 K, *D* = -9.54 lg(m^2^ s^-1^) = 2.88×10^-10^ m^2^ s^-1^).


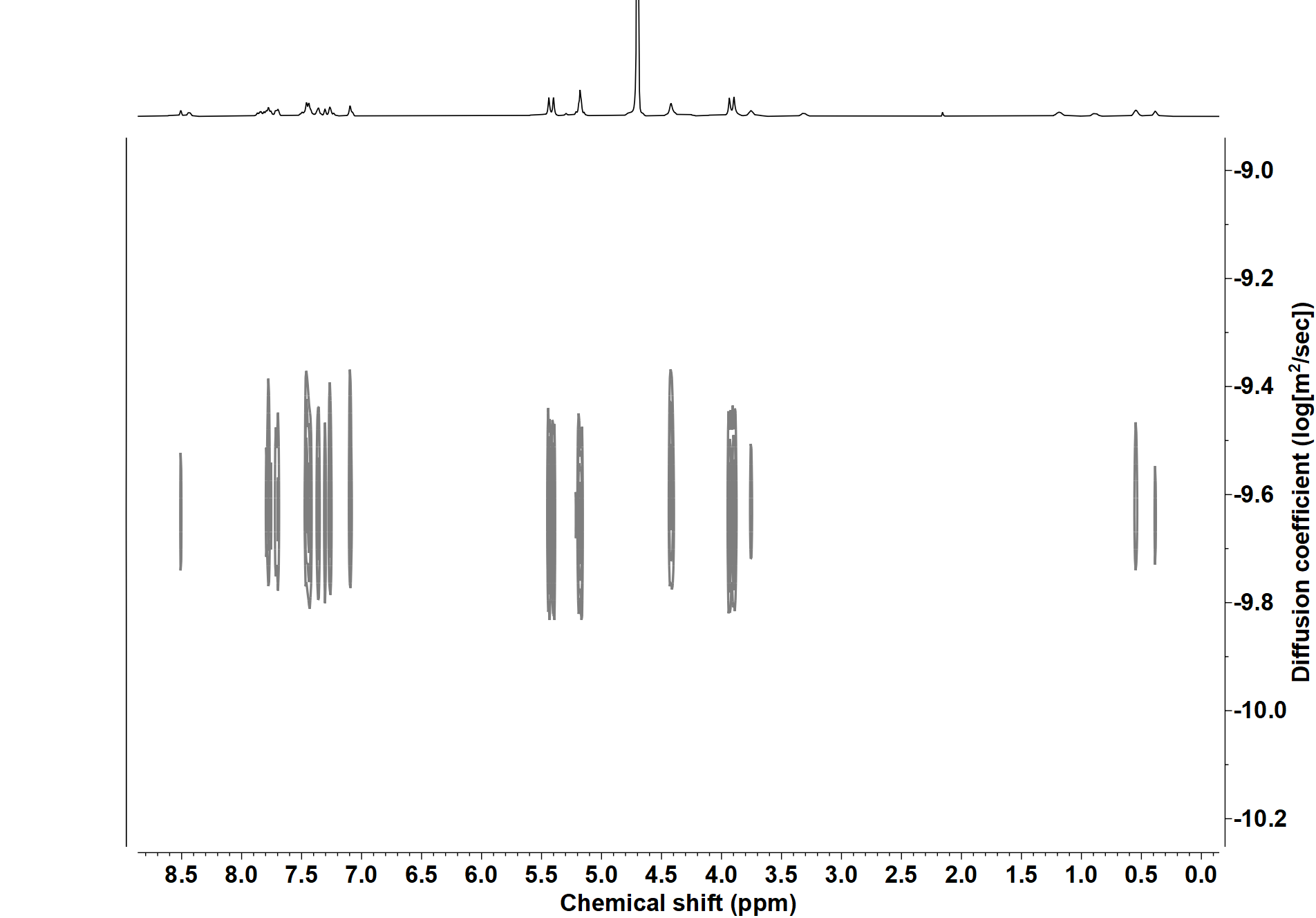


**Figure S16**. 2D DOSY spectrum of NI6R@CB[7] in D_2_O (C_NI6R@CB[7]_ = 2.0 mM, molar ratio, NI6R : CB[7] = 1:1, 400 MHz, 298 K, *D* = -9.63 lg(m^2^ s^-1^) = 2.34×10^-10^ m^2^ s^-1^).


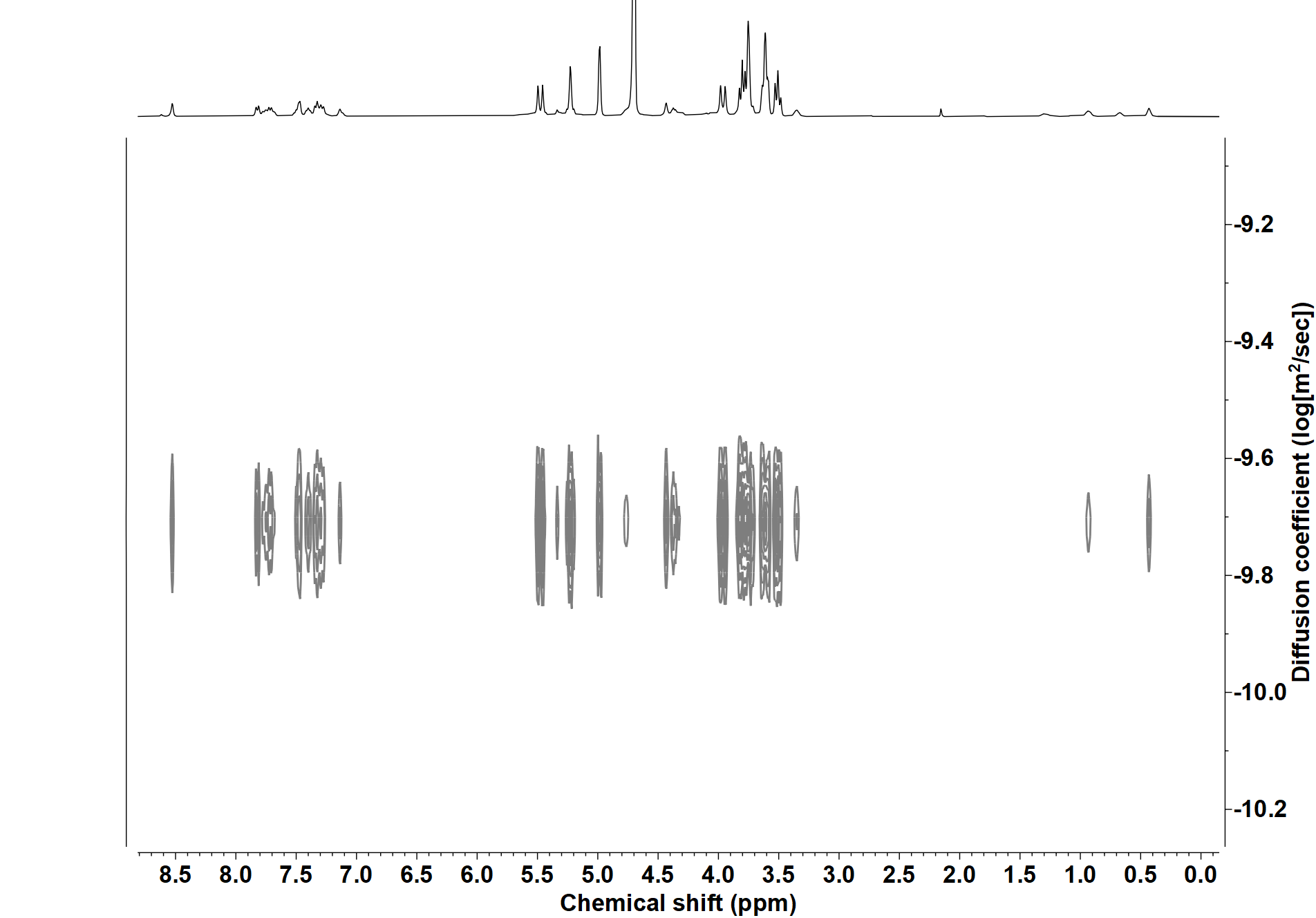


**Figure S17**. 2D DOSY spectrum of NI6R@CB[7]@*β*-CD in D_2_O (C_NI6R@CB[7]@_*_β_*_-CD_ = 2.0 mM, molar ratio of NI6R : CB[7] : *β*-CD = 1:1:1, 400 MHz, 298 K, *D* = -9.72 lg(m^2^ s^-1^) = 1.91×10^-10^ m^2^ s^-1^).

## 4.6 UV-vis Spectra of Different Concentrations of NI6R in Water and Schematic Representation of the Lowest Energy in Chem 3D


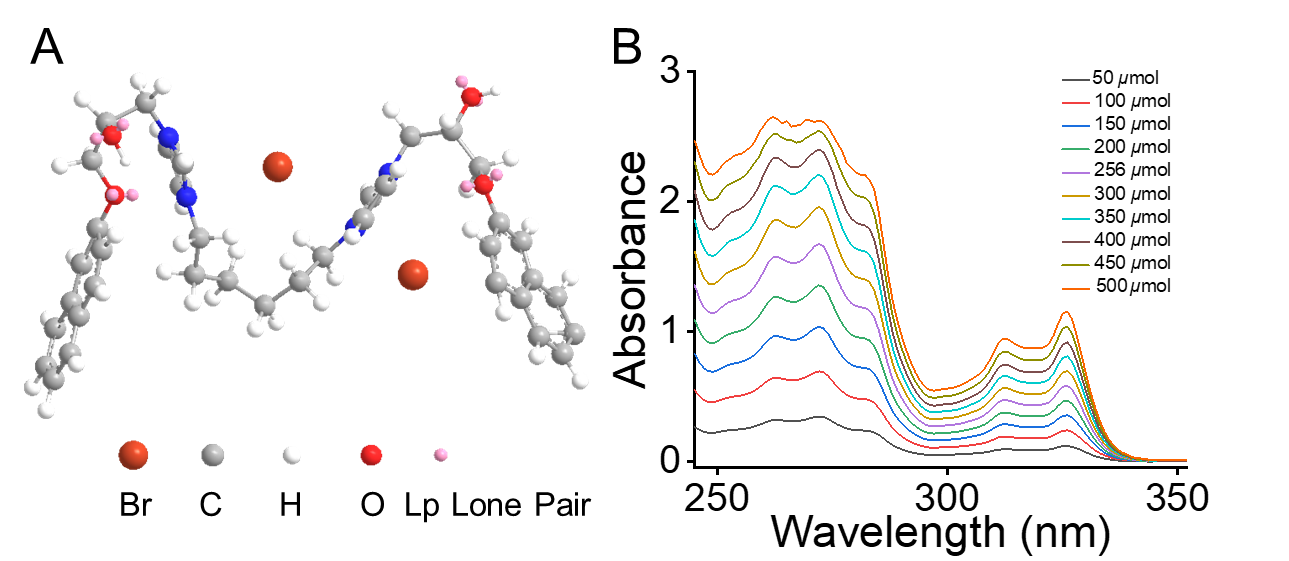


**Figure S18**. A) Schematic diagram of the optimal molecular configuration of NI6R using Chem 3D. B) UV-vis spectra of different concentrations of NI6R in water.

## 4.7 Assembly Mechanisms of Supramolecular Building Blocks NI6R and NI6R@CB[7]

**
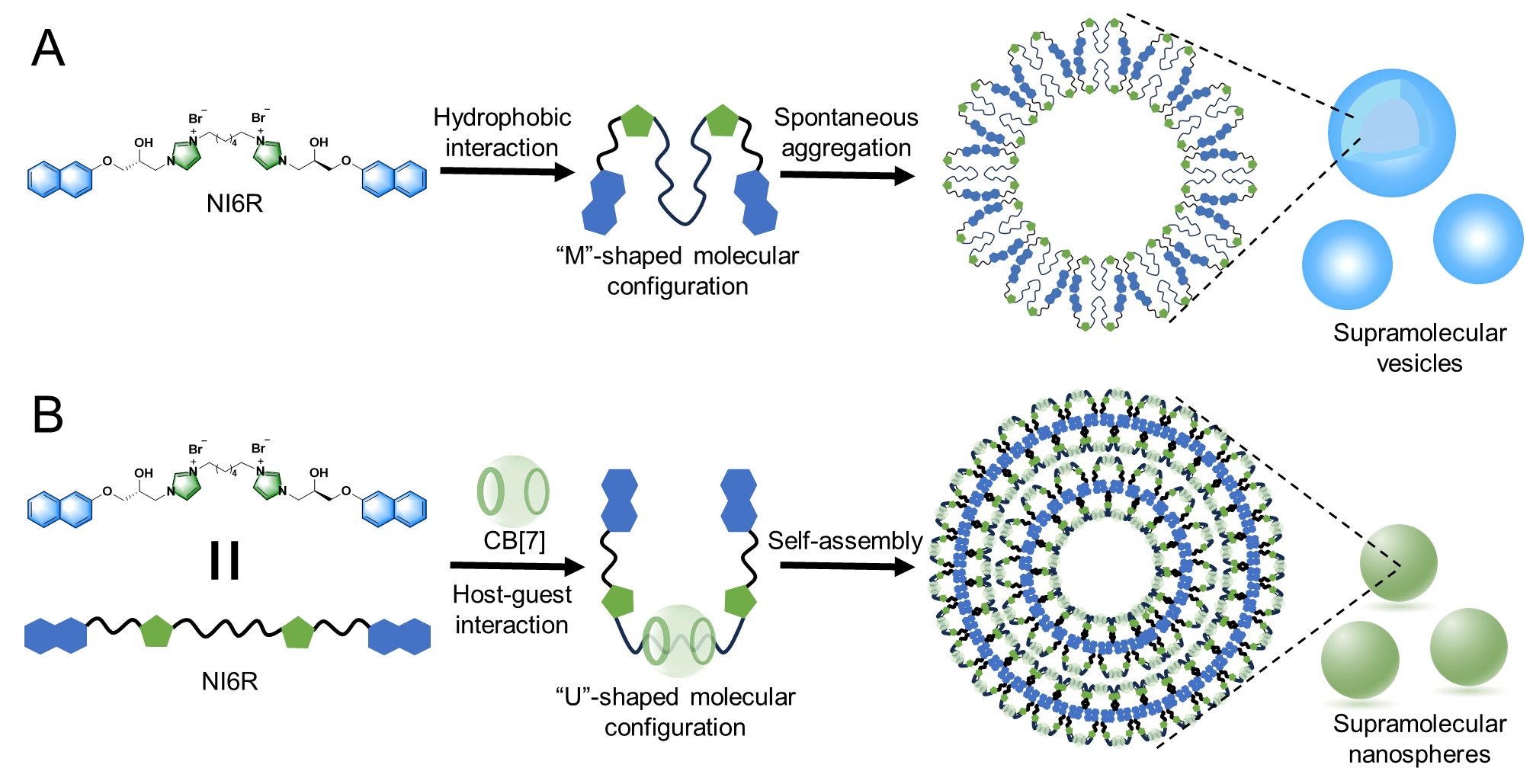
**

**Figure S19.** A) The possible assembly mechanism of NI6R: NI6R is a typical “M”-shaped supramolecular amphiphile, it will voluntarily aggregate together to give vesicular architectures. B) The possible assembly mechanism of NI6R@CB[7]: After loading NI6R with CB[7], a new binary host-guest building unit (NI6R@CB[7]) will adapt a “U”-shaped molecular configuration and affords supramolecular nanospheres.

## 4.8 Biofilm Inhibition Test

**Figure S20.** *Xoo*-biofilm content values at OD_570 nm_.
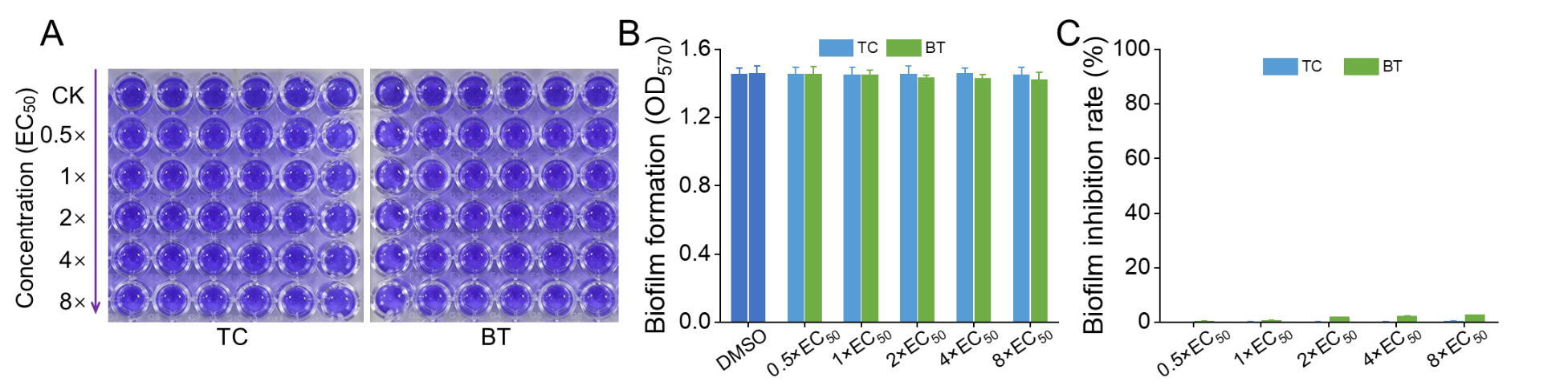


**Figure S21.** A) Biofilm inhibition results using crystal violet staining after 48 hours of co-incubation with varying doses (0.5~8.0×EC_50_, EC_50_ = 1.11 *μ*g mL^-1^) of commercial bactericides TC and BT, while DMSO (0.18%, V/V) was used as the control group. B) *Xoo*-biofilm content values at OD_570 nm_. C) The calculated biofilm inhibition rates at different concentrations of TC and BT.

**Figure S22.** Relative red fluorescence intensity from CLSM 3D images of NI6R, NI6R@*β*-CD, NI6R@CB[7], NI6R@CB[7]@*β*-CD were analyzed using Image-J software.

## 4.9 Biofilm Formation at Different Periods

**Figure S23.** The *Xoo* biofilm formation at different periods.

## 4.10 Crystal Violet Staining for Biofilm Eradication

**
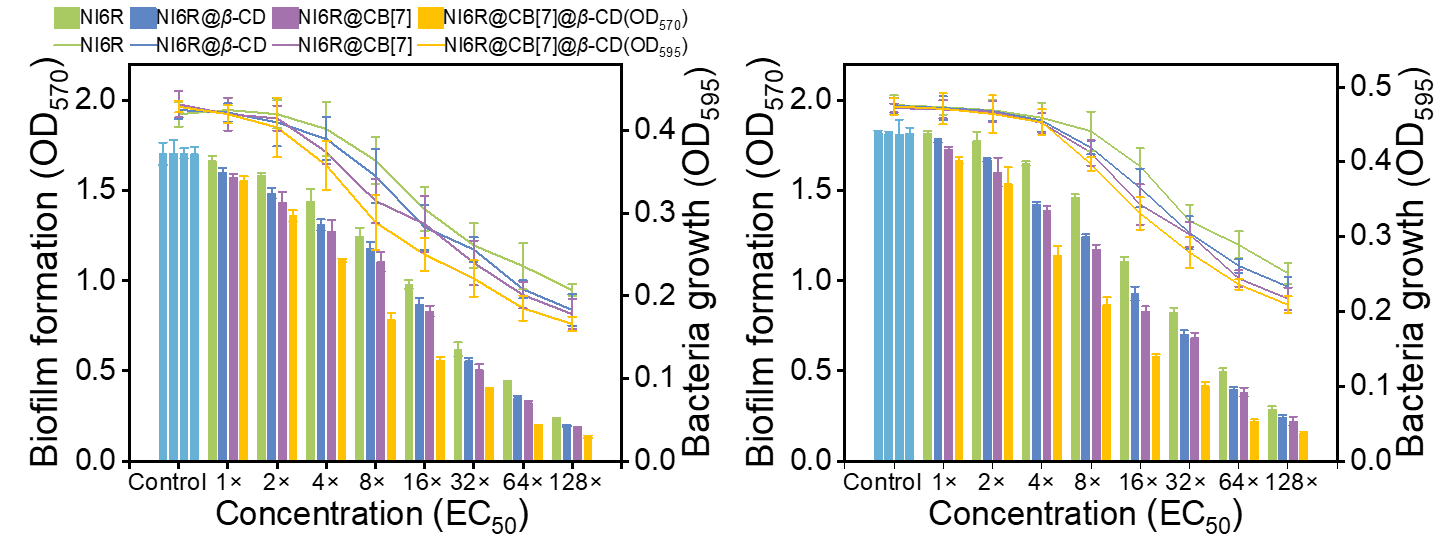
**

**Figure S24.** The effect of the supramolecular complexes on the destruction of pre-established *Xoo*-biofilms was determined by determining the OD_570 nm_ value and OD_595 nm_ value.


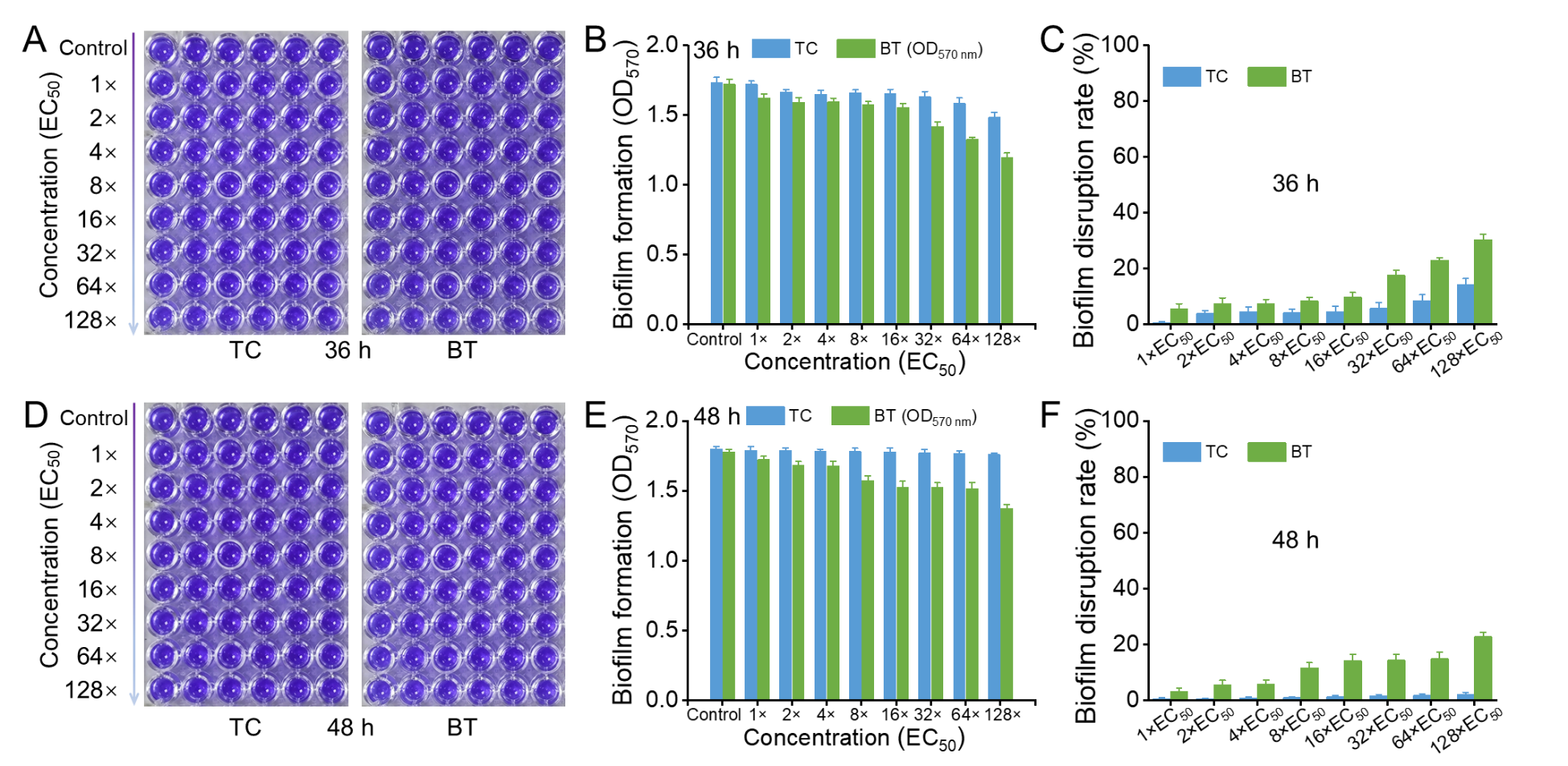


**Figure S25.** A) Crystal violet staining used to assess the biofilm eradication effects of commercial bactericides: *Xoo* biofilms were established in 96-well plates for 36 hours, followed by exposure to different doses of TC and BT for an additional 24 hours before staining. B-C) The relevant OD_570 nm_ values and biofilm destruction rates at different concentrations. D) Crystal violet staining used to assess the biofilm eradication effects of commercial bactericides: *Xoo* biofilms were established in 96-well plates for 48 hours, followed by exposure to different doses of TC and BT for an additional 24 hours before staining. E-F) The relevant OD_570 nm_ values and biofilm destruction rates at different concentrations (1.0~128×EC_50_, EC_50_ = 1.11 *μ*g mL^-1^).

## 4.11 Survival Rate of *Xoo* Bacteria Enclosed by Biofilm

**Figure S26.** Survival rates of biofilm-enclosed *Xoo* bacteria on agar plates treated with NI6R, NI6R@*β*-CD, NI6R@CB[7] and NI6R@CB[7]@*β*-CD at different concentrations.

## 4.12 OD_490 nm_ Value of Exopolysaccharides Solution

**Figure S27.** OD_490 nm_ of various agents at different doses was determined by the phenol-sulfuric acid method.

## 4.13 The Transcriptional Level of the Interrelated *Gum* Gene Cluster that Can Regulate the Synthesis and Transport of EPS in *Xoo*

**Figure S28.** Expression of *gum* genes in response to NI6R, NI6R@*β*-CD, NI6R@CB[7], NI6R@CB[7]@*β*-CD treatment at an effective concentration of 4.44 *μ*g mL^-1^. The error bars represent the standard error of the means of three independent replicates of qRT-PCR analysis. Different letters indicate significant differences between treatments within the same gene at *P* < 0.05.

Quantitative RT-PCR analysis and Experimental method: For the quantitative RT-PCR analysis,^[9]^ total RNA of the *Xanthomonas oryzae* pv*. oryzae* (*Xoo*) was extracted using an MolPure Bacterial RNA Kit (Yeasen, China). First-strand cDNA was synthesized cDNA using 100 ng of total RNA in a 20 *μ*L volume with FastKing gDNA Dispelling RT SuperMix (Tiangen, China). The quantitative RT-PCR was performed on a QuantStudio 5 real-time fluorescence quantitative PCR system (Thermo Fisher Scientific, Waltham, MA, USA). The cDNA was amplified using SYBR Green Master Mix (Yeasen, China). The *gyrB* gene was used as internal control, and the gene expression levels in three biological replicates were calculated using the 2^-ΔΔCt^ method.^[10]^ The primers used for real-time PCR are listed in Table S5.

**Table S5.** Primer Sequences for qRT-PCR

| Gene | Forward prinere | Reverse primere |
| --- | --- | --- |
| *gumB* | GCCATATTTCGTTGCCGCTT | GGAACACGATGACATTGCCG |
| *gumC* | GTTTGCGGAACAACGAGCTT | CGTAGGCACATCTGCGGTAT |
| *gumD* | GTTGCCTGTTGAGCGAACTG | TGGTTCAAAAAGCCACGCAG |
| *gumE* | TGAGCTGACGTTGCTGGTAG | CTGACGATCAAGGCAATGCG |
| *gumG* | ACTCTCTCCAACGCATGGTG | GCCCGACAACACGAAAAACA |
| *gumK* | GAAATGAAGCACGCCGAGAC | CCGCAATAACGGAATCAGCG |
| *gumM* | CTATTCCATGCGTTGGCAGC | ATACGGAATCAGGTCGGTGC |
| *gyrB* | TTCCTCAATTCCGGCGTCAA | CATGGTTTCCTGGTAGGCGT |

## 4.14 Length of Leaf Lesion in Rice Pathogenicity

**Figure S29.** Leaf lesion length of rice treated with NI6R, NI6R@*β*-CD, NI6R@CB[7], and NI6R@CB[7]@*β*-CD at a concentration of 4.44 *μ*g mL^-1^ (4×EC_50_) by the leaf-clipping method.

## 4.15 Investigation of Droplet Splashing on Rice Leaves


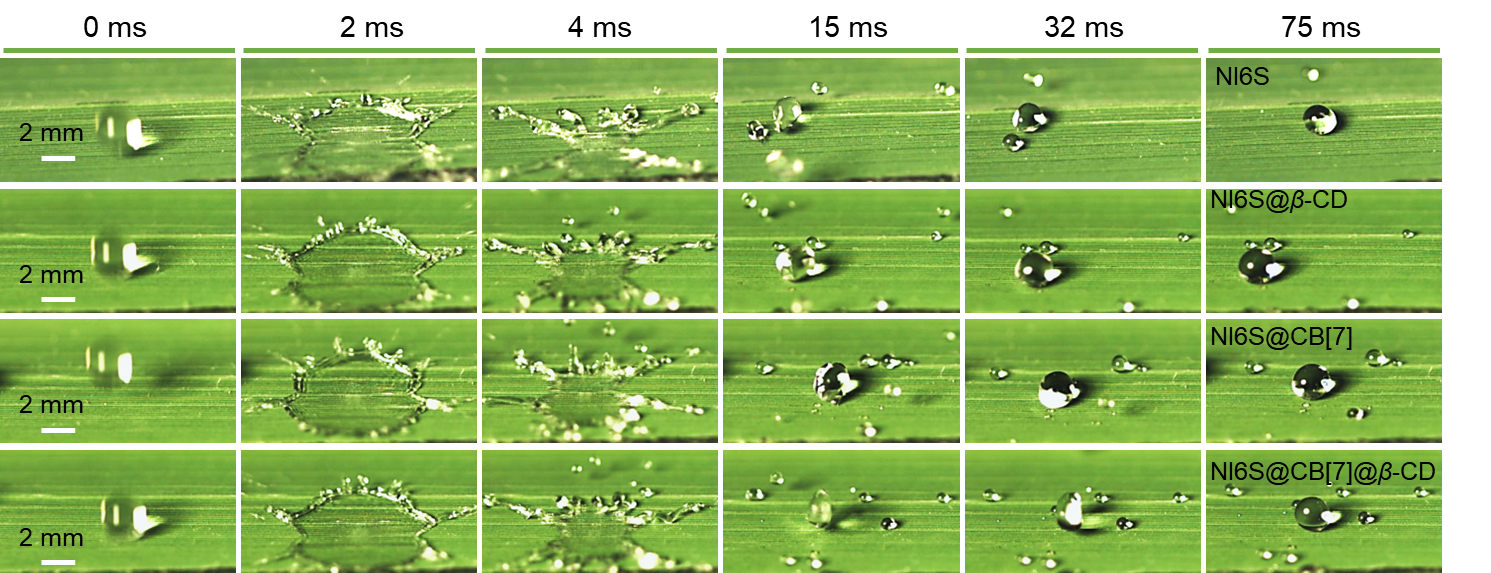


**Figure S30.** High-speed camera images of NI6S, NI6S@*β*-CD, NI6S@CB[7], and NI6S@CB[7]@*β*-CD droplets during splashing at a height of 40 cm on rice leaves.

## 4.16 Investigation of Droplet Bouncing on Rice Leaves


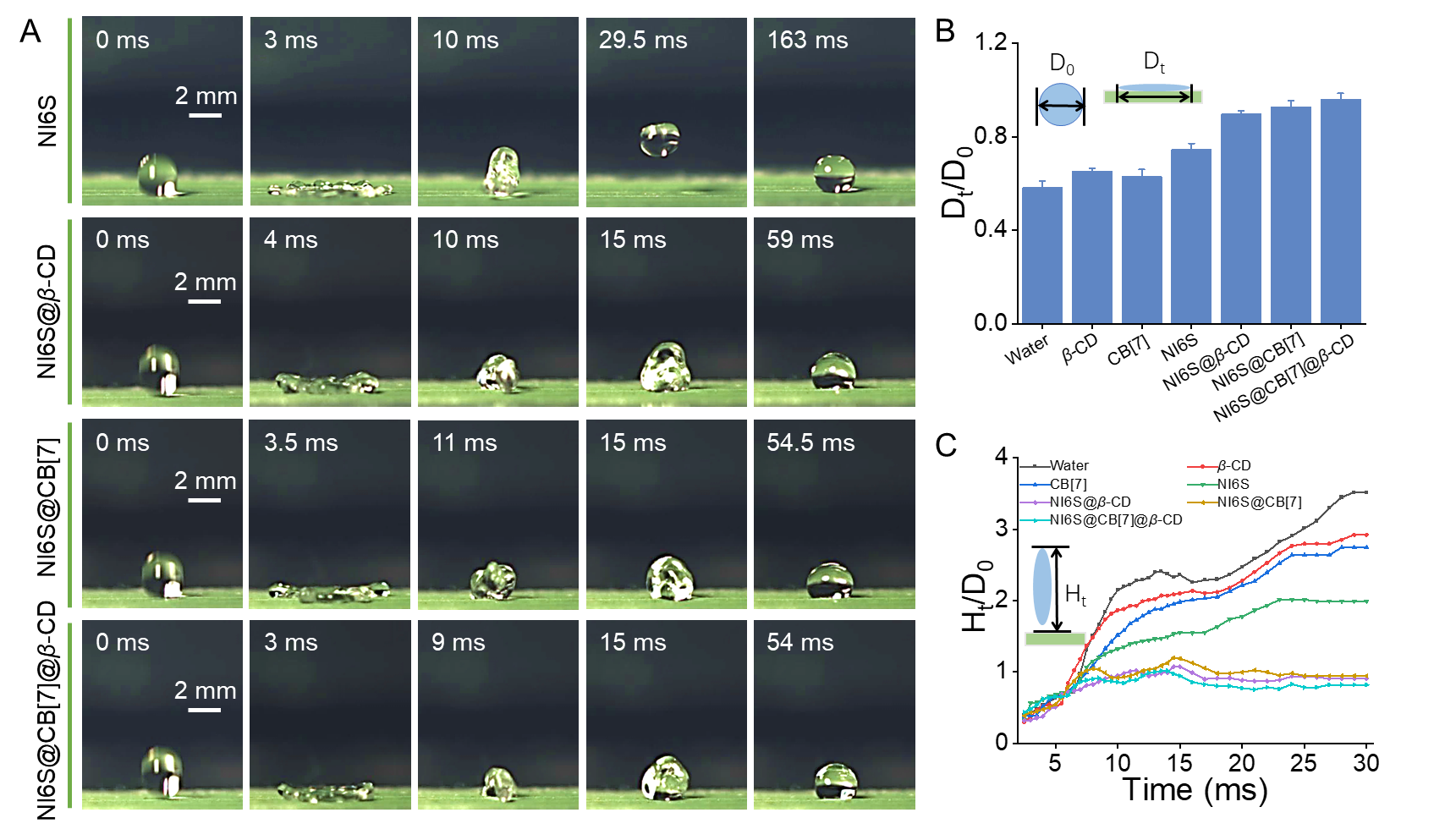


**Figure S31.** A) Bouncing behavior of NI6S, NI6S@*β*-CD, NI6S@CB[7] and NI6S@CB[7]@*β*-CD droplets on rice leaves (height: 10 cm). B) Normalized rebound height (H_t_/D_0_) diagram of each component, D_0_ and H_t_ represent the initial diameter of the droplet and the rebound height from the tip of the droplet to the rice surface during the shrinkage process, respectively. C) Normalized diffusion diameter (D_t_/D_0_) diagram of droplet stability of each component, D_t_ represents the spreading diameter of the droplet when it falls on the surface of rice leaves.

##
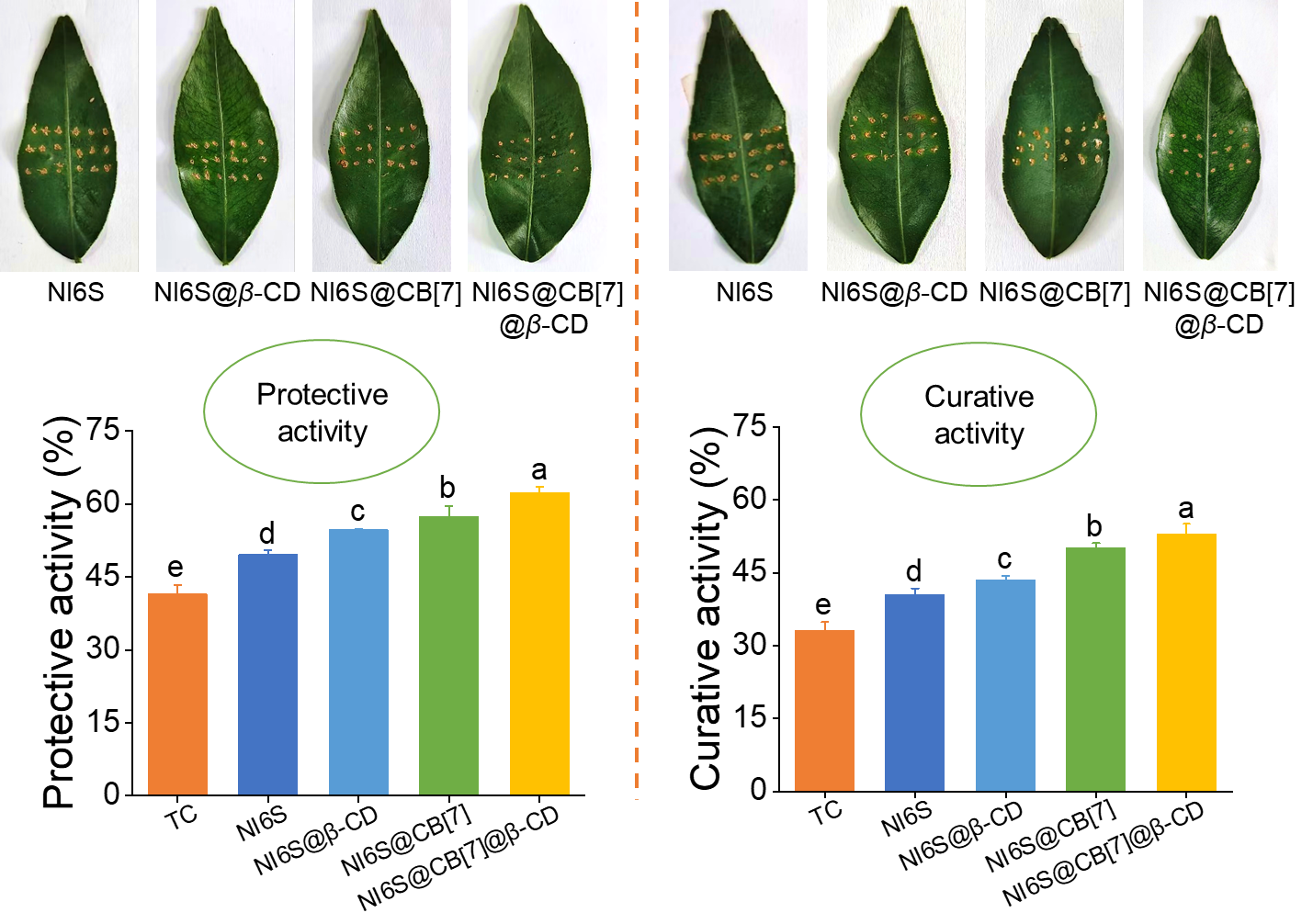
4.17 *In Vivo* Control of NI6S@CB[7]@*β*-CD on *Xac*

**Figure S32.** Protective activity and curative activity of NI6S, NI6S@*β*-CD, NI6S@CB[7], NI6S@CB[7]@*β*-CD and TC-20%SC against citrus bacterial canker at 200 *μ*g mL^-1^ concentration (14 d after spraying) and corresponding control effects.

## 4.18 *In Vivo* Control of NI6S@CB[7]@*β*-CD on *Psa*


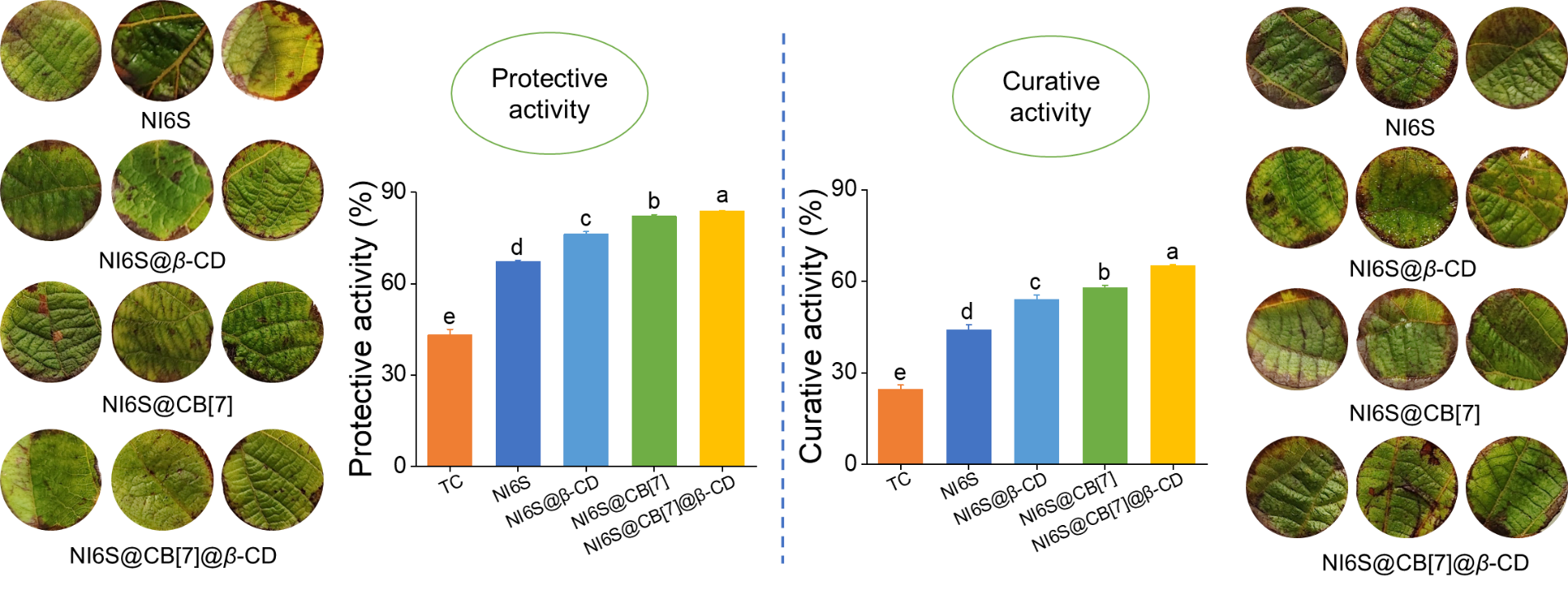


**Figure S33.** Effects of NI6S, NI6S@*β*-CD, NI6S@CB[7], NI6S@CB[7]@*β*-CD and TC-20%SC at 200 *μ*g mL^-1^ concentration (5 days after spraying) on the curative and protective activities of kiwifruit bacterial canker and corresponding control effects.

## 4.19 Phytotoxicity Studies of NI6R, NI6R@*β*-CD, NI6R@CB[7] and NI6R@CB[7]@*β*-CD on Rice Plants


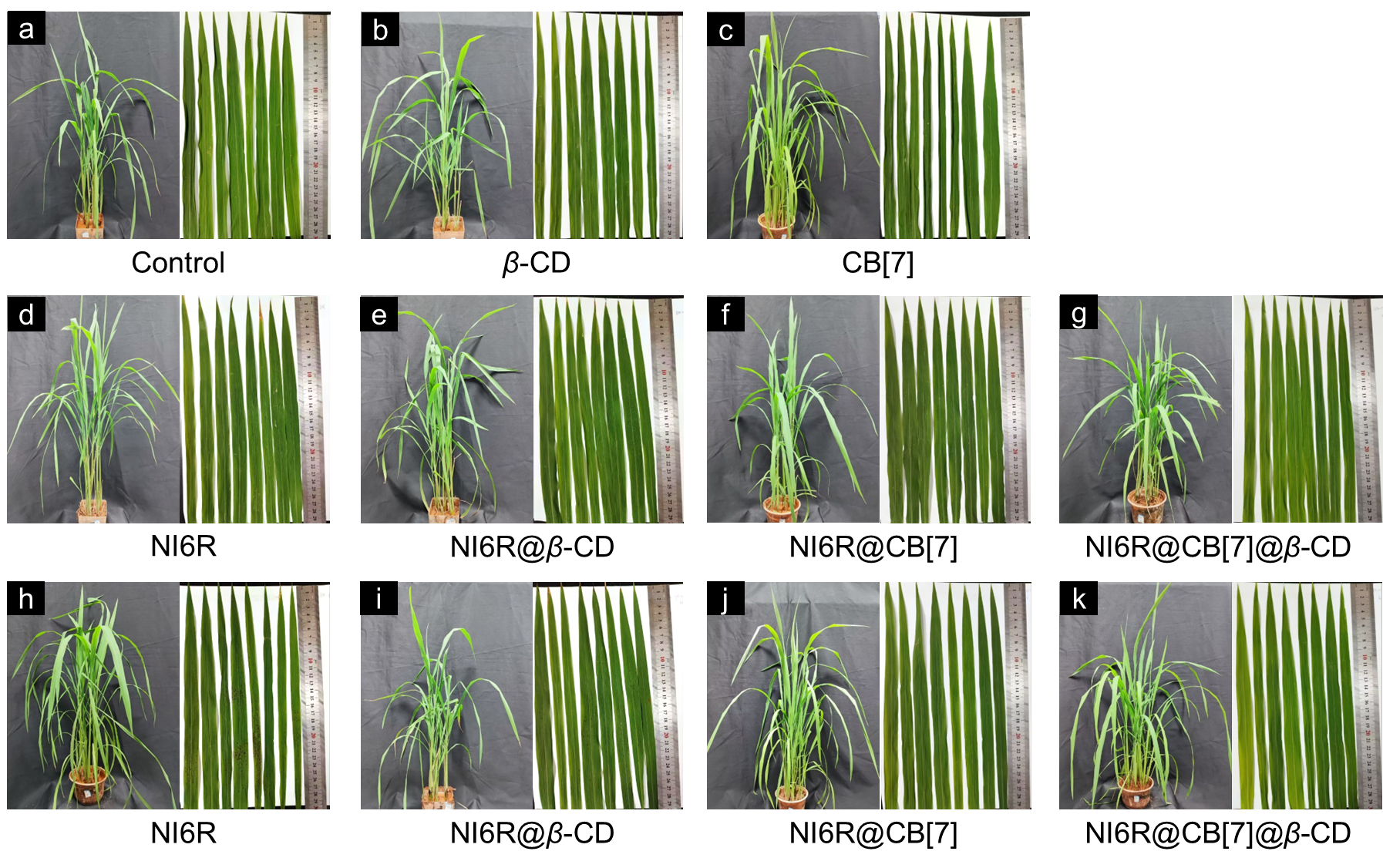


**Figure S34.** Phytotoxicity studies of NI6R, NI6R@*β*-CD, NI6R@CB[7] and NI6R@CB[7]@*β*-CD on rice plants for 7 d. The bactericide concentration was 200 *μ*g mL^-1^ for (d-g) and 500 *μ*g mL^-1^ for (h-k).

## 4.20 Acute Toxicity of NI6R, NI6R@*β*-CD, NI6R@CB[7] and NI6R@CB[7]@*β*-CD to Earthworm and Zebrafish


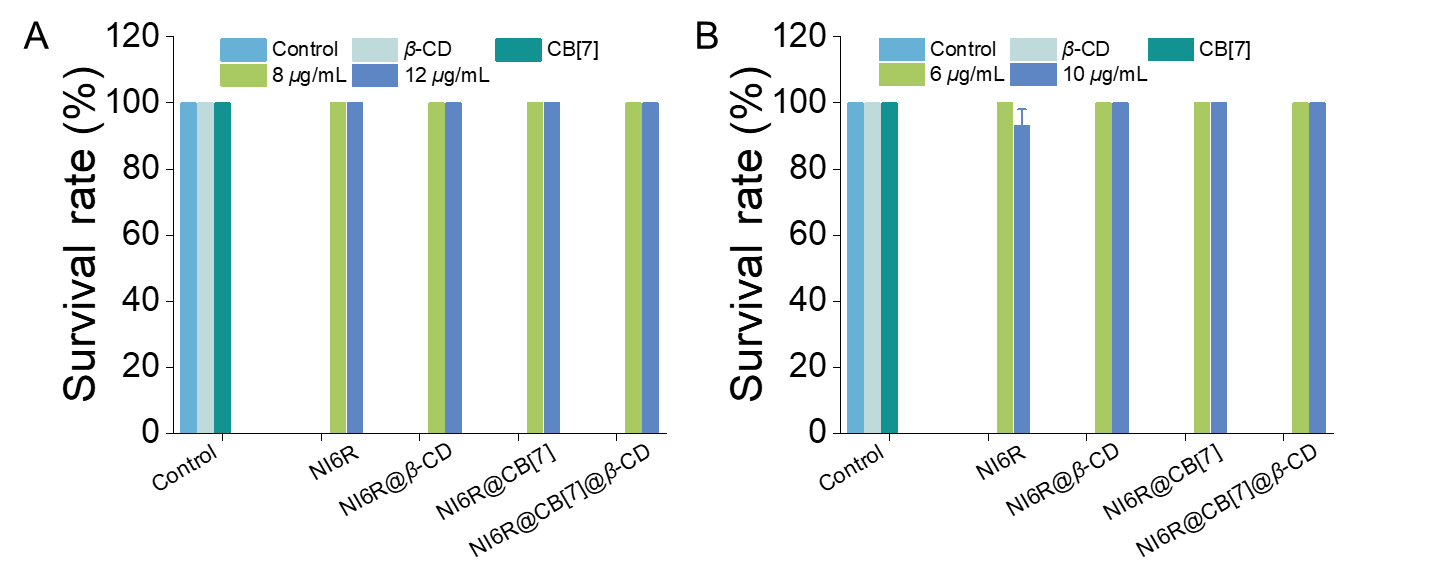


**Figure S35.** A) Survival rate of earthworms treated with 8 and 12 *μ*g mL^-1^ concentrations of NI6R, NI6R@*β*-CD, NI6R@CB[7] and NI6R@CB[7]@*β*-CD. B) Survival rate of zebrafishes treated with 6 and 10 *μ*g mL^-1^ concentrations of NI6R, NI6R@*β*-CD, NI6R@CB[7] and NI6R@CB[7]@*β*-CD.

# 5. Supplementary Tables

## 5.1 NI6R/S against Plant Pathogens *Xoo*

**Table S1.** The bactericidal activity of compounds NI6R and NI6S against *Xoo* with different concentration gradients.

| Compounds | Inhibition ratio (%) | | | | | | |
| --- | --- | --- | --- | --- | --- | --- | --- |
|  | 50  *μ*g mL^-1^ | 25  *μ*g mL^-1^ | 12.5  *μ*g mL^-1^ | 6.25  *μ*g mL^-1^ | 3.0  *μ*g mL^-1^ | 1.5  *μ*g mL^-1^ | 0.75  *μ*g mL^-1^ |
| NI6R | 100 | 100 | 100 | 100 | 100 | 64.9±1.3 | 18.9±1.2 |
| NI6S | 100 | 100 | 100 | 85.9±0.9 | 21.5±1.1 | 7.6±1.7 | - |

## 5.2 Chemical Shift Change of Supramolecular Complex NI6R@CB[7] and NI6R@CB[7]@*β*-CD After Assembly

**Table S2.** Chemical shift of NI6R after the formation of supramolecular complex NI6R@CB[7] with different molar ratios.


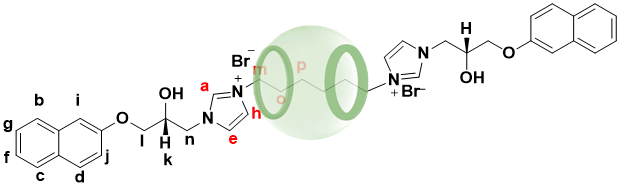


| Chemical shift (ppm) | Protons | | | | | | | | |
| --- | --- | --- | --- | --- | --- | --- | --- | --- | --- |
|  | H_a_ | H_e_ | H_h_ | H_k_ | H_l_ | H_m_ | Hn | H_o_ | H_p_ |
| NI6R | 8.4216 | 7.4548 | 7.2612 | 4.4087 | 4.4087 | 3.8576 | 3.7426 | 1.1740 | 0.5238 |
| NI6R: CB[7]=1:0.5 | 8.4980 | 7.4527 | 7.2617 | 4.2919 | 4.4075 | 3.3064 | 3.7409 | 0.8772 | 0.3767 |
| NI6R: CB[7]=1:1 | 8.5003 | 7.4264 | 7.2969 | 4.3286 | 4.3995 | 3.3059 | 3.6700 | 0.8775 | 0.3780 |
| Shift _NI6R: CB[7]=1:0.5_ | 0.076 | -0.002 | 0.001 | -0.117 | -0.001 | -0.551 | -0.002 | -0.297 | -0.147 |
| Shift _NI6R: CB[7]=1:1_ | 0.079 | -0.028 | 0.036 | -0.081 | -0.009 | -0.552 | -0.073 | -0.297 | -0.146 |

**Table S3.** Chemical shift of NI6R after the formation of supramolecular complex NI6R@*β*-CD with different molar ratios.


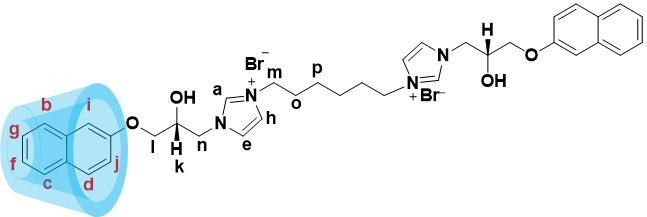


| Chemical shift (ppm) | Protons | | | | | | | | |
| --- | --- | --- | --- | --- | --- | --- | --- | --- | --- |
|  | H_b,c_ | H_d_ | H_e_ | H_f_ | H_g_ | H_h_ | H_i_ | H_j_ |  |
| NI6R | 7.7687 | 7.7033 | 7.4548 | 7.4253 | 7.3462 | 7.2587 | 7.0803 | 7.0629 |  |
| NI6R:*β*-CD=1:0.5 | 7.7536 | 7.6959 | 7.4616 | 7.4129 | 7.3253 | 7.2635 | 7.0934 | 7.0685 |  |
| NI6R:*β*-CD=1:1 | 7.7465 | 7.6919 | 7.4688 | 7.4054 | 7.3166 | 7.2642 | 7.1064 | 7.0816 |  |
| Shift _NI6R:_*_β_*_-CD=1:0.5_ | -0.015 | -0.007 | 0.007 | -0.012 | -0.021 | 0.005 | 0.013 | 0.006 |  |
| Shift _NI6R:_*_β_*_-CD=1:1_ | -0.022 | -0.011 | 0.014 | -0.020 | -0.030 | 0.006 | 0.026 | 0.019 |  |

**Table S4.** Chemical shift of NI6R@CB[7] after the formation of supramolecular complex NI6R@CB[7]@*β*-CD with different molar ratios.


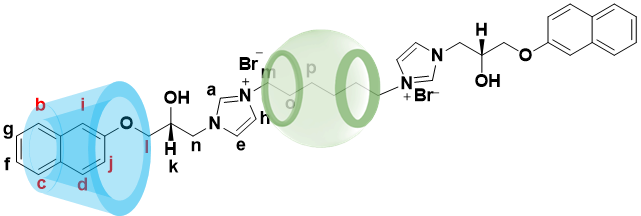


| Chemical shift (ppm) | Protons | | | | | | | | |
| --- | --- | --- | --- | --- | --- | --- | --- | --- | --- |
|  | H_b,c_ | H_d_ | H_f_ | H_g_ | H_i_ | H_j_ | H_k_ | H_l_ |  |
| NI6R:CB[7]=1:1 | 7.8847 | 7.7508 | 7.5306 | 7.4558 | 7.4163 | 7.2882 | 4.3134 | 4.4535 |  |
| NI6R:CB[7]:*β*-CD=1:1:0.5 | 7.8774 | 7.7641 | 7.5461 | 7.4556 | 7.4012 | 7.3273 | 4.3585 | 4.4408 |  |
| NI6R:CB[7]:*β*-CD=1:1:1 | 7.8810 | 7.7708 | 7.5598 | 7.4616 | 7.4057 | 7.3332 | 4.3611 | 4.4296 |  |
| Shift _NI6R:CB[7]:_*_β_*_-CD=1:1:0.5_ | -0.007 | 0.013 | 0.016 | -0.0002 | -0.015 | 0.039 | 0.045 | -0.013 |  |
| Shift _NI6R:CB[7]:_*_β_*_-CD=1:1:1_ | -0.004 | 0.020 | 0.029 | 0.006 | -0.011 | 0.045 | 0.048 | -0.024 |  |

## 5.3 Efficiency of *in Vivo* Control of Rice Bacterial Leaf Blight

**Table S6.** *In vivo* control efficiency of NI6R, NI6R@*β*-CD, NI6R@CB[7], NI6R@CB[7]@*β*-CD, NI6S, NI6S@*β*-CD, NI6S@CB[7], NI6S@CB[7]@*β*-CD and TC-20%SC against rice bacterial leaf blight at 200 *μ*g mL^-1^.

|  | | Protective activity | | | Curative activity | | |
| --- | --- | --- | --- | --- | --- | --- | --- |
| Compounds | | Morbidity  (%) | Disease  index (%) | Control  efficiency (%) | Morbidity  (%) | Disease  index (%) | Control  efficiency (%) |
| NI6R | | 100 | 55.24±0.68 | 40.34±0.71d | 100 | 56.19±0.85 | 38.80±1.46cd |
| NI6R@*β*-CD | | 100 | 51.43±0.47 | 43.42±0.75c | 100 | 53.33±1.80 | 41.72±1.43c |
| NI6R@CB[7] | | 100 | 50.67±0.56 | 45.28±0.84b | 100 | 51.90±0.98 | 43.49±0.54b |
| NI6R@*β*-CD@CB[7] | | 100 | 46.67±1.13 | 49.59±0.80a | 100 | 48.70±0.59 | 46.98±0.93a |
| NI6S | 100 | 56.19±0.75 | 39.31±0.58e | 100 | 57.14±1.16 | 37.79±0.76d |  |
| NI6S@*β*-CD | | 100 | 54.28±0.37 | 41.37±0.86cd | 100 | 54.28±1.75 | 40.90±1.39c |
| NI6S@CB[7] | | 100 | 52.86±0.93 | 42.91±1.46cd | 100 | 55.71±1.28 | 39.35±0.83cd |
| NI6S@*β*-CD@CB[7] | | 100 | 49.33±0.91 | 46.72±0.53b | 100 | 50.67±0.90 | 44.83±0.48b |
| TC^b^ | | 100 | 61.48±1.60 | 33.59±1.18f | 100 | 64.44±0.79 | 29.84±0.78e |
| *β*-CD | | 100 | 91.11±1.18 | 1.60±0.45g | 100 | 89.63±0.94 | 2.42±0.65f |
| Control^a^ | | 100 | 92.59±0.78 | - | 100 | 91.85±0.86 | - |

Note: a) Negative control; b) Commercial bactericidal agents as positive controls. Abbreviation: TC, thiodiazole-copper. Different lowercase letters indicate significant differences in control efficiency between treatment groups at *P*<0.05. Statistical significance was analyzed using IBM SPSS Statistics 27 software.

## 5.4 NI6R/S against Plant Pathogens *Xac* and *Psa*

**Table S7.** *In vitro* bactericidal activities of title compounds NI6R/S against plant pathogens *Xac* and *Psa*.

| Compounds | *Xac* | | *Psa* | |
| --- | --- | --- | --- | --- |
|  | Regression Equation | EC_50_ ^a^ (*μ*g mL^-1^) | Regression Equation | EC_50_^a^ (*μ*g mL^-1^) |
| NI6R | y=6.98x+4.86 | 1.05±0.05 | y=5.09x+4.66 | 1.16±0.06 |
| NI6S | y=6.82x+3.12 | 1.88±0.02 | y=6.36x+3.27 | 1.87±0.08 |
| BT^b^ | y=1.36x+2.34 | 91.1±7.10 | y=1.63x+1.64 | 116±3.66 |
| TC^b^ | y=4.34x-4.37 | 144±5.78 | y=2.34x+0.0590 | 131±0.93 |

Note: a) EC_50_ value of bactericidal activities are indicated as means ± SD (standard deviation); b) Commercialized bactericidal agents as positive controls. Abbreviations: BT, bismerthiazol; TC, thiodiazole-copper.

# References

[1] H. Dai, J. Yang, L. Fan, M. Luo, P. Wang, *Adv. Funct. Mater.* **2024**, *34*, 2403823.

[2] Y. Liu, M. Zheng, Z. Xie, *Chem. Eng. J.* **2024**, *486*, 150361.

[3] J. Tang, X. Tong, Y. Chen, Y. Wu, Z. Zheng, A. B. Kayitmazer, A. Ahmad, N. Ramzan, J. Yang, Q. Huang, Y. Xu, *Nat. Commun.* **2023**, *14*, 6401.

[4] W. B. Shao, P. Y. Wang, Z. M. Fang, J. J. Wang, D. X. Guo, J. Ji, X. Zhou, P. Y. Qi, L. W. Liu, S. Yang, *J. Agric. Food Chem.* **2021**, *69*, 15108.

[5] H. W. Liu, Q. T. Ji, G. G. Ren, F. Wang, F. Su, P. Y. Wang, X. Zhou, Z. B. Wu, Z. Li, S. Yang, *J. Agric. Food Chem.* **2020**, *68*, 12558.

[6] L. Jiang, L. Luo, M. Li, T. Xie, Z. Zhao, C. Kang, D. Chen, Y. Long, *Microchem. J.* **2023**, *192*, 108955.

[7] L. Yang, H. Chen, S. Zhu, S. Zhao, S. Huang, D. Cheng, H. Xu, Z. Zhang, *ACS Nano* **2024**, *18*, 6533.

[8] S. A. A. Abdel-Raheem, M. R. Fouad, M. A. Gad, A. M. Kamal El-Dean, M. S. Tolba, *J. Environ. Chem. Eng.* **2023**, *11*, 110839.

[9] S. M. Kim, J. P. Suh, Y. Qin, T. H. Noh, R. F. Reinke, K. K. Jena, *Theor. Appl. Genet.* **2015**, *128*, 1933.

[10] S. S. Muhammad, Y. Liu, M. Li, H. J. Wang, R. Y. Su, C. L. Wang, Z. Y. Ji, *Plant Biotechnol. J.* **2024**, *22*, 2632.
